# Functionalized Congeners of 2*H*-Chromene P2Y_6_ Receptor Antagonists

**DOI:** 10.3390/cells13161366

**Published:** 2024-08-16

**Authors:** Paola Oliva, Asmita Pramanik, Young-Hwan Jung, Sarah A. Lewicki, Jamie M. Mwendwa, Jong Hwan Park, Kenneth A. Jacobson

**Affiliations:** Molecular Recognition Section, Laboratory of Bioorganic Chemistry, National Institute of Diabetes and Digestive and Kidney Diseases, National Institutes of Health, Bethesda, MD 20892, USA; paola.oliva@nih.gov (P.O.); asmita.pramanik@nih.gov (A.P.); jyh0401@gmail.com (Y.-H.J.); sarahlewicki2021@gmail.com (S.A.L.); jamiemikali@gmail.com (J.M.M.); jonghwan.park@nih.gov (J.H.P.)

**Keywords:** P2Y receptor, antagonist, nucleotide, GPCR, calcium, structure–activity relationship

## Abstract

The P2Y_6_ receptor (P2Y_6_R), a G_q_-coupled receptor, is a potential drug discovery target for various inflammatory and degenerative conditions. Antagonists have been shown to attenuate colitis, acute lung injury, etc. In the search for competitive antagonists, we have investigated the SAR of 3-nitro-2-(trifluoromethyl)-2*H*-chromene derivatives, although high affinity is lacking. We now reveal that long-chain amino-functionalized congeners display greatly enhanced affinity in the antagonism of UDP-induced Ca^2+^ mobilization in *human* (h) P2Y_6_R-transfected 1321N1 astrocytoma cells. A 6-(Boc-amino-*n*-heptylethynyl) analogue **30** (MRS4940) had an IC_50_ of 162 nM, which was a 123-fold greater affinity than the corresponding unprotected primary alkylamine, 107-fold greater than the corresponding pivaloyl derivative **30**, and 132-fold selective compared to the P2Y_14_R. However, similar Boc-amino chains attached at the 8-position produced weak µM affinity. Thus, the P2Y_6_R affinity depended on the chain length, attachment point, and terminal functionality. Off-target activities, at 45 sites, were tested for acylamino derivatives **20**, **24**, **26**, **30**, **31**, and **37**, which showed multiple interactions, particularly at the biogenic amine receptors. The more potent analogues may be suitable for evaluation in inflammation and cancer models, which will be performed in future studies.

## 1. Introduction

The P2Y_6_ receptor (P2Y_6_R) responds to extracellular uridine 5′-diphosphate (UDP) to activate phospholipase C β (PLCβ) and subsequently Ca^2+^ mobilization through the G_q/11_ subfamily of heterotrimeric GTP-binding proteins [1]. UDP is a short-lived, local proinflammatory danger signal, which is cleaved to inactive products by ectonucleotidases [2,3], such as intestinal nucleoside triphosphate diphospho-hydrolase (NTPDase)8 [4]. The P2Y_6_R occurs in microglia, macrophages, T cells, cardiomyocytes, endothelial cells, and other cell types [5,6,7,8,9], causing the release of inflammatory mediators, such as interleukins IL-6 and IL-8. P2Y_6_R antagonists are sought for diverse conditions, including diabetes and obesity, pain, cancer, colitis, atherosclerosis, memory loss in aging, psoriasis, and others [8,9,10,11]. P2Y_6_R knockout (KO) mice, either whole-body or tissue-specific, have been utilized to probe the role of this receptor in various disease models. Both beneficial [4,7,12,13,14] and detrimental effects [15,16,17,18] of genetic knockout (KO) or receptor antagonism have been found.

**Figure 1:**
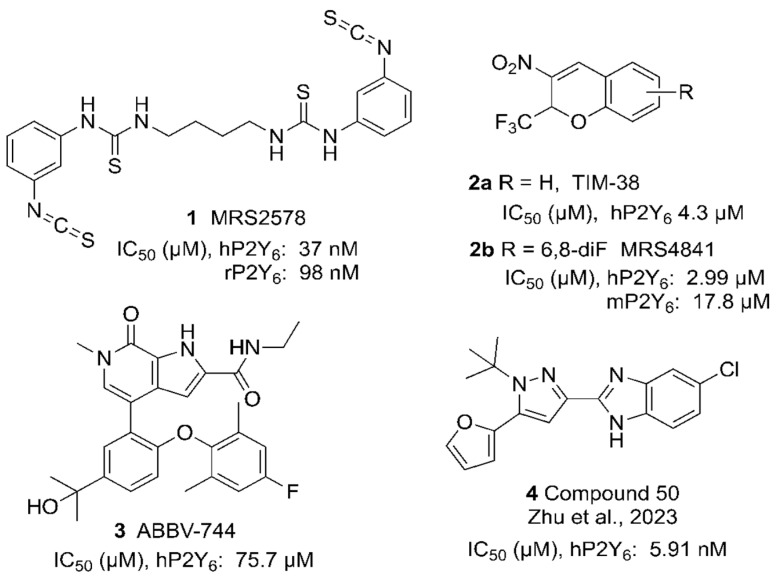
Selected P2Y_6_R antagonist structures, with the IC_50_ values in inhibition of UDP-induced Ca^2+^ mobilization in hP2Y_6_R-expressing 1321N1 astrocytoma cells (h, *human*; r, *rat*; m, *mouse*) [33].

We have also explored in detail the structure–activity relationship (SAR) of UDP and CDP derivatives as P2Y_6_R agonists and separated their ability to activate the P2Y_6_R from activity at the G_i_-coupled P2Y_14_R, which is also UDP-activated [19]. However, the discovery of potent and selective P2Y_6_R antagonists has lagged behind other purinergic GPCRs, although numerous studies have suggested their potential utility as a drug discovery target [1,4,5,6,8,20,21,22,23,24,25,26,27,28]. Only a few classes of P2Y_6_R antagonists have been explored. The first selective antagonists were in a series of di-arylisothiocyanates, among which MRS2578 (Figure 1) is the most widely used. This compound does not bind in or near the orthosteric binding site but rather binds covalently to a Cys residue in the intracellular receptor region [6]. This binding induces a mechanism called “redox-induced degradation” by which the receptor is driven to the proteasome for degradation. Another class of P2Y_6_R antagonists was identified through library screening to discover a 2*H*-chromene derivative **2a** as a reversible P2Y_6_R antagonist of µM affinity, and its SAR was initially explored by Ito et al. [29]. We have further mapped the SAR, focusing on 6- and 8-position substitutions, e.g., 6,8-difluoro **2b** [30,31]. These antagonists contain an alkyl nitro group and are racemic, characteristics that are considered less than optimal for drug development. Nevertheless, we have gradually improved this series’ affinity, including in this work. Machine learning is an alternative approach that was used to identify ABBV-744 **3** as a non-surmountable P2Y_6_R antagonist [32]. Recently, a 2-(1*H*-pyrazol-3-yl)-1*H*-benzo[*d*]imidazole derivative **4** was described as a potent P2Y_6_R antagonist that attenuated colitis and acute lung injury [33]. 

This SAR study of the 2*H*-chromene class of P2Y_6_R antagonists applies a functionalized congener design approach [34]. Homologous ω-aminoalkyl-functionalized congeners, and their acylated derivatives, of adenosine receptor (AR) ligands were first reported by Jacobson et al. in 1985 as a model for other GPCRs [35]. Strategically chain-extended orthosteric AR agonists and antagonists had enhanced affinity when a terminal amine was present [35]. A later X-ray structure of the A_2A_AR complex with a xanthine amine congener (XAC) featured its aminoalkyl chain facing outward from the orthosteric binding site and interacting with polar residues [36], as envisioned in an earlier design [35]. A series of aminoalkyl congeners of the M_1_ muscarinic acetylcholine receptor antagonist telenzepine showed an affinity optimum with a 10-methylene length [37]. We now empirically apply a similar strategy for the P2Y_6_R, for which there is no experimental structure, as a means of indirectly probing the binding site environment. We considered that the functionalized chains appended might extend into the phospholipid bilayer, as was shown for the A_3_AR [38].

**Figure 2:**
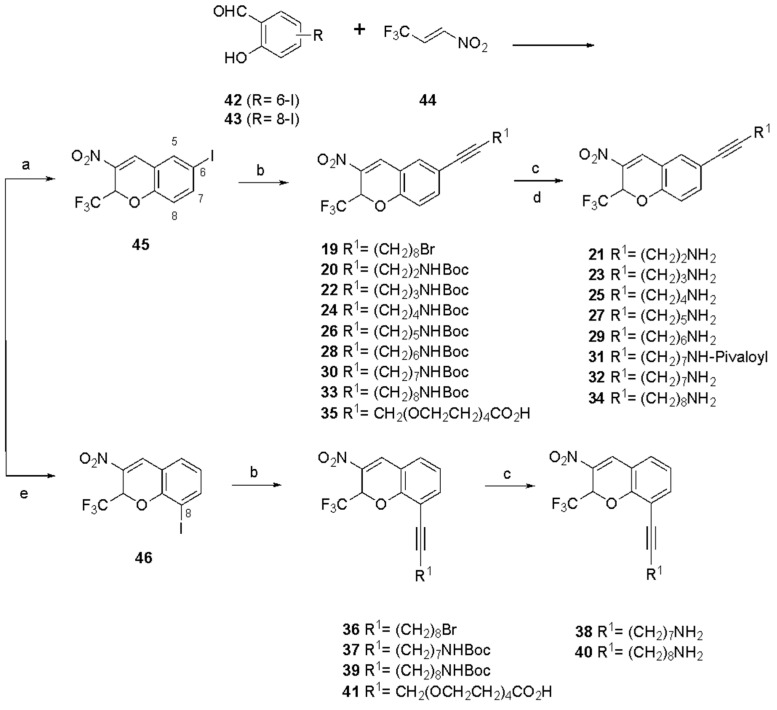
Synthetic route to P2Y_6_R antagonists (compounds **19**–**41**). Reagents and conditions: (**a**) TEA, DCM, rt, 4 h, and 51–89%; (**b**) alkyne R^1^-H, PdCl_2_(PPh_3_)_2_, CuI, TEA, DMF, rt, 7 h, and 58–99%; (**c**) TFA, DCM, rt, and 3 h; (**d**) pivaloyl chloride, TEA, DMF, rt, and 3 h; and (**e**) DBU, DMF, *μ*W, 80 °C, 2 h, and 32–99%.

The list of 2*H*-chromene analogues compared here for *human* (h) P2Y_6_R functional inhibition is shown in Table 1, including reference compounds **2** and **4**–**18**. The primary pharmacological assay consisted of the calcium mobilization measurement in hP2Y_6_R-expressing 1321N1 astrocytoma cells using a fixed UDP (agonist) concentration of 100 nM [30,31]. The newly prepared derivatives (**19**–**41**) were synthesized as shown in Figure 2, with the full synthetic procedures and spectral characterization in the Supplementary Materials. The derivatization concentrated on the 6 and 8 positions of original hit compound **2a** using 6-I and 8-I intermediates (**10** and **11**, respectively) subjected to Sonogashira reactions to install various extended alkyne chains, including those bearing a Boc-amino terminal group. The Boc protection was removed using TFA to yield the corresponding alkyl amines. 

## 2. Materials and Methods

The representation synthetic methods for compounds **24**, **30**, and **36** are presented here (Figure 2), and the methods for the other compounds are found in the Supplementary Materials (Figure S1). 

### 2.1. Materials

Unless noted, the reagents and solvents were purchased from Sigma-Aldrich (St. Louis, MO, USA). The anhydrous solvents were obtained directly from commercial sources. The Boc-amino-alkynes and other alkynes were obtained from Ambeed (Arlington Heights, IL, USA), except for *N*-Boc-non-8-yn-1-amine, *N*-Boc-9-decyn-1-amine, and 10-Br-1-decyne, which were purchased from Acrotein (Hoover, AL, USA). All the reactions were performed using anhydrous solvents and under dry nitrogen. Room temperature or rt refers to 25 ± 2 °C. The ^1^H-NMR spectra were obtained with a Bruker 400 MHz spectrometer in CDCl_3_ (7.26 ppm) and MeOD (3.31 ppm). The chemical shifts are expressed as ppm downfield and coupling constants (*J*) are given in Hz. The TLC analysis utilized glass sheets precoated with silica gel F254 (0.2 mm) from Aldrich. The purity of the final compounds (**19**–**41**) was checked using a Hewlett−Packard 1100 HPLC (Agilent Technologies Inc., Palo Alto, CA, USA) equipped with an Agilent Eclipse 5 µm XDB-C18 analytical column (50 × 4.6 mm); the mobile phase: a linear gradient solvent system, 10 mM EAA (triethylammonium acetate):CH_3_CN from 95:5 to 0:100 in 20 min; and the flow rate was 1.0 mL/min. UV absorption peaks (230, 254, and 280 nm) were detected using a diode array detector. All the derivatives later evaluated pharmacologically showed >95% purity by analytical HPLC. Low-resolution mass spectrometry was performed with a JEOL SX102 spectrometer with 6 kV Xe atoms following desorption from a glycerol matrix or on an Agilent LC/MS 1100 MSD, with an Atlantis C18 column (Waters, Milford, MA, USA). High-resolution mass spectroscopic (HRMS) measurements were carried out on a proteomics-optimized Q-TOF-2 (Micromass-Waters) using external polyalanine calibration and observation of mass accuracies.

### 2.2. Chemical Synthetic Procedures

*tert*-Butyl (6-(3-nitro-2-(trifluoromethyl)-2*H*-chromen-6-yl)hex-5-yn-1-yl)carbamate (**24**). The compound was synthesized following the same procedure for compound **36**, using *N*-Boc-hex-5-yn-1-amine as the alkyne. The residue was purified by silica gel column chromatography (hexane:ethyl acetate = 90:10) to afford compound **24** (57% yield) as a yellow solid; ^1^H NMR (400 MHz, methanol-*d*_4_) δ 8.32 (d, *J* = 2.1 Hz, 1H), 7.54 (d, *J* = 1.9 Hz, 1H), 7.52–7.43 (m, 1H), 7.02 (d, *J* = 8.4 Hz, 1H), 6.35 (qd, *J* = 6.5, 1.6 Hz, 1H), ESI-HRMS calcd. *m*/*z* for C_16_H_16_N_2_O_3_F_3_ 341.1113, found 341.1108.

*tert*-Butyl (9-(3-nitro-2-(trifluoromethyl)-2*H*-chromen-6-yl)non-8-yn-1-yl)carbamate (**30**). The compound was synthesized following the same procedure for compound **36**, using *N*-Boc-non-8-yn-1-amine as the alkyne. The residue was purified by silica gel column chromatography (hexane:ethyl acetate = 90:10) to afford the compound **30** (83% yield) as a yellow solid; ^1^H NMR (400 MHz, CDCl_3_) δ 8.05 (s, 1H), 7.44 (dd, *J* = 8.5, 2.0 Hz, 1H), 7.38 (d, *J* = 2.0 Hz, 1H), 6.98 (d, *J* = 8.4 Hz, 1H), 6.07 (q, *J* = 6.2 Hz, 1H), 4.52 (s, 1H), 3.10 (h, *J* = 6.3, 5.2 Hz, 4H), 2.37 (t, *J* = 7.0 Hz, 2H), 2.23 (t, *J* = 6.9 Hz, 2H), 1.58 (p, *J* = 7.1 Hz, 2H), 1.43 (s, 9H), 1.38–1.23 (m, 4H). MS (ESI, *m*/*z*) 505.2 [M+H]^+^; ESI-HRMS calcd. *m*/*z* for C_24_H_29_N_2_O_5_F_3_ 505.1926, found 505.1932 [M + H]^+^.

8-(10-Bromodec-1-yn-1-yl)-3-nitro-2-(trifluoromethyl)-2*H*-chromene (**36**). To a mixture of 8-iodo-3-nitro-2-(trifluoromethyl)-2H-chromene (**46**) (50 mg, 0.134 mmol), PdCl_2_(PPh_3_)_2_ (4.7 mg, 0.0067 mmol), and copper iodide (2.5 mg, 0.0134 mmol) in DMF (3 mL), triethylamine (37 μL, 0.27 mmol) and 10-bromodec-1-yne (44 mg, 0.202 mmol) were added at room temperature under a N_2_ atmosphere, and then this reaction mixture was stirred at room temperature for 7 h under a N_2_ atmosphere. This reaction mixture was diluted with water (5 mL) and extracted with ethyl acetate (10 mL × 3). The combined organic extracts were dried over anhydrous Na_2_SO_4_, filtered through a pad of Celite, and evaporated under reduced pressure. The residue was purified by silica gel column chromatography (hexane:ethyl acetate = 90:10) to afford the compound **36** (32 mg, 52%) as a yellow solid; ^1^H NMR (400 MHz, CDCl_3_) δ 8.10 (s, 1H), 7.50 (dd, *J* = 8 Hz, 1H), 7.28 (dd, *J* = 8 Hz, 1H), 7.03 (t, *J* = 8 Hz, 1H), 6.14–6.21 (m, 1H), 3.41 (t, *J* = 8 Hz, 2H), 2.46 (t, *J* = 8 Hz, 2H), 1.81–1.90 (m, 2H), 1.45–1.51 (m, 8H).

6-Iodo-3-nitro-2-(trifluoromethyl)-2*H*-chromene (**45**). To a mixture of 5-iodosalicylaldehyde (**42**, 0.50 g, 2.01 mmol) and (1*E*)-3,3,3-trifluoro-1-nitroprop-1-ene (**44**, 0.31 g, 2.21 mmol) in dichloromethane (10 mL), triethylamine (42 μL, 0.30 mmol) was added. The mixture was stirred at room temperature for 5 h. This reaction mixture was diluted with water (20 mL) and extracted with dichloromethane (10 mL × 3). The combined organic extracts were dried over anhydrous Na_2_SO_4_, filtered, and evaporated under reduced pressure. The residue was purified by silica gel column chromatography (hexane:ethyl acetate = 20:1) to afford the compound **45** (0.57 g, 77%) as a yellow solid; HPLC purity 99%; ^1^H NMR (400 MHz, CDCl_3_) δ 8.00 (s, 1H), 7.70 (dd, *J* = 2.12 and 8.56 Hz, 1H), 7.65 (d, *J* = 2.12 Hz, 1H), 6.84 (d, *J* = 8.56 Hz, 1H), 6.06 (q, *J* = 6.16 Hz, 1H); ^19^F NMR (376 MHz, CDCl_3_) δ −77.69 (d, *J* = 6.16 Hz, CF_3_). 

8-Iodo-3-nitro-2-(trifluoromethyl)-2*H*-chromene (**46**). To a mixture of 2-hydroxy-3-iodobezaldeyde (**43**, 20 mg, 0.142 mmol) and (1E)-3,3,3-trifluoro-1-nitroprop-1-ene (**44**, 90 mg, 0.642 mmol) in DMF (2.0 mL), 1,8-diazabicyclo[5.4.0]undec-7-ene (2.0 μL, 0.014 mmol) was added. The mixture was stirred at 80 °C for 2 h in a microwave reactor. This reaction mixture was diluted with water (5 mL) and extracted with ethyl acetate (10 mL × 3). The combined organic extracts were dried over anhydrous Na_2_SO_4_, filtered, and evaporated under reduced pressure. The residue was purified by silica gel column chromatography (hexane:ethyl acetate = 95:5) to afford the compound **46**. Yield = 41%; HPLC purity 99%; ^1^H NMR (400 MHz, CDCl_3_) δ 8.06 (s, 1H), 7.87 (dd, *J* = 1.48 and 7.92 Hz, 1H), 7.35 (dd, *J* = 1.48 and 7.56 Hz, 1H), 6.88 (t, *J* = 7.72 Hz, 1H), 6.18 (q, *J* = 6.16 Hz, 1H); ^19^F NMR (376 MHz, CDCl_3_) δ −77.78 (d, *J* = 6.12 Hz, 3F).

### 2.3. Assay of hP2Y_6_R-Induced Ca^2+^ Transients

To screen for P2Y_6_R antagonist activity, PLC-mediated calcium mobilization assays were carried out using the recombinant *human* P2Y_6_R stably expressed in 1321N1 astrocytoma cells (hP2Y_6_R-1321N1) according to the previously published method [30]. Briefly, the hP2Y_6_R-1321N1 cells were seeded on 96-well black plates and incubated overnight. The antagonists were pretreated along with the FLIPR dye (Calcium 6 assay kit, Molecular Devices, San Jose, CA, USA) containing 5 mM probenecid for 45 min, prior to agonist addition followed by an immediate intracellular Ca^2+^ measurement. UDP was used as a P2Y_6_R agonist (final concentration 100 nM, EC_50_ 13 nM in PLC activation). Here, probenecid was used as an organic anion transporter inhibitor to prevent the efflux of the dye into the extracellular environment. During incubation, the membrane permeable dye enters the cytoplasm and cleaves the acetoxymethyl protection to enable Ca^2+^ binding. When the P2Y_6_R was activated by UDP, the increased calcium concentration was directly measured by intracellular changes in the fluorescence intensity with a Fluorescent Imaging Plate Reader (FLIPR^TETRA^, Molecular Devices, San Jose, CA, USA).

### 2.4. Assay of hP2Y_14_R Binding

The affinity of three P2Y_6_R antagonists was tested at the hP2Y_14_R using whole-cell binding of a specific fluorescent antagonist MRS4174 [31] in CHO cells stably overexpressing the hP2Y_14_R (CHO-hP2Y_14_R). Briefly, 1 × 10^4^ cells were seeded on a 96-well plate for 24 h prior to the assay and incubated at 37 °C and 5% CO_2_ in Dulbecco’s modified Eagle’s medium/nutrient mixture F-12 (DMEM/F12) supplemented with 10% fetal bovine serum, 100 units/mL penicillin, 100 μg/mL streptomycin, and 0.5 mg/mL selective antibiotic G418 sulfate. On the day of the assay, three unlabeled P2Y_6_R antagonists (**24**, **30**, and **37**) were diluted separately in the serum-free media with a 10-fold serial dilution (1 nM–100 μM). The cells were first incubated with unlabeled ligands for 30 min at 37 °C and 5% CO_2_ followed by another 30 min of incubation with 20 nM fluorescent antagonist (MRS 4174). After the incubation, the cells were washed three times with Dulbecco’s phosphate-buffered saline (1X DPBS) without Ca^2+^/Mg^2+^ to remove the residual unlabeled and labeled fluorescent ligands. The cells were detached using Cellstripper and resuspended in 1X DPBS for a flow cytometry analysis. The competitive binding assays were performed on a CytoFLEX flow cytometer (Beckman Coulter, CA, USA). 

### 2.5. Cell Culture and Cell Viability

The 1321N1 astrocytoma (hP2Y_6_R-overexpressing) and HeLa (non-transfected) cell lines were cultured in Dulbecco’s modified Eagle’s medium (DMEM, HyClone, Logan, UT, USA) supplemented with 5% heat-inactivated fetal bovine serum (FBS, HyClone, UT, USA), 100 U/mL penicillin, and 100 µg/mL streptomycin in 5% CO_2_ at 37 °C. The cell viability was determined using an MTT kit (Sigma, St. Louis, MO, USA) according to the manufacturer’s instructions. Briefly, 1 × 10^4^ cells were seeded in triplicate in 96-well plates. The hP2Y_6_R-astrocytoma and HeLa cells were treated with a P2Y_6_R agonist and antagonists at a range of concentrations and incubated for 12 h. The cell viability was measured at 490 nm using a SpectraMax-M5 Spectrometer (Molecular Devices, San Jose, CA, USA). 

### 2.6. Statistical Analysis

The pharmacological parameters were determined using the Prism 10.2.2 software (GraphPad, San Diego, CA, USA). The values are expressed as the mean ± standard error mean. The data were analyzed by analysis of variance (ANOVA) followed by post hoc analysis to check the statistical difference among the groups with *p* < 0.05 being considered significant.

## 3. Results

### Functional Antagonism

As reported in our previous studies [30,40], the mono-halo substitution of the core 3-nitro-2-(trifluoromethyl)-2*H*-chromene heterocycle in **5**–**11** indicates a preference for 8- and 6-position substitution, compared to the 5 and 7 positions (Table 1). A comparison of the triethylsilyl-alkynyl groups placed at the 5, 6, and 8 positions (**12**–**14**) is consistent with the substitution preference at the 8 and 6 positions, with sub-µM affinity in derivatives **13** and **14**. Furthermore, the presence of a *p*-substituted phenyl ring with various terminal groups in derivatives **15**–**18** indicated the relative freedom of substitution at the 6 position, suggesting that it is located in a region on the receptor with no strict steric constraints. Previously, both hydrophilic (e.g., **16** and **17**) and hydrophobic terminal groups are tolerated, although none were found to greatly enhance the affinity. 

**Figure 3:**
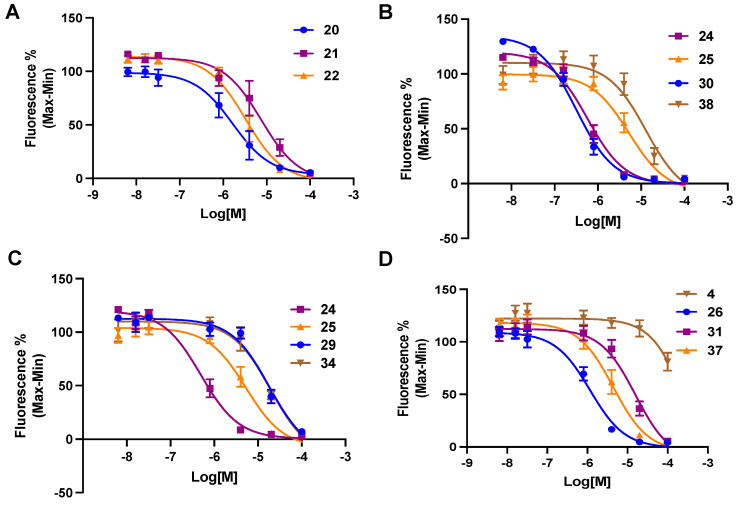
Representative curves (**A**–**D**), single experiments run in triplicate, for inhibition by selected 3-nitro-2-(trifluoromethyl)-2*H*-chromene derivatives, **20**–**22**, **24**–**26**, **29**–**31**, **34**, **37**, and **38**, and 1*H*-benzo[*d*]imidazole derivative **4** of calcium mobilization by agonist UDP (100 nM) in hP2Y_6_R-expressing astrocytoma cells. Mean IC_50_ ± SEM (*n* = 3) values are shown in Table 1.

Table 1 shows the structures and P2Y_6_R inhibitory affinity of newly synthesized (**19**–**41**) compounds, as well as their lipophilicity and aqueous solubility predictions. Sigmoidal inhibition curves for selected derivatives appear in Figure 3. There is no indication of P2Y_6_R agonist activity for any tested antagonists. The high affinity reported for compound **4**, in the reduction of IP_3_ production in response to 10 µM UDP in hP2Y_6_R-HEK cells [33], was not confirmed in our assay (Ca^2+^ mobilization in response to 100 nM UDP in hP2Y_6_R-1321N1 cells). Compound **4** appeared to be only a weak hP2Y_6_R antagonist (18.9 ± 8.5% inhibition at 100 µM).

Given the unknown receptor binding site location for this series, we expanded the SAR by probing the distal changes while maintaining the CF_3_ and NO_2_ groups as constant. Instead of a rigid phenyl ring, we introduced flexible alkyl or oxyalkyl (PEG) groups on the anchoring ethynyl moiety. Varying both the length and the terminal functionality of these groups had characteristic effects on the hP2Y_6_R affinity. The 6 position (**19**–**35**) was more extensively modified than the 8 position (**36**–**41**). A progression from a chain length of 3 to 8 methylenes extending the ethynyl moiety was examined, and the affinity values are also shown graphically, comparing the terminal primary amines and terminal Boc-amines (Figure S2, Supplementary Materials). The pattern of Boc-amino at the chain terminus (6 position) being more potent than unblocked amino was consistent for different chain lengths (except for 8 methylenes). The progression of affinity with increasing chain length was not smooth (7 > 4 > 3 > 6 > 8 methylenes), which probably indicates specific interactions with groups on the receptor rather than tethering away from the receptor. Thus, there was no clear definition of the environment around the appended chains or evidence that they reach polar head groups of the phospholipid bilayer [38], as was our original hypothesis. 

A terminal Br group in **19** and **36** was associated with moderate µM affinity. At the 6 position, the Boc-amino derivatives tended to have greater antagonist potency than the corresponding amino derivatives. The presence of the Boc-amino-protecting group in the 6-position-derivatized **30** enhanced the affinity by 17-fold compared to the primary amino derivative **29**. However, removing the Boc-amino-protecting group in the 8-position-derivatized **40** did not affect the affinity, which was weak in both cases (IC_50_ 20–30 µM). Curiously, the extension of Boc derivative **30** by one single carbon in **33** reduced it by 160-fold. At both the 6 and 8 positions, the introduction of a hydrophilic PEG chain terminating in a carboxylic acid greatly reduced the affinity, which is consistent with a mainly hydrophobic environment. 

Prediction of the physicochemical and pharmacokinetic properties was performed using the Stardrop Software (Version 7.4.0, Table 1 and Table S1, Supplementary Materials) [41]. Among the reference compounds, nonpolar compounds **2** and **5**–**14** were predicted to cross the BBB. Among the newly synthesized analogues, only the two ω-bromo-alkyl analogues **19** and **36**, both lacking H-bond donors, were predicted to cross the blood–brain barrier (BBB), and all except the carboxylate analogues **35** and **41** were predicted to have intestinal bioavailability. Most other compounds had only one H-bond donor, which contributed to *human* intestinal permeability and high flexibility. However, the potent compound **30** had low predicted aqueous solubility (0.86 mg/mL), while the ω-aminoalkyl analogues had a predicted aqueous solubility of ~100 mg/mL and pKa of 8.7–9. Although a nitro group is unusual in pharmaceuticals, there are examples of approved drugs containing this functionality [41]. 

**Figure 4:**
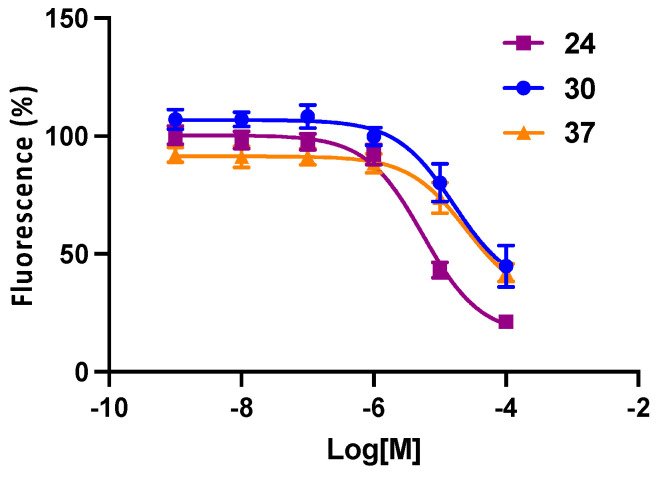
Affinity of selected P2Y_6_R antagonists in a fluorescent assay of hP2Y_14_R binding; screening for CHO-hP2Y_14_ (flow cytometer, competitive binding) by the method described [31]. Mean IC_50_ ± SEM (*n* = 3) values (µM) were as follows: **24**, 5.6 ± 0.7; **30**, 21.4 ± 5.8; and **37**, 31.2 ± 12.2.

The original lead compound **2a** was shown to be P2Y_6_R-selective compared to all the other P2YRs [29]. The antagonistic affinity at one other P2YR, i.e., hP2Y_14_R, was determined here for the selected compounds (Figure 4) using a previously reported fluorescent binding assay in whole cells [31]. The P2Y_14_R affinity was generally weak. Broader off-target binding interactions at 45 different membrane-bound proteins were assayed by the NIMH Psychoactive Drug Screening Program (PDSP) [42] for acylamino derivatives **20**, **24**, **26**, **30**, **31,** and **37** (Table 2 and Table S2). Curiously, while the highest affinity P2Y_6_R antagonist **30** displayed seven off-target interactions, including relatively potent interactions at the α_2A_- and α_2B_-adrenergic receptors, the corresponding pivaloyl derivative **31** (only one O atom different) had only two weak interactions (K_i_, µM): α_2A_, 3.5; σ_2_, 3.3. In contrast, 6,8-difluoro analogue **2b** had no off-target interactions. 

Although previous members of this chemical series were shown to be inactive in hP2Y_14_R binding, including **2a**, **7**, **8**, **15**, and **16** [29,43], three Boc-containing antagonists, **24**, **30,** and **37**, displayed < µM IC_50_ values (Figure 4). The most potent hP2Y_6_R antagonists in this series, **24** and **30**, were 10- and 132-fold selective, respectively, compared to the hP2Y_14_R. The inhibition at most other P2YRs by **2a**, **13**, **14**, and **17** was shown to be minor at high µM concentrations [29,43].

**Figure 5:**
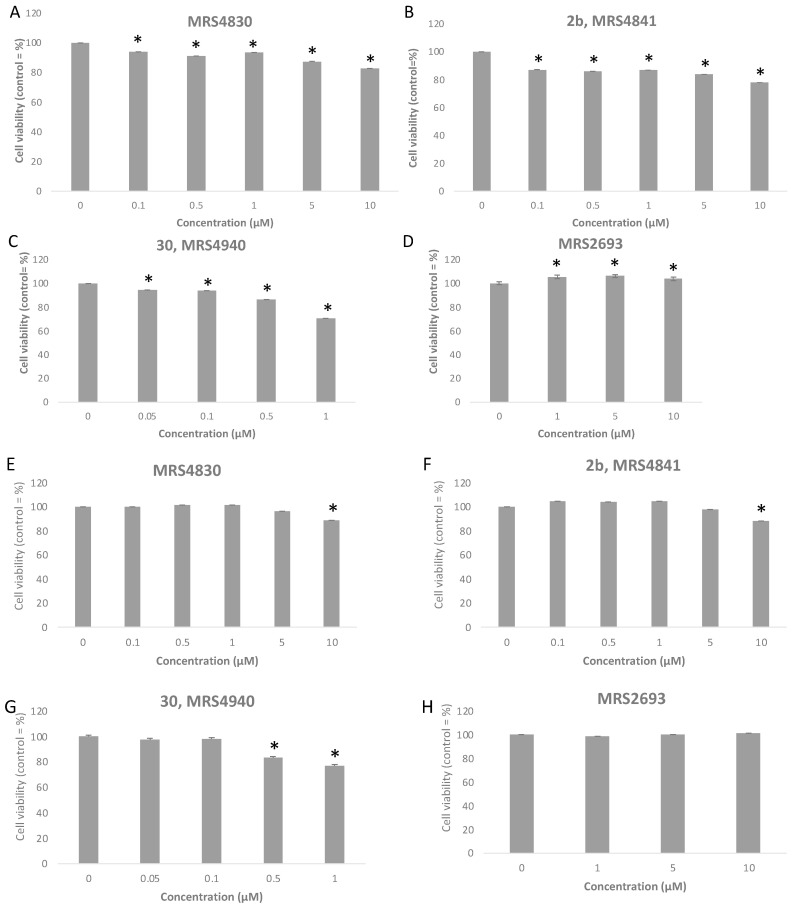
Cell viability of two cell lines (HeLa, (**A**–**D**); P2Y_6_R-astrocytoma, (**E**–**H**)) treated with antagonists MRS4830 (6-fluoro-3-nitro-2-(trifluoromethyl)-2*H*-chromene), **2b**, and **30** and with agonist MRS2693 at a range of concentrations up to 10 µM, following exposure for 12 h. Viability was determined using the MTT assay [44], showing mean ± SEM as a percentage of control. * *p* < 0.05. Percent data are in Supplementary Materials.

Potent P2Y_6_R agonist 5-iodouridine-5′-*O*-diphosphate (MRS2693, EC_50_ 15 nM in PLC activation) and three antagonists were tested for effects on cell viability using a 3-(4,5-dimethylthiazol-2-yl)-2,5-diphenyl-tetrazolium bromide (MTT) assay [44] in two cell lines, hP2Y_6_R-1321N1 *human* astrocytoma cells and HeLa cells (Figure 5, Table S3). The minor effects, following 12 h exposure, were slightly greater in the HeLa cells than in the astrocytoma cells. The agonist (≤10 µM) had no significant effect in the astrocytoma cells, but there was a modest increased (4–6%) viability at ≥1 µM in the HeLa cells. The antagonist MRS4841 **2b** [43] had only minor inhibition of viability at 10 µM (22% in the HeLa cells and 12% in the hP2Y_6_R-1321N1 cells). However, MRS4940 **30** at 1 µM inhibited viability by 29% in the HeLa cells and 23% in the hP2Y_6_R-1321N1 cells. The minor cell viability inhibition by **2b** and **30** at concentrations higher than their receptor affinity appeared to be dose-dependent, but there was no indication of P2Y_6_R-dependent effects on proliferation. Thus, we expect that this series of antagonists will be useful as pharmacological tool compounds. 

## 4. Discussion

In conclusion, we followed our previous results that the 2*H*-chromenes could be derivatized most effectively, with respect to receptor affinity, at the 6 and 8 positions. Further SAR exploration of 2*H*-chromene derivatives as P2Y_6_R antagonists has led to the identification of potent analogues containing an extended alkyl chain terminating in various functional groups, either charged or protected with a Boc-amino group. Curiously, the Boc-protected analogues were consistently more potent than the free amines, suggesting a hydrophobic environment around the extended chains, unlike earlier studies of GPCR-functionalized congeners. Consistently, carboxylates on extended polar PEG chains greatly reduced the affinity. The affinity was not dependent on the methylene chain length. The highest affinity was observed for the 6-(Boc-aminoheptyl) analogue MRS4940 **30** (IC_50_ 162 nM) and 4-(Boc-aminobutyl) analogue MRS4944 **24** (IC_50_ 568 nM). Compound **30** was 132-fold selective compared to the P2Y_14_R. When the identical chain found in **30** was placed at the 8 position in **37**, the affinity was 31-fold lower. However, **30** also slightly inhibited the cell viability at 2.5 µM (15-fold higher than the IC_50_) and displayed off-target interactions with two adrenergic receptors. The µM (or in some cases with greater affinity) off-target inhibition of binding at various receptors by multiple analogues, mainly α-adrenergic and dopamine receptors, is also a limitation of the use of these compounds. Multiple compounds at 10 µM displayed a small reduction in the cell viability in two cancer cell lines. 

## 5. Conclusions

We have improved the affinity of previous 3-nitro-2-(trifluoromethyl)-2*H*-chromene derivatives as P2Y_6_R antagonists. The addition of a favored 6-ethynyl-linked chain indicated a hydrophobic receptor environment surrounding the pharmacophore. Although the P2Y_6_R affinity depended on the chain length, attachment point, and terminal functionality of the added chain, there was not a smooth progression of affinity with increased length. Nevertheless, some patterns were evident, such as the high affinity of Boc-protected amines. Thus, despite the limitations of this 2*H*-chromene series, and given the difficulty of discovery of new chemotypes as antagonists, some of these analogues may now be evaluated further to probe the applicability of P2Y_6_R antagonists for potential treatment of neurodegeneration, cancer, and inflammatory and other conditions.

## Figures and Tables

**Table 1:** The inhibition of UDP (100 nM)-induced Ca^2+^ mobilization in hP2Y_6_R stably transfected 1321N1 *human* astrocytoma cells. ^a^ The structure of compound **4** appears in Figure 1.

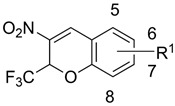 **2**, **5–41**			
**Compound**	**R^1^ =**	**cLogD (cLogS, pH7.4)**	**IC_50_, hP2Y_6_R (µM, Mean ± SEM) ^c^** **or *% inhib.***
Halo substitution (reported compounds)			
**2a** ^b,c,d^	H	3.32 (2.10)	2.91 ± 1.21
**2b** ^c,d^	6,8-diF	3.52 (1.11)	2.99 ± 0.56
**4**	-	5.22 (0.937)	>100
**5** ^c^	5-Cl	3.88 (1.92)	15.7 ± 4.6
**6** ^c^	6-Cl	3.94 (2.10)	1.79 ± 0.43
**7** ^c^	7-Cl	3.88 (1.92)	7.98 ± 0.42
**8** ^c^	8-Cl	3.88 (1.92)	3.69 ± 0.47
**9** ^c,d^	6-Br	4.17 (1.71)	3.49 ± 1.54
**10** ^c,d^	6-I	4.52 (1.63)	4.00 ± 1.59
**11** ^c^	8-I	4.40 (1.44)	4.09 ± 1.04
Alkyne substitution (reported compounds)			
**12** ^c^	5-C≡C-Si(CH_2_CH_3_)_3_	6.47 (0.369)	4.77 ± 2.73
**13**	6-C≡C-Si(CH_2_CH_3_)_3_	6.47 (0.369)	0.604 ± 0.239
**14** ^c,d^	8-C≡C-Si(CH_2_CH_3_)_3_	6.47 (0.369)	0.461 ± 0.270
**15** ^c^	6-C≡C-Ph-p-OH	5.33 (0.670)	5.36 ± 1.28
**16** ^c,d^	6-C≡C-Ph-p-CH_2_OH	4.70 (0.806)	2.00 ± 0.30
**17** ^b,c,d^	6-C≡C-Ph-p-CO_2_H	1.34 (2.50)	1.09 ± 0.42
**18** ^c^	6-C≡C-Ph-p-CO_2_CH_3_	5.59 (0.0912)	3.97 ± 1.58
Newly synthesized 6-substituted compounds			
**19**	6-C≡C-(CH_2_)_8_Br	6.06 (−0.0142)	14.0 ± 4.7
**20** ^d^	6-C≡C-(CH_2_)_2_-NH-Boc	4.60 (0.679)	0.587 ± 0.087
**21**	6-C≡C-(CH_2_)_2_-NH_2_	1.09 (2.38)	22.6 ± 9.3
**22**	6-C≡C-(CH_2_)_3_-NH-Boc	4.89 (0.507)	1.87 ± 0.11
**23**	6-C≡C-(CH_2_)_3_-NH_2_	1.28 (2.30)	9.98± 1.37
**24** ^d^	6-C≡C-(CH_2_)_4_-NH-Boc	5.16 (0.344)	0.568 ± 0.163
**25**	6-C≡C-(CH_2_)_4_-NH_2_	1.50 (2.18)	5.39 ± 1.49
**26**	6-C≡C-(CH_2_)_5_-NH-Boc	5.45 (0.163)	1.19± 0.25
**27**	6-C≡C-(CH_2_)_5_-NH_2_	1.68 (2.04)	12.4± 1.3
**28**	6-C≡C-(CH_2_)_6_-NH-Boc	5.66 (0.0289)	10.5 ± 6.5
**29**	6-C≡C-(CH_2_)_6_-NH_2_	1.83 (1.90)	20.0 ± 5.1
**30** ^d^	6-C≡C-(CH_2_)_7_-NH-Boc	5.84 (−0.064)	0.162 ± 0.013
**31**	6-C≡C-(CH_2_)_7_-NH-pivaloyl	5.69 (0.0417)	17.3 ± 5.2
**32**	6-C≡C-(CH_2_)_7_-NH_2_	1.95 (1.77)	6.37 ± 1.82
**33**	6-C≡C-(CH_2_)_8_-NH-Boc	6.26 (−0.302)	45.4 ± 17.7
**34**	6-C≡C-(CH_2_)_8_-NH_2_	2.06 (1.64)	20.0 ± 5.2
**35**	6-C≡C-CH_2_(OCH_2_CH_2_)_4_-CO_2_H	0.943 (2.53)	63.3 ± 30.0
Newly synthesized 8-substituted compounds			
**36**	8-C≡C-(CH_2_)_8_Br	6.06 (−0.014)	4.79 ± 1.45
**37**	8-C≡C-(CH_2_)_7_-NH-Boc	5.84 (−0.064)	4.96 ± 1.96
**38**	8-C≡C-(CH_2_)_7_-NH_2_	1.97 (1.69)	13.0 ± 3.9
**39**	8-C≡C-(CH_2_)_8_-NH-Boc	6.01 (−0.123)	23.8 ± 6.8
**40**	8-C≡C-(CH_2_)_8_-NH_2_	2.08 (1.56)	34.8 ± 16.3
**41**	8-C≡C-CH_2_(OCH_2_CH_2_)_4_-CO_2_H	0.948 (2.42)	>50

^a^ Ca^2+^ assays performed using hP2Y_6_R-expressing 1321N1 astrocytoma cells (gift of T. K. Harden, Univ. of North Carolina, Chapel Hill, NC) [12,30,39]. UDP (100 nM) was used to activate the P2Y_6_R. A total of 3–4 independent triplicate determinations. cLogD was predicted using StarDrop software, v. 7.6.1 (full data in Supporting Information). ^b^ Unsubstituted reference compound from Ito et al., 2017 [29]; and Jung et al., 2021 [30]. IC_50_ values shown were determined here. ^c^ IC_50_ values shown were determined in Jung et al., 2022 [40]. ^d^ **2a**, TIM-38; **2b**, MRS4841; **10**, MRS4666; **14**, MRS4853; **16**, MRS4816; **17**, MRS4656; **20**, MRS4948; **24**, MRS4944; and **30**, MRS4940.

**Table 2:** Off-target interactions of selected antagonists, determined in binding assays at 45 target sites by the PDSP (mean, *n* = 2) [42].

Compd	Structure	Receptor, K_i_ in µM ^c^
**2b**	6,8-di-F	none
**13** ^a^	6-C≡C-Si(CH_2_CH_3_)_3_	α_2A_, 2.0
**16** ^a^	6-C≡C-Ph-p-CH_2_OH	D_5_, 0.60
**17** ^a^	6-C≡C-Ph-p-CO_2_H	D_1_, 1.1; D_5_, 2.1; µ-opioid, 4.2; α_2A_, 1.8; α_2B_, 1.0; α_2C_, 2.6
**20** ^b^	6-C≡C-(CH_2_)_2_-NH-Boc	α_2A_, 1.6; σ_1_, 6.5; σ_2_, 3.1; D_5_, 4.4
**24** ^b^	6-C≡C-(CH_2_)_4_-NH-Boc	D_1_, 1.9; D_5_, 2.4; H_1_, 1.0; α_2A_, 0.16; α_2B_, 0.46; 5-HT_1D_, 3.2; σ_2_, 1.5
**26** ^b^	6-C≡C-(CH_2_)_5_-NH-Boc	α_2A_, 0.75; α_2B_, 1.7; σ_2_, 3.6; 5-HT_1D_, 4.8
**30** ^b^	6-C≡C-(CH_2_)_7_-NH-Boc	D_4_, 3.1; D_5_, 0.78; H_1_, 3.9; H_2_, 0.50; κ-opioid, 2.2; α_2A_, 0.086; α_2B_, 0.17
**31** ^b^	6-C≡C-(CH_2_)_7_-NH-pivaloyl	α_2A_, 3.5; σ_2_, 3.3
**37** ^b^	8-C≡C-(CH_2_)_7_-NH-Boc	α_2A_, 5.2; α_2B_, 4.8; σ_2_, 4.1; D_5_, 4.2; H_1_, 7.3; 5-HT_1D_, 3.5

^a^ reported in Jung et al., 2021 and 2022 [30,40]. ^b^ **2b**, MRS4841; **20**, MRS4948; **24**, MRS4944; **26**, MRS4952; **30**, MRS4940; **31**, MRS4955; and **37**, MRS4956. ^c^ If no value is given, the affinity at all other receptors, channels, and transporters examined, as shown in the Supplementary Materials file, are >10 µM.

## Data Availability

The data are available from the corresponding author upon reasonable request.

## Supplementary Materials

The following supporting information can be downloaded at https://www.mdpi.com/article/10.3390/cells13161366/s1, Additional synthesis of P2Y_6_ antagonists, pharmacological procedures, and results prediction using StarDrop software, version 7.6.1. Figure S1: Synthetic methods for non-chromene derivative **4**; Figure S2: Dependence of affinity on chain length and terminal group; Table S1: StarDrop (calculation of ADMET properties); Table S2: PDSP off-target screening; Table S3: Cell viability data.
