# Supplementary material for: Functionalized Congeners of 2H-Chromene P2Y6 Receptor Antagonists
Source: Cells. 2024 Aug 16;13(16):1366. doi: 10.3390/cells13161366 (PMC11352859; doi:10.3390/cells13161366)
Supplement: Supplementary file 1 [file cells-13-01366-s001.zip › cells-3128943-supplementary.pdf]

Supporting information

Functionalized Congeners of 2*H*-Chromene P2Y$_6$ Receptor Antagonists

Paola Oliva [†], Asmita Pramanik [†], Young-Hwan Jung, Sarah A. Lewicki, Jamie M. Mwendwa, Jong Hwan Park and Kenneth A. Jacobson *

Molecular Recognition Section, Laboratory of Bioorganic Chemistry, National Institute of Diabetes and Digestive and Kidney Diseases, National Institutes of Health, Bethesda, MD 20892, USA; paola.oliva@nih.gov (P.O.); asmita.pramanik@nih.gov (A.P.); jyh0401@gmail.com (Y.-H.J.); sarahlewicki2021@gmail.com (S.A.L.); jamiemikali@gmail.com (J.M.M.); jonghwan.park@nih.gov (J.H.P.)

* Correspondence: kennethj@niddk.nih.gov; Tel.: +301-496-9024; Fax: +301-480-8422

† These authors contributed equally to this work.

Contents page(s)

Synthetic methods for chromenes S2 – S7
Synthetic methods for non-chromene derivative **4** (**Figure S1**). S8 – S10
Spectra (NMR, mass) and HPLC purity of synthesized compounds S11 – S52
Figure S2. Dependence of affinity on chain length and terminal group. S53
Table S1. StarDrop (calculation of ADMET properties). S54 – S56
Table S2. PDSP off-target screening. S57
Table S3. Cell viability data. S58
References S59

Chemical Synthesis:

Refer to the synthetic route shown in Figure 2 and procedures in the main text. The synthetic methods are similar to those in Jung et al., 2022 [1].

6-(10-Bromodec-1-yn-1-yl)-3-nitro-2-(trifluoromethyl)-2H-chromene (**19**). The compound was synthesized following the same procedure for compound **36**, using 10-Br-1-decyne as alkyne. The residue was purified by silica gel column chromatography (hexane:ethyl acetate = 95:05) to afford the compound **19** (35% yield) as a yellow solid; ^{1}H NMR (400 MHz, $CDCl_3$) δ 8.06 (s, 1H), 7.45 (dd, *J* = 8.5, 2.0 Hz, 1H), 7.39 (d, *J* = 2.0 Hz, 1H), 6.99 (d, *J* = 8.5 Hz, 1H), 6.08 (q, *J* = 6.2 Hz, 1H), 3.41 (t, *J* = 6.8 Hz, 2H), 2.39 (t, *J* = 7.0 Hz, 2H), 1.93 – 1.79 (m, 2H), 1.67 – 1.52 (m, 4H), 1.39 – 1.45 (m, 6H).

tert-Butyl (4-(3-nitro-2-(trifluoromethyl)-2H-chromen-6-yl)but-3-yn-1-yl)carbamate (**20**). The compound was synthesized following the same procedure for compound **36**, using *tert*-butyl-but-3-yn-1-yl carbamate as alkyne. The residue was purified by silica gel column chromatography (hexane:ethyl acetate = 95:05) to afford compound **20** (67% yield) as a yellow solid; ^{1}H NMR (400 MHz, methanol-d_4) δ 8.35 (s, 1H), 7.61 (d, *J* = 2.0 Hz, 1H), 7.58 – 7.50 (m, 1H), 7.08 (d, *J* = 8.5 Hz, 1H), 6.40 (q, *J* = 6.5 Hz, 1H), 3.31 (t, *J* = 6.9 Hz, 1H), 3.21 (t, *J* = 6.8 Hz, 1H), 2.61 (t, *J* = 6.9 Hz, 1H), 2.45 (t, *J* = 6.8 Hz, 1H), 1.48 (s, 9H).

4-(3-Nitro-2-(trifluoromethyl)-2H-chromen-6-yl)but-3-yn-1-amine triethylammonium salt (**21**). To a solution of compound **20** (0.014 mmol), in DCM (3 mL), TFA (15 μL) were added at room temperature. The reaction mixture was stirred at room temperature for 3 h. This reaction mixture was diluted with water (5 mL) and extracted with DCM (10 mL x 3). The combined organic extracts were dried over anhydrous Na_2SO_4, filtered through a pad of Celite and evaporated under reduced pressure. The residue was purified by silica gel column chromatography (DCM:MeOH = 90:10) to afford the compound **21** (92%) as a yellow solid; ^{1}H NMR (400 MHz, methanol-d_4) δ 8.38 (s, 1H), 7.68 (d, *J* = 2.0 Hz, 1H), 7.61 (dd, *J* = 8.5, 2.1 Hz, 1H), 7.12 (d, *J* = 8.5 Hz, 1H), 6.43 (q, *J* = 6.5 Hz, 1H), 3.25 (p, *J* = 7.2 Hz, 18H), 2.89 (t, *J* = 6.9 Hz, 2H), 1.36 (t, *J* = 7.3 Hz, 21H). MS (ESI, m/z) 313.1 $[M+H]^+$; ESI-HRMS calcd. m/z for $C_{14}H_{12}N_2O_3F_3$ 313.0800, found 313.0797 $[M+H]^+$.

tert-Butyl (5-(3-nitro-2-(trifluoromethyl)-2H-chromen-6-yl)pent-4-yn-1-yl)carbamate (**22**). The compound was synthesized following the same procedure for compound **36**, using *tert*-butyl-pent-4-yn-1-yl carbamate as alkyne. The residue was purified by silica gel column chromatography (hexane:ethyl acetate = 90:10) to afford compound **22** (22% yield) as a yellow solid; ^{1}H NMR (400 MHz, $CDCl_3$) δ 8.05 (s, 1H), 7.45 (dd, *J* = 8.5, 2.0 Hz, 1H), 7.40 (d, *J* = 2.0 Hz, 1H), 6.99 (d, *J* = 8.5 Hz, 1H), 6.08 (q, *J* = 6.2 Hz, 1H), 4.67 (s, 1H), 3.28 (q, *J* = 6.5 Hz, 2H), 2.46 (t, *J* = 7.0 Hz, 2H), 1.79 (p, *J* = 6.9 Hz, 2H), 1.45 (s, 9H).

5-(3-Nitro-2-(trifluoromethyl)-2H-chromen-6-yl)pent-4-yn-1-amine triethylammonium salt (**23**). The compound was synthesized following the same procedure for compound **21**. The residue was purified by silica gel column chromatography (DCM:MeOH = 90:10) to afford compound **23** (70% yield) as a yellow solid; ^{1}H NMR (400 MHz, methanol-*d*$_4$) δ 8.58 (s, 1H), 7.83 (d, *J* = 2.0 Hz, 1H), 7.75 (dd, *J* = 8.5, 2.1 Hz, 1H), 7.31 (d, *J* = 8.4 Hz, 1H), 6.63 (q, *J* = 6.5 Hz, 1H), 3.47 (q, *J* = 7.3 Hz, 7H), 3.40 – 3.32 (m, 2H), 2.84 (t, *J* = 6.9 Hz, 2H), 2.20 (dq, *J* = 9.0, 7.0 Hz, 2H), 1.57 (t, *J* = 7.3 Hz, 13H). MS (ESI, m/z) 327.1 $[M+H]^+$; ESI-HRMS calcd. m/z for $C_{15}H_{14}N_2O_3F_3$ 327.0957, found 327.0948 $[M+H]^+$.

tert-Butyl (6-(3-nitro-2-(trifluoromethyl)-2H-chromen-6-yl)hex-5-yn-1-yl)carbamate (**25**). The compound was synthesized following the same procedure for compound **21**. The residue was purified by silica gel column chromatography (DCM:MeOH = 90:10) to afford compound **25** (52% yield) as a yellow solid; ^{1}H NMR (400 MHz, methanol-*d*$_4$) δ 8.32 (s, 1H), 7.56 (d, *J* = 2.0 Hz, 1H), 7.48 (dd, *J* = 8.5, 2.1 Hz, 1H), 7.05 (d, *J* = 8.5 Hz, 1H), 6.37 (q, *J* = 6.5 Hz, 1H), 3.04 – 2.94 (m, 2H), 2.51 (t, *J* = 6.8 Hz, 2H), 1.92 – 1.77 (m, 2H), 1.77 – 1.61 (m, 2H). MS (ESI, m/z) 341.1 $[M+H]^+$; ESI-HRMS calcd. m/z for $C_{16}H_{16}N_2O_3F_3$ 341.1113, found 341.1109 $[M+H]^+$.

tert-Butyl (7-(3-nitro-2-(trifluoromethyl)-2H-chromen-6-yl)hept-6-yn-1-yl)carbamate (**26**). The compound was synthesized following the same procedure for compound **36**, using *tert*-butyl-hept-6-yn-1-ylcarbamate as alkyne. The residue was purified by silica gel column chromatography (hexane:ethyl acetate = 90:10) to afford compound **26** (49% yield) as a yellow solid; ^{1}H NMR (400 MHz, methanol-*d*$_4$) δ 8.57 (s, 1H), 7.79 (d, *J* = 2.1 Hz, 1H), 7.74 – 7.67 (m, 1H), 7.28 (d, *J* = 8.5

Hz, 1H), 6.60 (q, *J* = 6.5 Hz, 1H), 3.38 – 3.23 (m, 2H), 2.66 (td, *J* = 6.9, 3.8 Hz, 2H), 1.86 (p, *J* = 7.0 Hz, 2H), 1.77 (ddt, *J* = 11.4, 8.2, 3.8 Hz, 4H), 1.67 (s, 9H). MS (ESI, m/z) 477.1 $[M+Na]^+$; ESI-HRMS calcd. m/z for $C_{22}H_{25}N_2O_5F_3Na$ 477.1613, found 477.1619 $[M+Na]^+$.

7-(3-Nitro-2-(trifluoromethyl)-2H-chromen-6-yl)hept-6-yn-1-amine triethylammonium salt (**27**). The compound was synthesized following the same procedure for compound **21**. The residue was purified by silica gel column chromatography (DCM:MeOH = 90:10) to afford compound **27** (66% yield) as a yellow solid; ^{1}H NMR (400 MHz, methanol-d_4) δ 8.68 (s, 1H), 7.91 (d, *J* = 2.0 Hz, 1H), 7.83 (dd, *J* = 8.5, 2.1 Hz, 1H), 7.40 (d, *J* = 8.5 Hz, 1H), 6.72 (q, *J* = 6.5 Hz, 1H), 3.57 (q, *J* = 7.3 Hz, 10H), 3.31 (t, *J* = 7.6 Hz, 2H), 2.82 (t, *J* = 6.8 Hz, 2H), 2.17 – 1.87 (m, 6H), 1.67 (t, *J* = 7.3 Hz, 15H). MS (ESI, m/z) 355.1 $[M+H]^+$; ESI-HRMS calcd. m/z for $C_{17}H_{18}N_2O_3F_3$ 355.1270, found 355.1275 $[M+H]^+$.

tert-Butyl (8-(3-nitro-2-(trifluoromethyl)-2H-chromen-6-yl)oct-7-yn-1-yl)carbamate (**28**). The compound was synthesized following the same procedure for compound **36**, using N-Boc-oct-7-yn-1-amine as alkyne. The residue was purified by silica gel column chromatography (hexane:ethyl acetate = 90:10) to afford compound **28** (80% yield) as a yellow solid; ^{1}H NMR (400 MHz, $CDCl_3$) δ 8.06 (s, 1H), 7.44 (dd, *J* = 8.5, 2.0 Hz, 1H), 7.40 (d, *J* = 2.0 Hz, 1H), 6.98 (d, *J* = 8.4 Hz, 1H), 6.07 (q, *J* = 6.2 Hz, 1H), 4.52 (s, 1H), 3.12 (q, *J* = 6.7 Hz, 2H), 2.38 (t, *J* = 7.0 Hz, 2H), 1.65 – 1.53 (m, 2H), 1.52 – 1.45 (m, 4H), 1.43 (s, 9H), 1.35 (tt, *J* = 11.0, 4.2 Hz, 2H). MS (ESI, m/z) 491.2 $[M+Na]^+$; ESI-HRMS calcd. m/z for $C_{23}H_{27}N_2O_5F_3Na$ 491.1770, found 491.1779 $[M+Na]^+$.

8-(3-Nitro-2-(trifluoromethyl)-2H-chromen-6-yl)oct-7-yn-1-amine triethylammonium salt (**29**). The compound was synthesized following the same procedure for compound **21**. The residue was purified by silica gel column chromatography (DCM:MeOH = 90:10) to afford compound **29** (88% yield) as a yellow solid; ^{1}H NMR (400 MHz, methanol-d_4) δ 8.28 (s, 1H), 7.49 (d, *J* = 2.0 Hz, 1H), 7.42 (dd, *J* = 8.5, 2.1 Hz, 1H), 6.99 (d, *J* = 8.5 Hz, 1H), 6.31 (q, *J* = 6.5 Hz, 1H), 3.01 – 2.73 (m, 2H), 2.39 (t, *J* = 6.9 Hz, 2H), 1.71 – 1.20 (m, 8H). MS (ESI, m/z) 369.1 $[M+H]^+$; ESI-HRMS calcd. m/z for $C_{18}H_{20}N_2O_3F_3$ 369.1426, found 369.1422 $[M+H]^+$.

N-(9-(3-Nitro-2-(trifluoromethyl)-2H-chromen-6-yl)non-8-yn-1-yl)pivalamide (**31**). To a solution of compound **30** (2.5 mg, 0.0065 mmol), in DCM (1 mL), TEA (1.8 µL, 0.013 mmol) and pivaloyl chloride (0.96 µL, 0.0078 mmol) were added at room temperature. The reaction mixture was stirred at room temperature for 3 h. This reaction mixture was diluted with water (5 mL) and extracted with DCM (10 mL x 3). The residue was purified by silica gel column chromatography (DCM:MeOH = 90:10) to afford compound **31** (52% yield) as a yellow solid; ^{1}H NMR (400 MHz, methanol-d_4) δ 8.69 (s, 1H), 7.90 (d, *J* = 2.1 Hz, 1H), 7.82 (dd, *J* = 8.5, 2.1 Hz, 1H), 7.43 – 7.35 (m, 1H), 6.72 (q, *J* = 6.5 Hz, 0H), 4.46 (q, *J* = 7.1 Hz, 2H), 3.58 – 3.41 (m, 2H), 2.76 (t, *J* = 7.0 Hz, 2H), 2.37 (m, 2H), 1.90 (m, 4H), 1.60 (m, 2H), 1.52 (s, 9H). MS (ESI, m/z) 467.2 $[M+H]^+$; ESI-HRMS calcd. m/z for $C_{24}H_{29}N_2O_4F_3$ 467.2158, found 467.2164 $[M+H]^+$.

9-(3-Nitro-2-(trifluoromethyl)-2H-chromen-6-yl)non-8-yn-1-amine (**32**). The compound was synthesized following the same procedure for compound **21**. The residue was purified by silica gel column chromatography (DCM:MeOH = 90:10) to afford the compound **32** (24% yield) as a yellow solid; ^{1}H NMR (400 MHz, methanol-d_4) δ 8.32 (s, 1H), 7.54 (d, *J* = 2.1 Hz, 1H), 7.46 (dd, *J* = 8.5, 2.1 Hz, 1H), 7.04 (d, *J* = 8.5 Hz, 1H), 6.37 (q, *J* = 6.5 Hz, 1H), 4.86 (s, 2H), 3.02 (t, *J* = 7.2 Hz, 2H), 2.96 – 2.87 (m, 2H), 2.42 (t, *J* = 6.9 Hz, 2H), 1.72 – 1.55 (m, 2H), 1.43 (p, *J* = 3.7 Hz, 2H). MS (ESI, m/z) 383.1 $[M+H]^+$; ESI-HRMS calcd. m/z for $C_{19}H_{22}N_2O_3F_3$ 383.1583, found 383.1576 $[M+H]^+$.

tert-Butyl (10-(3-nitro-2-(trifluoromethyl)-2H-chromen-6-yl)dec-9-yn-1-yl)-l2-azanecarboxylate (**33**). The compound was synthesized following the same procedure for compound **36**, using N-Boc-9-decyn-1-amine as alkyne. The residue was purified by silica gel column chromatography (hexane:ethyl acetate = 95:05) to afford the compound **33** (35% yield) as a yellow solid; ^{1}H NMR (400 MHz, $CDCl_3$) δ 8.06 (s, 1H), 7.45 (dd, *J* = 8.5, 2.0 Hz, 1H), 7.39 (d, *J* = 2.0 Hz, 1H), 6.99 (d, *J* = 8.5 Hz, 1H), 6.08 (q, *J* = 6.2 Hz, 1H), 4.48 (s, 1H), 3.10 (s, 2H), 2.38 (t, *J* = 7.1 Hz, 2H), 1.59 (p, *J* = 7.0 Hz, 2H), 1.44 (s, 13H), 1.35 – 1.28 (m, 6H). MS (ESI, m/z) 519.2 $[M+Na]^+$; ESI-HRMS calcd. m/z for $C_{25}H_{31}N_2O_5F_3Na$ 519.2083, found 519.2076 $[M+Na]^+$.

10-(3-Nitro-2-(trifluoromethyl)-2H-chromen-6-yl)dec-9-yn-1-amine (**34**). The compound was synthesized following the same procedure for compound **21**. The residue was purified by silica gel

column chromatography (DCM:MeOH = 90:10) to afford the compound **34** (39% yield) as a yellow solid; MS (ESI, m/z) 397.2 $[M+H]^+$; ESI-HRMS calcd. m/z for $C_{20}H_{23}N_2O_3F_3$ 397.1739, found 397.1737 $[M+H]^+$.

16-(3-Nitro-2-(trifluoromethyl)-2*H*-chromen-6-yl)-4,7,10,13-tetraoxahexadec-15-ynoic acid (**35**). The compound was synthesized following the same procedure for compound **36**, using propargyl-PEG4-acid (BroadPharm, San Diego, CA) as reagent. The residue was purified by silica gel column chromatography (dichloromethane:methanol = 90:10) to afford compound **35** (62% yield) as a yellow solid; HPLC purity 96%; ^{1}H NMR (400 MHz, $CDCl_3$) δ 8.06 (s, 1H), 7.50 (d, *J* = 8.48 Hz, 1H), 7.46 (s, 1H), 7.02 (d, *J* = 8.52 Hz, 1H), 6.09 (q, *J* = 6.16 Hz, 1H), 3.78-3.69 (m, 7H), 3.67-3.64 (m, 9H), 2.62 (t, *J* = 6.16 Hz, 2H); ^{19}F NMR (376 MHz, $CDCl_3$) δ -77.77 (d, *J* = 6.09 Hz, 3F); MS (ESI, m/z) 526.1 $[M+Na]^+$; ESI-HRMS calcd. m/z for $C_{22}H_{24}NO_9F_3Na$ 526.1301, found 526.1307 $[M+Na]^+$.

tert-Butyl (9-(3-nitro-2-(trifluoromethyl)-2H-chromen-8-yl)non-8-yn-1-yl)carbamate (**37**). The compound was synthesized following the same procedure for compound **36**. The residue was purified by silica gel column chromatography (hexane:ethyl acetate = 90:10) to afford compound **37** (yield 88%) as a yellow solid; ^{1}H NMR (400 MHz, $CDCl_3$) δ 8.36 (s, 1H), 7.77 (dd, *J* = 7.8, 1.6 Hz, 1H), 7.59 – 7.51 (m, 1H), 7.32 (d, *J* = 7.7 Hz, 1H), 6.45 (q, *J* = 6.2 Hz, 1H), 4.80 (s, 1H), 2.74 (t, *J* = 7.0 Hz, 2H), 2.52 (t, *J* = 6.9 Hz, 2H), 1.84 – 1.72 (m, 11H), 1.70 – 1.47 (m, 8H).

9-(3-Nitro-2-(trifluoromethyl)-2H-chromen-8-yl)non-8-yn-1-amine (**38**). To a solution of *tert*-butyl (9-(3-nitro-2-(trifluoromethyl)-2H-chromen-8-yl)non-8-yn-1-yl)carbamate (**37**, 5 mg, 0.014 mmol), in DCM (3 mL), TFA (4 µL, 0.028 mmol) were added at room temperature. The reaction mixture was stirred at room temperature for 3 h. This reaction mixture was diluted with water (5 mL) and extracted with DCM (10 mL x 3). The combined organic extracts were dried over anhydrous Na_2SO_4, filtered through a pad of Celite and evaporated under reduced pressure. The residue was purified by silica gel column chromatography (DCM:MeOH = 90:10) to afford compound **38** (yield 85%) as a yellow solid; ^{1}H NMR (400 MHz, $CDCl_3$) δ 8.35 (s, 1H), 7.76 (dd, *J* = 7.8, 1.6 Hz, 1H), 7.59 – 7.50 (m, 1H), 7.30 (t, *J* = 7.7 Hz, 1H), 6.44 (q, *J* = 6.2 Hz, 1H), 3.20

(s, 2H), 2.73 (t, *J* = 7.0 Hz, 4H), 2.00 – 1.83 (m, 6H), 1.76 (q, *J* = 8.3, 7.8 Hz, 2H). MS (ESI, m/z) 383.2 $[M+H]^+$; ESI-HRMS calcd. m/z for $C_{19}H_{22}N_2O_3F_3$ 383.1583, found 383.1581 $[M+H]^+$.

tert-Butyl (10-(3-nitro-2-(trifluoromethyl)-2H-chromen-8-yl)dec-9-yn-1-yl)carbamate (**39**). The compound was synthesized following the same procedure for compound **36**. The residue was purified by silica gel column chromatography (hexane:ethyl acetate = 90:10) to afford the compound **39** (yield 42%) as a yellow solid; ^{1}H NMR (400 MHz, Chlorofzorm-*d*) δ 8.08 (s, 1H), 7.49 (dd, *J* = 7.8, 1.6 Hz, 1H), 7.27 (d, *J* = 6.3 Hz, 1H), 7.03 (t, *J* = 7.7 Hz, 1H), 6.17 (q, *J* = 6.2 Hz, 1H), 4.48 (s, 1H), 3.10 (s, 2H), 2.46 (t, *J* = 7.0 Hz, 2H), 1.66 – 1.57 (m, 2H), 1.44 (s, 13H), 1.36 – 1.28 (m, 6H). MS (ESI, m/z) 519.2 $[M+Na]^+$; ESI-HRMS calcd. m/z for $C_{25}H_{31}N_2O_5F_3Na$ 519.2083, found 519.2082 $[M+Na]^+$.

10-(3-Nitro-2-(trifluoromethyl)-2H-chromen-8-yl)dec-9-yn-1-amine (**40**). The compound was synthesized following the same procedure for compound **38**. The residue was purified by silica gel column chromatography (DCM:MeOH = 90:10) to afford the compound **40** (yield 78%) as a yellow solid; ^{1}H NMR (400 MHz, $CDCl_3$) δ 8.07 (s, 1H), 7.97 (s, 1H), 7.48 (dd, *J* = 7.8, 1.6 Hz, 1H), 7.02 (t, *J* = 7.7 Hz, 1H), 6.16 (q, *J* = 6.2 Hz, 1H), 2.45 (t, *J* = 6.9 Hz, 4H), 1.75 – 1.53 (m, 10H), 1.46 (m, 2H). MS (ESI, m/z) 397.2 $[M+H]^+$; ESI-HRMS calcd. m/z for $C_{20}H_{24}N_2O_3F_3$ 397.1739, found 397.1743 $[M+H]^+$.

16-(3-Nitro-2-(trifluoromethyl)-2H-chromen-8-yl)-4,7,10,13-tetraoxahexadec-15-ynoic acid (**41**). The compound was synthesized following the same procedure for compound **36**. The residue was purified by silica gel column chromatography (hexane:ethyl acetate = 90:10) to afford compound **41** (yield 82%) as a yellow solid; ^{1}H NMR (400 MHz, $CDCl_3$) δ 8.08 (s, 1H), 7.59 – 7.41 (m, 1H), 7.33 (dd, *J* = 7.6, 1.5 Hz, 1H), 7.06 (t, *J* = 7.7 Hz, 1H), 6.17 (q, *J* = 6.2 Hz, 1H), 4.48 (s, 2H), 3.84 – 3.69 (m, 6H), 3.66 (d, *J* = 11.3 Hz, 8H), 2.62 (t, *J* = 6.2 Hz, 2H). MS (ESI, m/z) 521.2 $[M+H]^+$; ESI-HRMS calcd. m/z for $C_{22}H_{28}N_2O_9F_3$ 521.1747, found 521.1755 $[M+H]^+$.

Figure S1. Synthesis of compound **4** (modified from Zhu et al., 2023).[a]

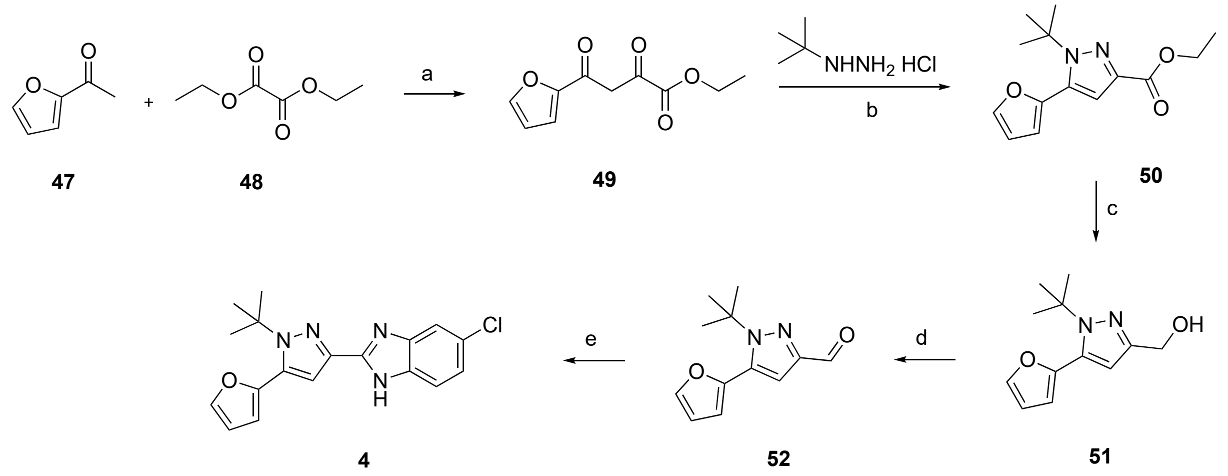

[a] Reagents and conditions: (a) NaH (60% w/w dispersion in mineral oil), diethyl oxalate, THF, 0 °C to reflux for 2 h; (b) *tert*-butylhydrazine, EtOH, r.t., overnight; (c) $LiAlH_4$, THF, 0 °C, under argon, 30 min, reflux, 2 h; (d) Dess-Martin reagent, DCM, 0 °C to r.t., 1 h; (e) 4-Cl-benzene-1,2-diamine, EtOH/H_2O (2:1, by volume), $NaHSO_3$, reflux, 1 h.

2-(1-(*tert*-Butyl)-5-(furan-2-yl)-1H-pyrazol-3-yl)-5-chloro-1H-benzo[d]imidazole (**4**). The aldehyde compound (0.128 mmol, 1 equiv) (**52**), *o*-phenylenediamine (0.192 mmol, 1.5 equiv), and sodium bisulfite (0.256 equiv, 2 equiv) were dissolved in a solution of ethanol/water = 2:1 and refluxed for 1 h. The reaction solution was extracted with ethyl acetate, dried over anhydrous sodium sulfate, spun dry under reduced pressure, and purified by fast column chromatography (hexane/ethyl acetate (85:15)) to obtain the pure target product **53**. Yield: 69%. ^{1}H NMR (400 MHz, DMSO-*d6*) δ 12.79 (bs, 1H), 7.92 (dd, J = 1.8 Hz, 1H), 7.56 (bs, 2H), 7.21 (s, 1H), 7.02 (s, 1H), 6.77 (dd, J = 3.2 Hz, 1H), 6.68 (dd, J = 3.3, 1.9 Hz, 1H), 1.55 (s, 9H). MS (ESI, m/z) 341.1 $[M+H]^+$; ESI-HRMS calcd. m/z for $C_{18}H_{18}N_4OCl$ 341.1169, found 341.1168 $[M+H]^+$. HPLC purity 97%.

Ethyl 4-(furan-2-yl)-2,4-dioxobutanoate (**49**). To a suspension of NaH (60% w/w dispersion in mineral oil) (176 mg, 3 equiv) in THF (3 mL) at 0ºC was added diethyl oxalate (741 µL, 3 equiv) (**48**). The resulting mix was stirred at RT for 15 min, then the (furan-2-yl)-ethan-1-one (200 mg, 1 equiv) (**47**) was added and the reaction was heated at reflux for 2 hours. The reaction was allowed to cool, the solvent was concentrated, added water, and adjusted to pH 4 wit 1.5 M HCl. The

resulting precipitate was filtered and washed to furnish pure compound **49**. Yield: 93%. The 1H spectrum of **49** was identical to that reported commercially (CAS:36983-35-4).

Ethyl 1-(tert-butyl)-5-(furan-2-yl)-1H-pyrazole-3-carboxylate (**50**). The *β*-dicarbonyl compound (0.48 mmol, 1 equiv) (**49**) was dissolved in ethanol, *tert*-butylhydrazine hydrochloride (0.48 mmol, 1 equiv) was added, and the mixture was stirred at room temperature overnight. When the reaction was completed, the crude product was extracted with water and ethyl acetate. The organic phase was dried with anhydrous sodium sulfate and spin dried under reduced pressure. The product was purified by silica gel column chromatography, eluting with hexane - ethyl acetate (100:0−85:15) to give pure compound **50**. Yield: 59%. The ^{1}H spectrum of **5** was identical to that reported commercially (CAS: 2098856-93-8).

(1-(*tert*-Butyl)-5-(furan-2-yl)-1H-pyrazol-3-yl)methanol (**51**). The ester compound (0.28 mmol, 1 equiv) (**50**) was dissolved in 0.6 mL of tetrahydrofuran and then added dropwise to a suspension of $LiAlH_4$ (22 mg, 2 equiv) in 2 mL of tetrahydrofuran at 0 °C and under argon protection. After holding at this temperature for 30 min, the reaction was heated to reflux for 2 h. The reaction was cooled to room temperature, and 5 mL of ethyl acetate was slowly added to the reaction. Sodium hydroxide (5 N) was slowly added dropwise to the reaction solution until a white precipitate appeared. The filter and filtrate were extracted with ethyl acetate and water. The organic layer was dried with magnesium sulfate, filtered, and spin dried under reduced pressure to give pure compound **51**. Yield: 89%. ^{1}H NMR (400 MHz, MeOD) *δ* 7.83 (dd, *J* = 1.9 Hz, 1H), 6.54 (dd, dd, *J* = 4ff.0 Hz, 1H) 6.60 (dd, *J* = 2.0 Hz, 1H), 6.37 (s, 1H), 4.57 (s, 2H), 1.49 (s, 9H).

1-(*tert*-Butyl)-5-(furan-2-yl)-1H-pyrazole-3-carbaldehyde (**52**). The alcohol compound (0.24 mmol, 1 equiv) (**51**) was dissolved in DCM, and the Dess-Martin reagent (0.29 mg, 1.2 equiv) was added under an ice bath. The solution was stirred for 1 h at room temperature, quenched with saturated sodium carbonate, and filtered with diatomaceous earth, and the filtrate was extracted with ethyl acetate. It was then dried with anhydrous sodium sulfate, spin dried under reduced pressure, and purified by silica gel column chromatography, eluting with hexane/ethyl acetate (90:10) to give pure compound **52**. Yield: 78%. ^{1}H NMR (400 MHz, MeOD) *δ* 9.87 (s, 1H), 7.71 (dd, *J* = 1.9 Hz, 1H), 6.86 (s, 1H), 6.61 (d, *J* = 3.3 Hz, 1H),6.40−6.53 (m, 1H), 1.56 (s, 9H).

Compound 37

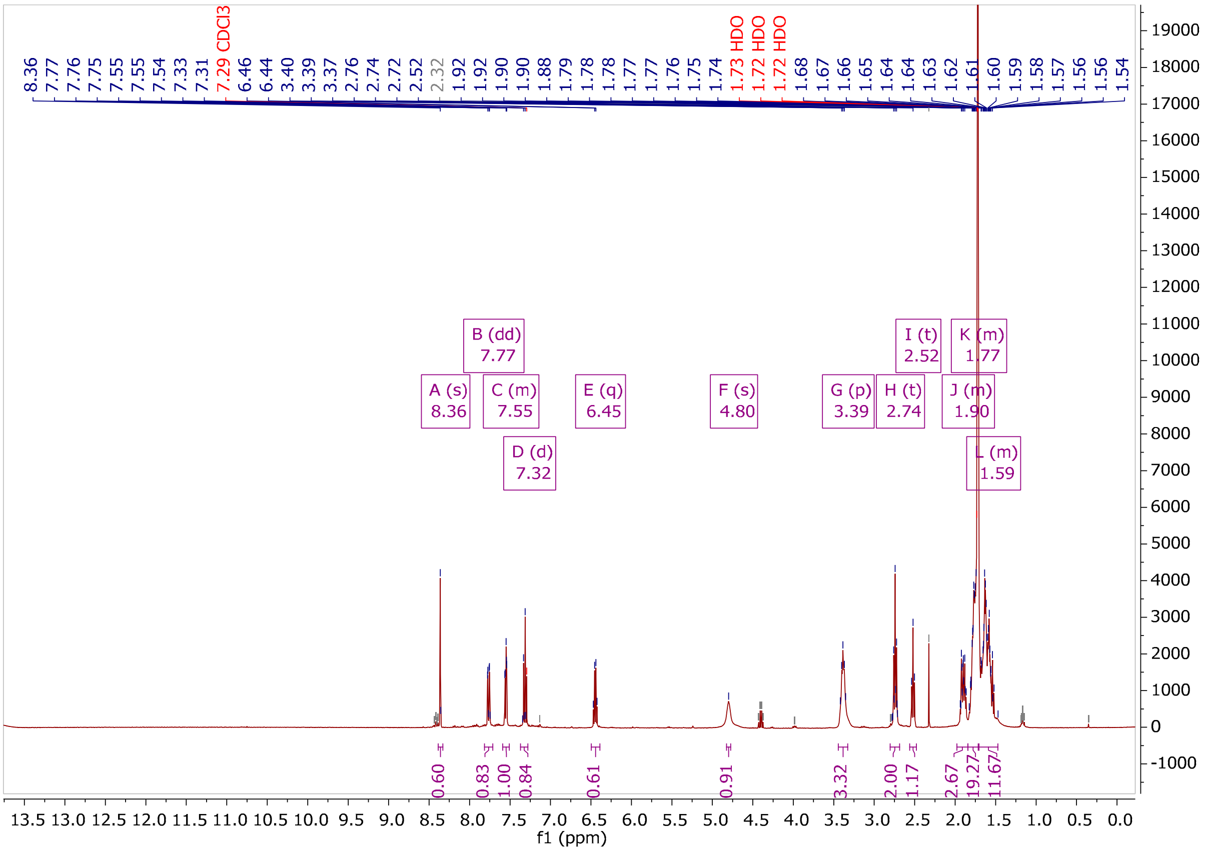

8.36
7.77
7.76
7.75
7.55
7.55
7.54
7.33
7.31
7.29 CDCl3
6.46
6.44
3.40
3.39
3.37
2.76
2.74
2.72
2.52
2.32
1.92
1.92
1.90
1.90
1.88
1.79
1.78
1.78
1.77
1.77
1.76
1.75
1.74
1.73 HDO
1.72 HDO
1.72 HDO
1.68
1.67
1.66
1.65
1.64
1.64
1.63
1.62
1.61
1.60
1.59
1.58
1.57
1.56
1.56
1.54
A (s) 8.36
B (dd) 7.77
C (m) 7.55
D (d) 7.32
E (q) 6.45
F (s) 4.80
G (p) 3.39
H (t) 2.74
I (t) 2.52
J (m) 1.90
K (m) 1.77
L (m) 1.59
0.60
0.83
1.00
0.84
0.61
0.91
3.32
2.00
1.17
2.67
19.27
11.67
f1 (ppm)

Compound 39

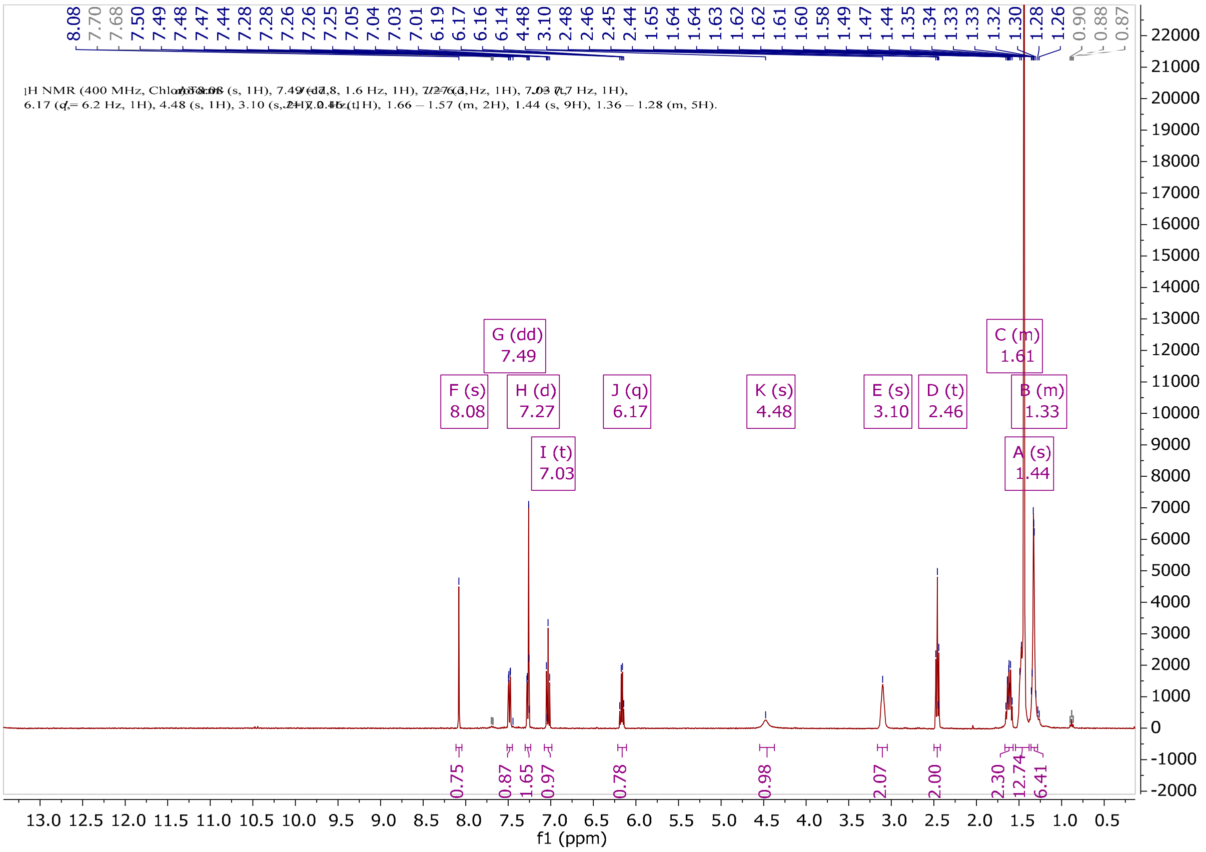
1H NMR (400 MHz, Chloroform-d) δ 8.08 (s, 1H), 7.49 (dd, J = 7.8, 1.6 Hz, 1H), 7.27 (d, J = 7.6 Hz, 1H), 7.03 (t, J = 7.7 Hz, 1H), 6.17 (q, J = 6.2 Hz, 1H), 4.48 (s, 1H), 3.10 (s, 2H), 2.46 (t, J = 7.0 Hz, 2H), 1.66 – 1.57 (m, 2H), 1.44 (s, 9H), 1.36 – 1.28 (m, 5H).
f1 (ppm)

Data File C:\Chem32\1\Data\PAOLA\Paola 2023-01-31 11-15-25\PO2120000005.D
Sample Name: PO212

```
=====================================================================
Acq. Operator   : SYSTEM                          Seq. Line :    1
Acq. Instrument : hplc                             Location :    1
Injection Date  : 1/31/2023 11:17:15 AM                 Inj :    1
                                                 Inj Volume : Inj prog
Method          : C:\Chem32\1\Data\PAOLA\Paola 2023-01-31 11-15-25\Paola_A40-90_B_20MIN.M (
                  Sequence Method)
Last changed    : 1/31/2023 11:15:25 AM by SYSTEM
Additional Info : Peak(s) manually integrated
```

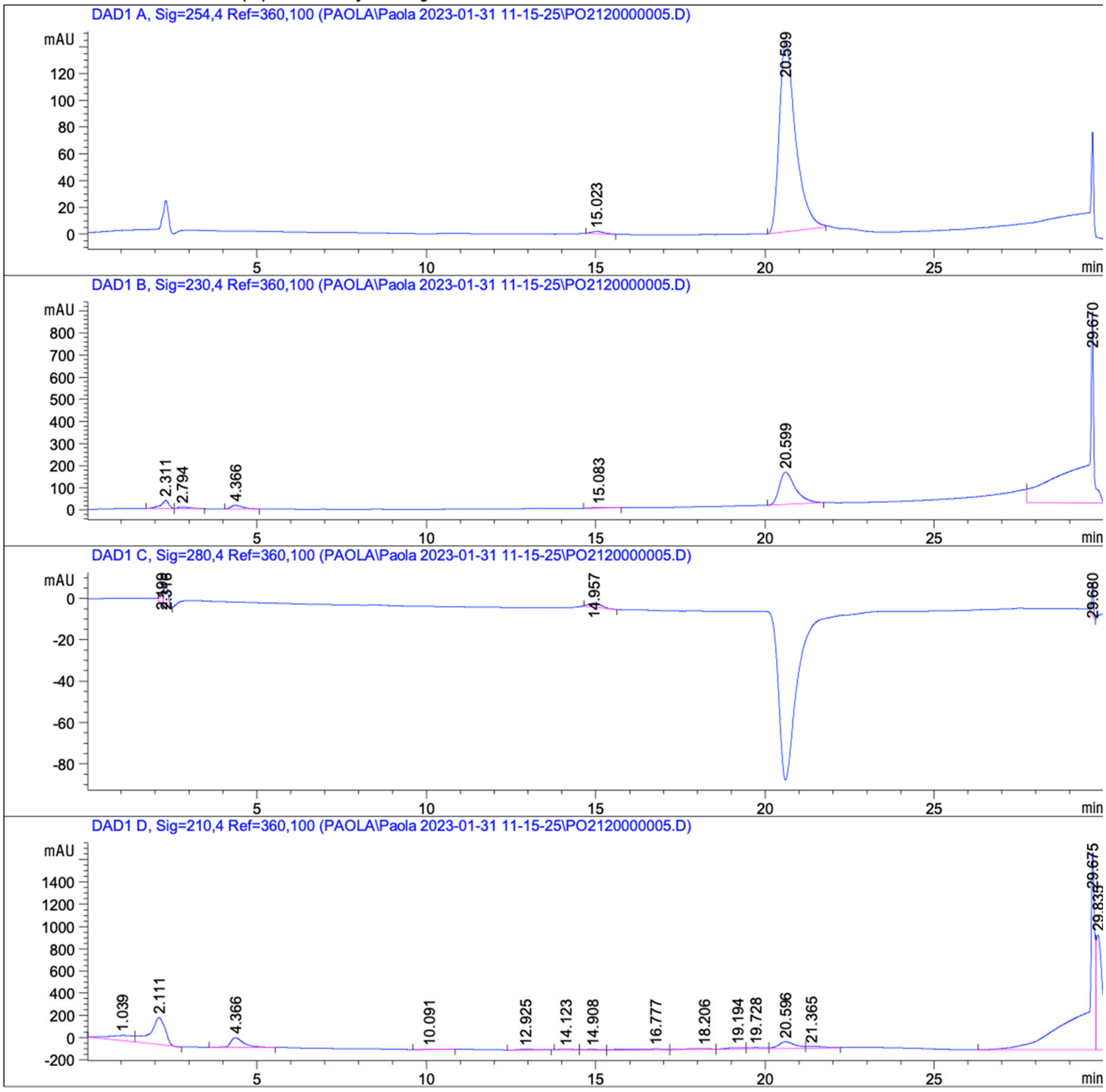

Data File C:\Chem32\1\Data\PAOLA\Paola 2023-01-31 11-15-25\PO2120000005.D
Sample Name: PO212

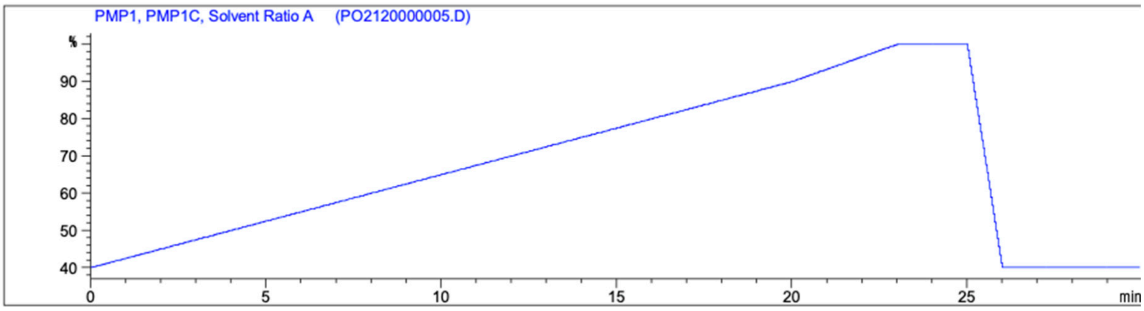

===

External Standard Report

===

Sorted By : Signal
Multiplier : 1.0000
Dilution : 1.0000
Sample Amount: : 1.00000 [wt%] (not used in calc.)
Do not use Multiplier & Dilution Factor with ISTDs

===

===

Area Percent Report

===

Sorted By : Signal
Multiplier : 1.0000
Dilution : 1.0000
Sample Amount: : 1.00000 [wt%] (not used in calc.)
Do not use Multiplier & Dilution Factor with ISTDs

Signal 1: DAD1 A, Sig=254,4 Ref=360,100

Peak #	RetTime [min]	Type	Width [min]	Area [mAU*s]	Height [mAU]	Area %
1	15.023	BB	0.3631	38.95404	1.55038	0.8071
2	20.599	BB	0.5019	4787.24707	142.53172	99.1929

Totals : 4826.20111 144.08210

Signal 2: DAD1 B, Sig=230,4 Ref=360,100

Peak #	RetTime [min]	Type	Width [min]	Area [mAU*s]	Height [mAU]	Area %
1	2.311	BV	0.2283	620.87079	37.66441	2.5886
2	2.794	VB	0.3657	200.93776	7.87371	0.8378
3	4.366	BB	0.3615	395.45749	16.50412	1.6488
4	15.083	BB	0.4235	91.21790	3.28963	0.3803
5	20.599	BB	0.4972	4872.87891	146.06252	20.3165
6	29.670	BBA	0.2560	1.78034e4	865.28943	74.2280

Compound 41

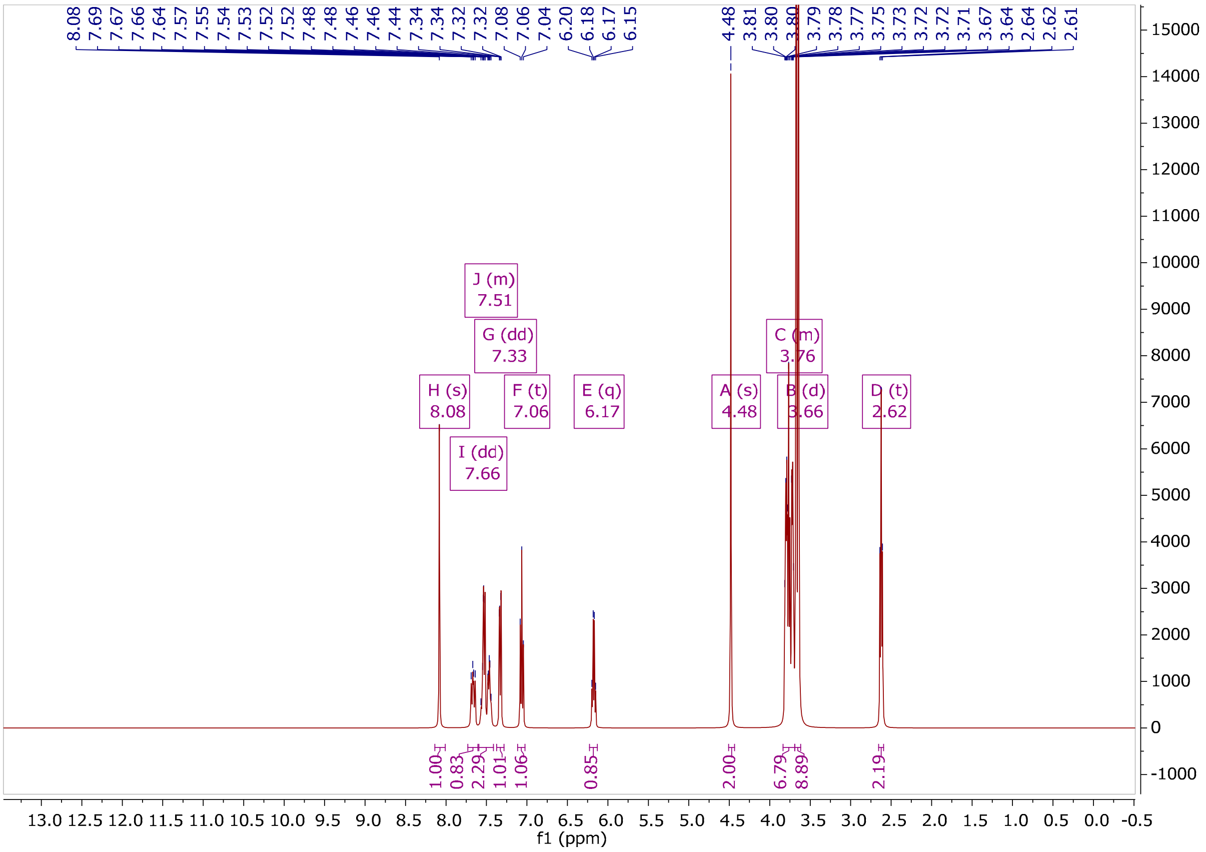

8.08 7.69 7.67 7.66 7.64 7.57 7.55 7.54 7.53 7.52 7.52 7.48 7.48 7.46 7.46 7.44 7.34 7.34 7.32 7.32 7.08 7.06 7.04 6.20 6.18 6.17 6.15
4.48 3.81 3.80 3.80 3.79 3.78 3.77 3.75 3.73 3.72 3.72 3.71 3.67 3.64 2.64 2.62 2.61
J (m) 7.51
G (dd) 7.33
H (s) 8.08
F (t) 7.06
E (q) 6.17
I (dd) 7.66
A (s) 4.48
C (m) 3.76
B (d) 3.66
D (t) 2.62
1.00 0.83 2.29 1.01 1.06 0.85 2.00 6.79 8.89 2.19
f1 (ppm)

Data File C:\Chem32\1\Data\PAOLA\Paola 2023-01-06 13-00-54\PO2090000002.D
Sample Name: PO209

===

Acq. Operator : SYSTEM Seq. Line : 1
Acq. Instrument : hplc Location : 1
Injection Date : 1/6/2023 1:02:49 PM Inj : 1
Inj Volume : 100.000 µl
Different Inj Volume from Sample Entry! Actual Inj Volume : 3.000 µl
Method : C:\Chem32\1\Data\PAOLA\Paola 2023-01-06 13-00-54\HBL_A05-95_B95-05_20MIN.M
(Sequence Method)
Last changed : 1/6/2023 1:00:55 PM by SYSTEM
Additional Info : Peak(s) manually integrated

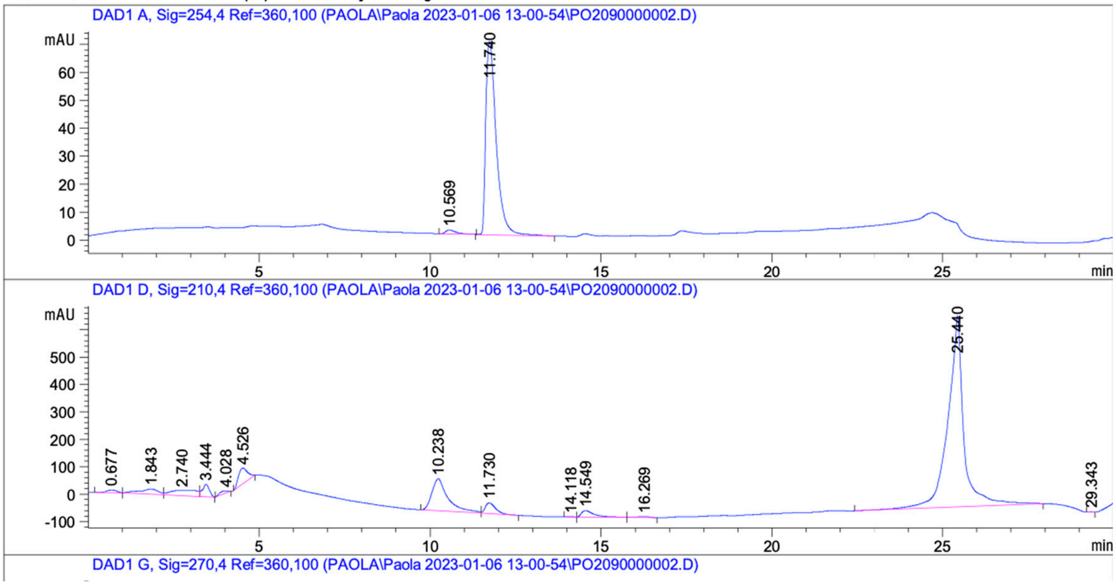

Data File C:\Chem32\1\Data\PAOLA\Paola 2023-01-06 13-00-54\PO2090000002.D
Sample Name: PO209

===

Area Percent Report

===

Sorted By : Signal
Multiplier : 1.0000
Dilution : 1.0000
Sample Amount: : 1.00000 [ng/ul] (not used in calc.)
Use Multiplier & Dilution Factor with ISTDs

Signal 1: DAD1 A, Sig=254,4 Ref=360,100

Peak #	RetTime [min]	Type	Width [min]	Area [mAU*s]	Height [mAU]	Area %
1	10.569	BB	0.3040	30.18341	1.39763	2.0091
2	11.740	BB	0.3286	1472.17786	69.08029	97.9909

Totals : 1502.36127 70.47792

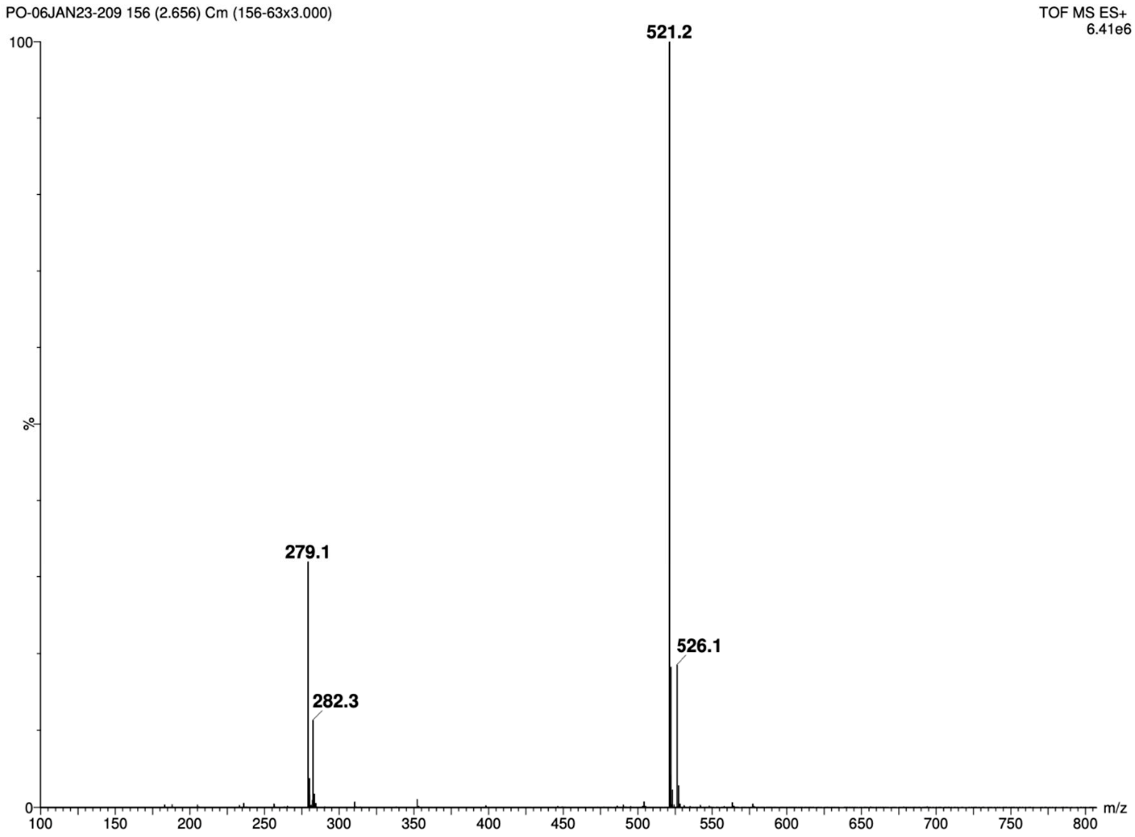
PO-06JAN23-209 156 (2.656) Cm (156-63x3.000)
TOF MS ES+
6.41e6
521.2
279.1
526.1
282.3
100
%
0
m/z
100
150
200
250
300
350
400
450
500
550
600
650
700
750
800

Compound 38

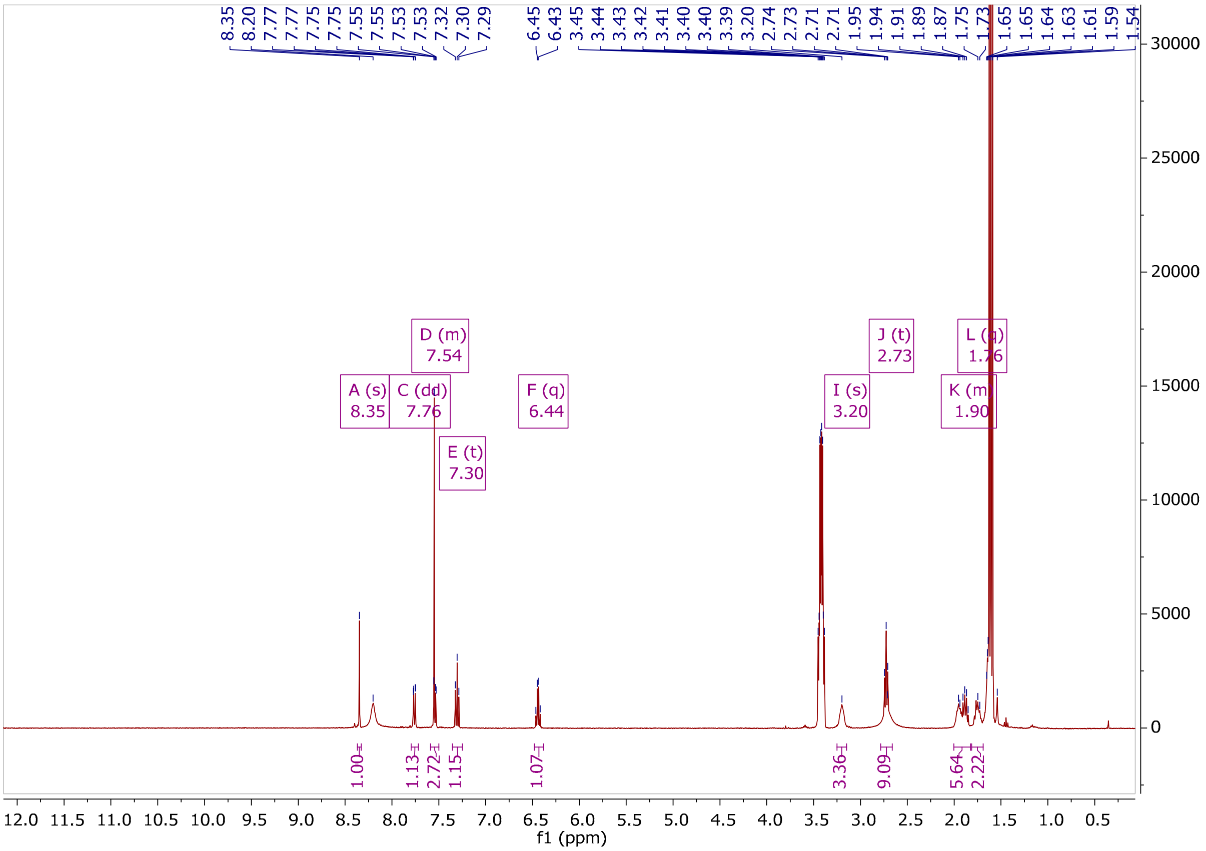
8.35
8.20
7.77
7.77
7.75
7.75
7.55
7.55
7.53
7.53
7.32
7.30
7.29
6.45
6.43
3.45
3.44
3.43
3.42
3.41
3.40
3.40
3.39
3.20
2.74
2.73
2.71
2.71
1.95
1.94
1.91
1.89
1.87
1.75
1.73
1.65
1.65
1.64
1.63
1.61
1.59
1.54
A (s) 8.35
C (dd) 7.76
D (m) 7.54
E (t) 7.30
F (q) 6.44
I (s) 3.20
J (t) 2.73
K (m) 1.90
L (q) 1.76
1.00
1.13
2.72
1.15
1.07
3.36
9.09
5.64
2.22
30000
25000
20000
15000
10000
5000
0
f1 (ppm)

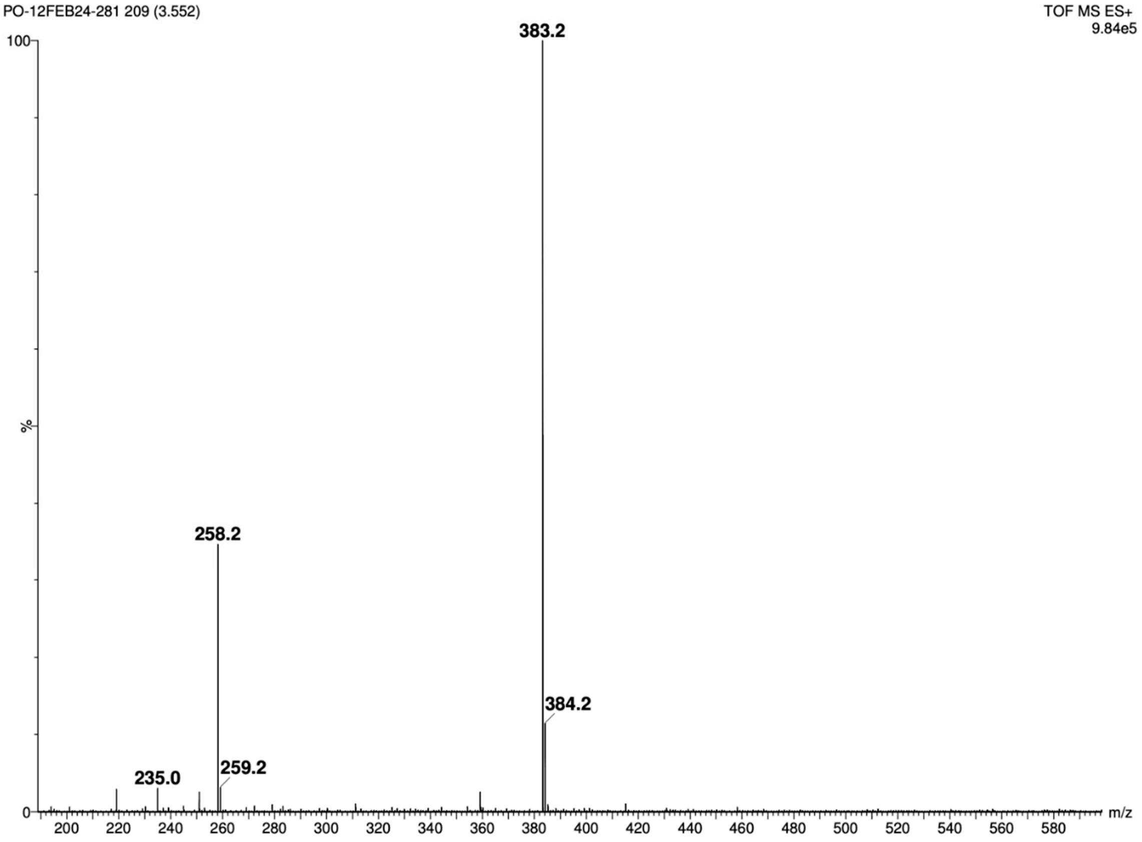

PO-12FEB24-281 209 (3.552)
TOF MS ES+
9.84e5
100
%
0
383.2
258.2
384.2
235.0
259.2
200
220
240
260
280
300
320
340
360
380
400
420
440
460
480
500
520
540
560
580
m/z

Compound 40

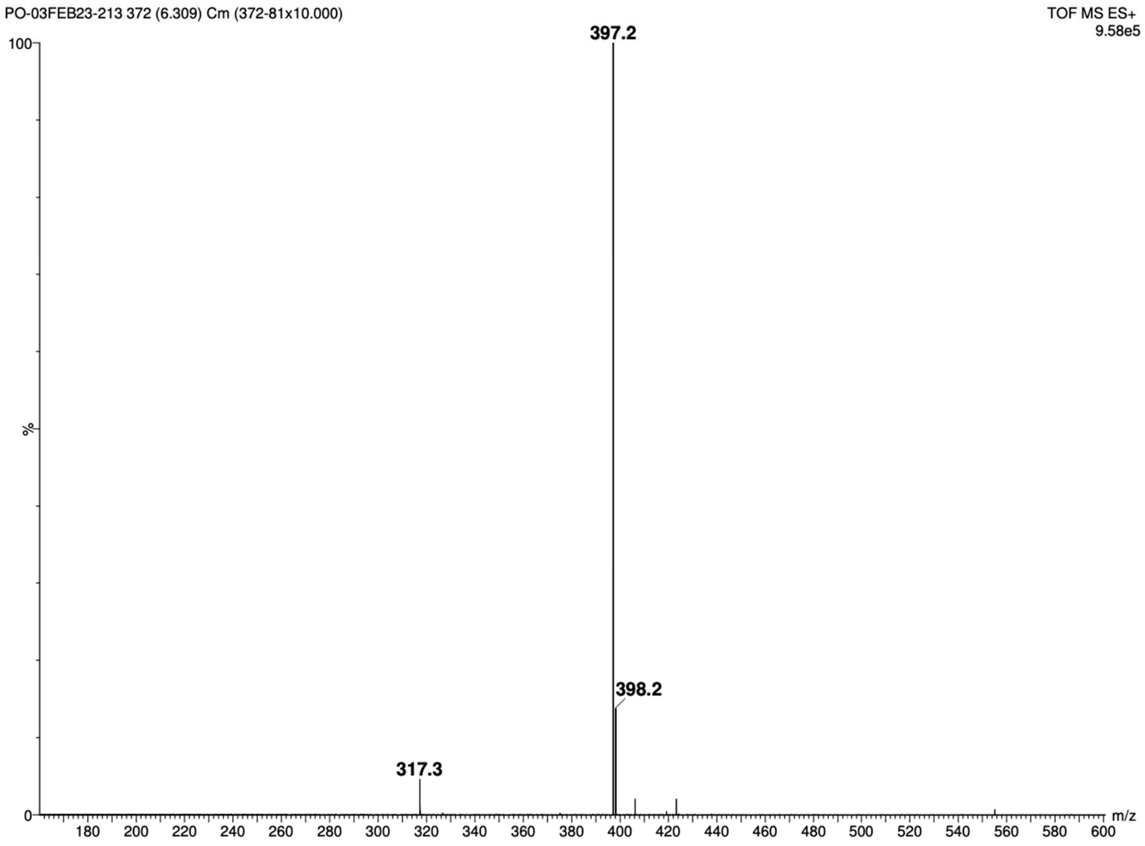
PO-03FEB23-213 372 (6.309) Cm (372-81x10.000)
TOF MS ES+
9.58e5
100
%
0
397.2
398.2
317.3
180
200
220
240
260
280
300
320
340
360
380
400
420
440
460
480
500
520
540
560
580
600
m/z

Data File C:\Chem32\1\Data\PAOLA\Paola 2023-02-02 16-41-38\PO2130000001.D
Sample Name: PO213

===

Acq. Operator : SYSTEM Seq. Line : 1
Acq. Instrument : hplc Location : 1
Injection Date : 2/2/2023 4:43:43 PM Inj : 1
Inj Volume : Inj prog
Method : C:\Chem32\1\Data\PAOLA\Paola 2023-02-02 16-41-38\Paola_A40-90_B_20MIN.M (Sequence Method)
Last changed : 2/2/2023 4:41:38 PM by SYSTEM
Additional Info : Peak(s) manually integrated

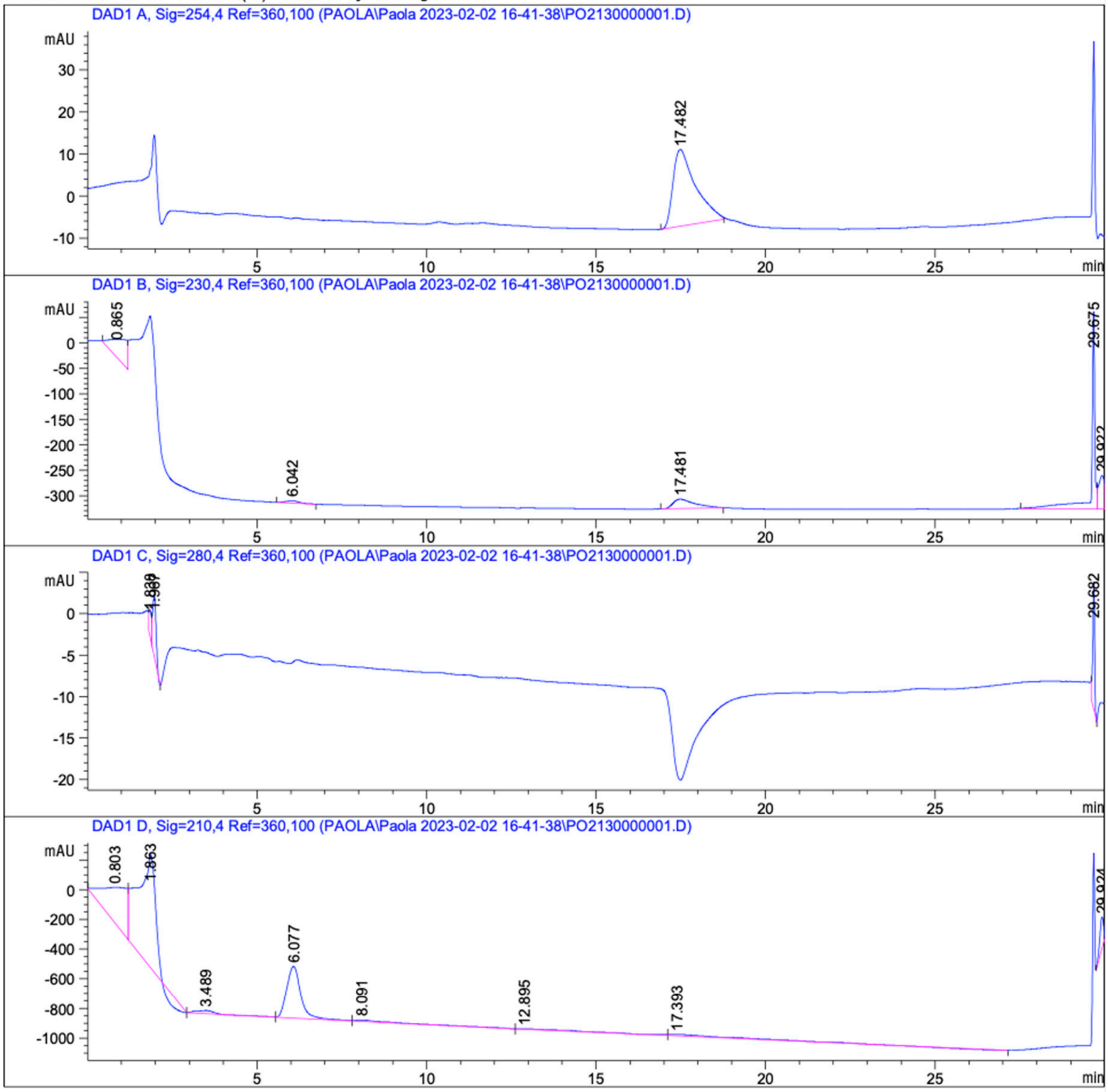

Data File C:\Chem32\1\Data\PAOLA\Paola 2023-02-02 16-41-38\P02130000001.D
Sample Name: P0213

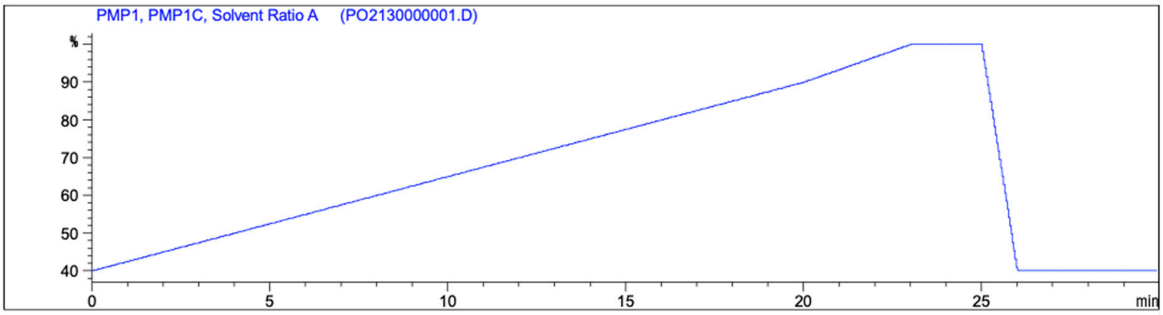

===
External Standard Report
===

Sorted By : Signal
Multiplier : 1.0000
Dilution : 1.0000
Sample Amount: : 1.00000 [wt%] (not used in calc.)
Do not use Multiplier & Dilution Factor with ISTDs
===
===
Area Percent Report
===

Sorted By : Signal
Multiplier : 1.0000
Dilution : 1.0000
Sample Amount: : 1.00000 [wt%] (not used in calc.)
Do not use Multiplier & Dilution Factor with ISTDs

Signal 1: DAD1 A, Sig=254,4 Ref=360,100

Peak #	RetTime [min]	Type	Width [min]	Area [mAU*s]	Height [mAU]	Area %
1	17.482	BB	0.6604	841.19678	18.22662	100.0000

Totals : 841.19678 18.22662

Signal 2: DAD1 B, Sig=230,4 Ref=360,100

Peak #	RetTime [min]	Type	Width [min]	Area [mAU*s]	Height [mAU]	Area %
1	0.865	BV	0.4719	1394.88184	35.53668	24.8425
2	6.042	BB	0.3853	114.31191	4.34005	2.0359
3	17.481	BB	0.6589	861.17731	18.71270	15.3374
4	29.675	BV	0.0918	2535.57935	390.48315	45.1581
5	29.922	VBA	0.1820	708.94067	64.91125	12.6261

Totals : 5614.89108 513.98384

Compound 19

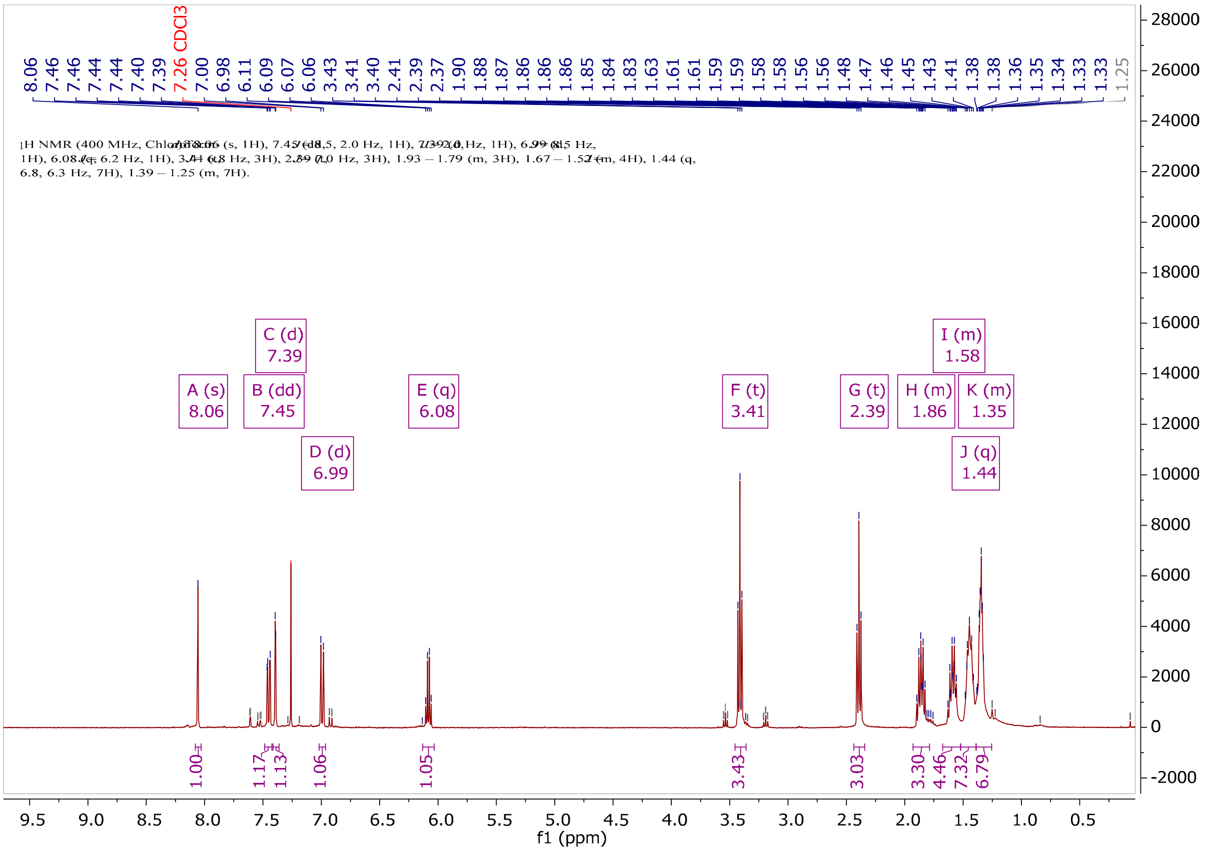

CDCl3
A (s) 8.06
B (dd) 7.45
C (d) 7.39
D (d) 6.99
E (q) 6.08
F (t) 3.41
G (t) 2.39
H (m) 1.86
I (m) 1.58
J (q) 1.44
K (m) 1.35
f1 (ppm)

Data File C:\Chem32\1\Data\PAOLA\Paola 2023-05-03 10-04-44\P0218-30000001.D
Sample Name: P0218-3

===
Area Percent Report
===

Sorted By : Signal
Multiplier : 1.0000
Dilution : 1.0000
Sample Amount: : 1.00000 [ng/ul] (not used in calc.)
Use Multiplier & Dilution Factor with ISTDs

Signal 1: DAD1 A, Sig=254,4 Ref=360,100

Peak #	RetTime [min]	Type	Width [min]	Area [mAU*s]	Height [mAU]	Area %
1	23.097	MM	0.3950	140.94206	5.94758	4.6888
2	24.380	BB	0.3876	2864.98804	112.30928	95.3112

Totals : 3005.93010 118.25686

Signal 2: DAD1 D, Sig=210,4 Ref=360,100

Peak #	RetTime [min]	Type	Width [min]	Area [mAU*s]	Height [mAU]	Area %
1	0.366	BB	0.5026	4423.65820	128.82455	2.4779
2	2.084	BB	0.2766	129.19249	7.74556	0.0724
3	2.538	BV E	0.2522	380.93347	23.87127	0.2134
4	2.974	VV E	0.3115	847.86060	39.33240	0.4749
5	3.598	VV E	0.5969	3451.42285	77.30439	1.9333
6	5.348	VV E	1.6834	1.45447e4	106.63036	8.1473
7	7.549	VB R	0.9189	1.38720e5	1804.47217	77.7052
8	19.876	BB	2.0363	1608.28027	9.57182	0.9009
9	24.371	BB	0.3085	1126.34033	58.52559	0.6309
10	25.598	BV R	0.3685	1.32444e4	485.21701	7.4190
11	26.380	VB E	0.2108	44.17799	2.98297	0.0247

Totals : 1.78521e5 2744.47809

Signal 3: DAD1 G, Sig=270,4 Ref=360,100

Peak #	RetTime [min]	Type	Width [min]	Area [mAU*s]	Height [mAU]	Area %
1	23.101	BB	0.3866	51.97716	1.85675	2.0845
2	24.380	BB	0.3866	2441.47510	96.02641	97.9155

Totals : 2493.45226 97.88316

===
*** End of Report ***

Compound 20

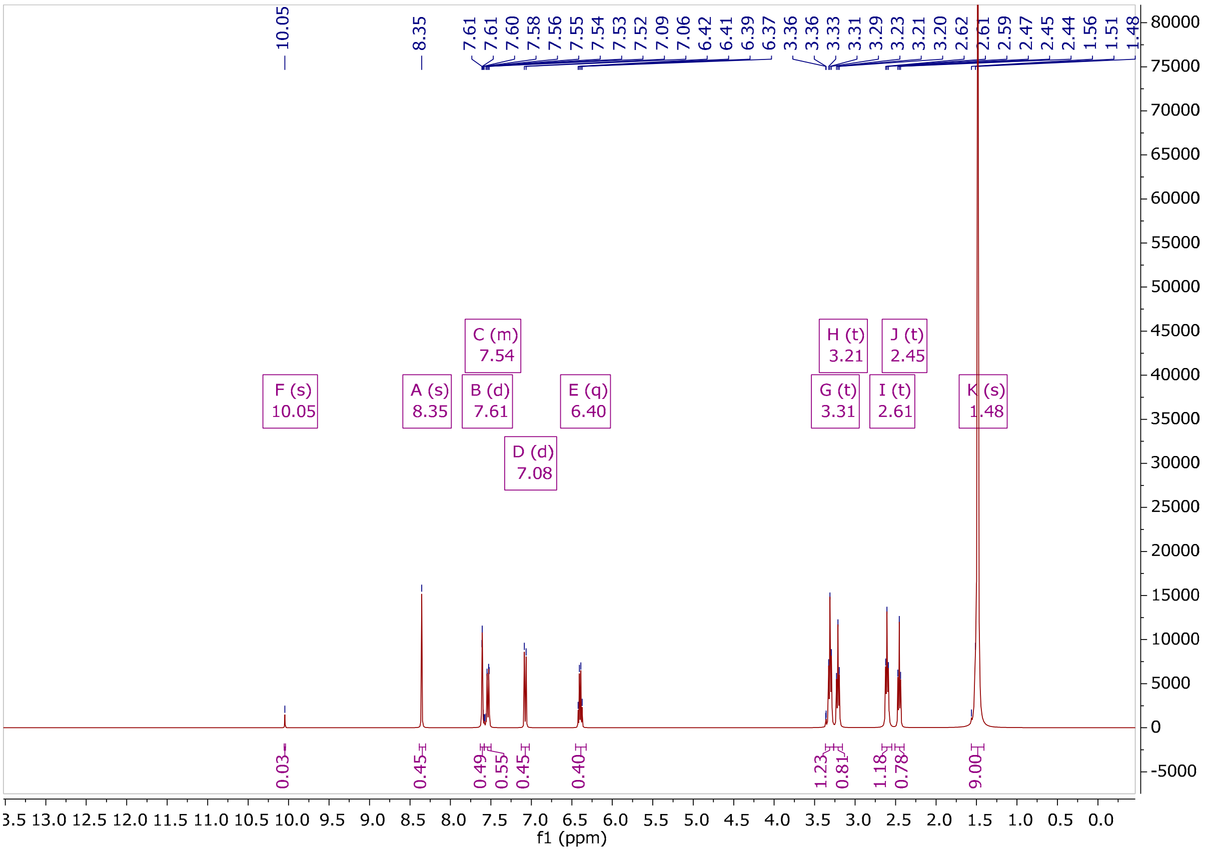

F (s) 10.05
A (s) 8.35
B (d) 7.61
C (m) 7.54
D (d) 7.08
E (q) 6.40
G (t) 3.31
H (t) 3.21
I (t) 2.61
J (t) 2.45
K (s) 1.48
f1 (ppm)

Compound 22

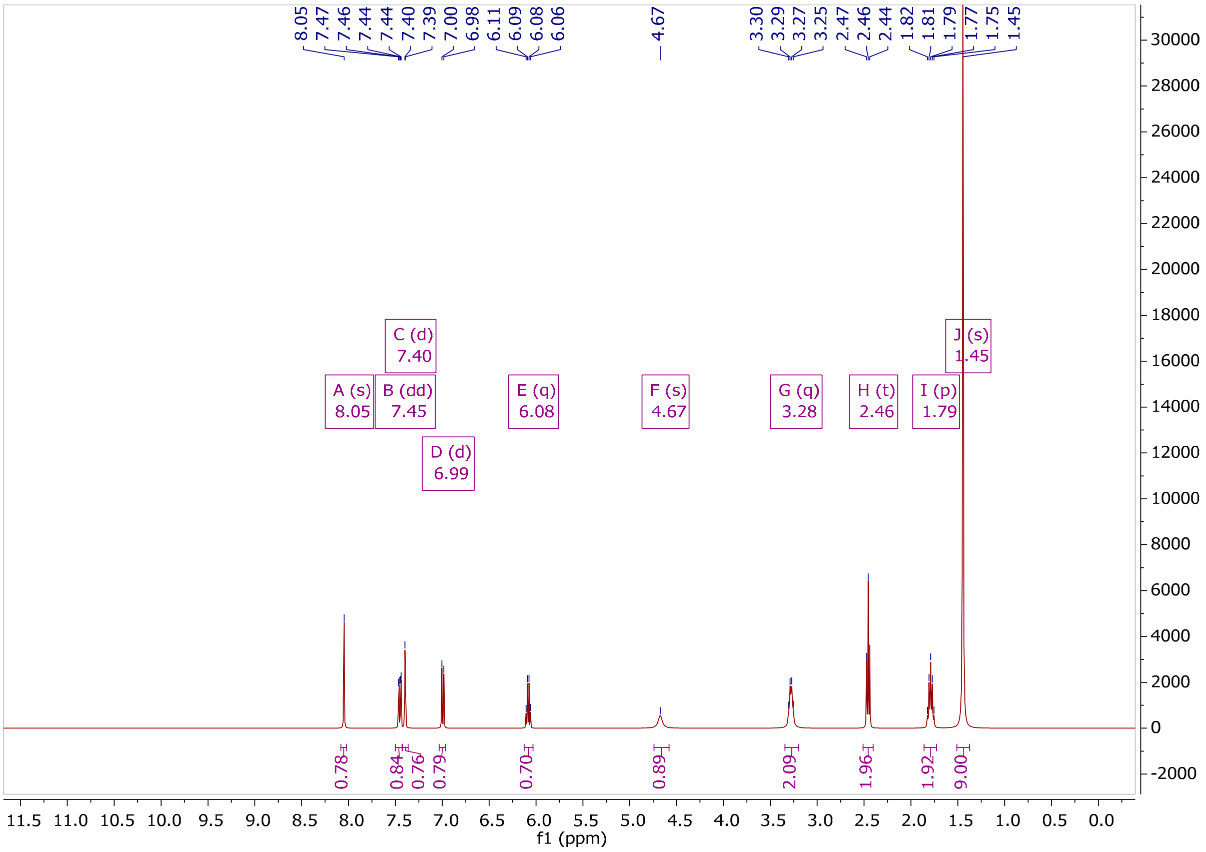
8.05
7.47
7.46
7.44
7.44
7.40
7.39
7.00
6.98
6.11
6.09
6.08
6.06
4.67
3.30
3.29
3.27
3.25
2.47
2.46
2.44
1.82
1.81
1.79
1.77
1.75
1.45
A (s) 8.05
B (dd) 7.45
C (d) 7.40
D (d) 6.99
E (q) 6.08
F (s) 4.67
G (q) 3.28
H (t) 2.46
I (p) 1.79
J (s) 1.45
0.78
0.84
0.76
0.79
0.70
0.89
2.09
1.96
1.92
9.00
f1 (ppm)

Compound 24

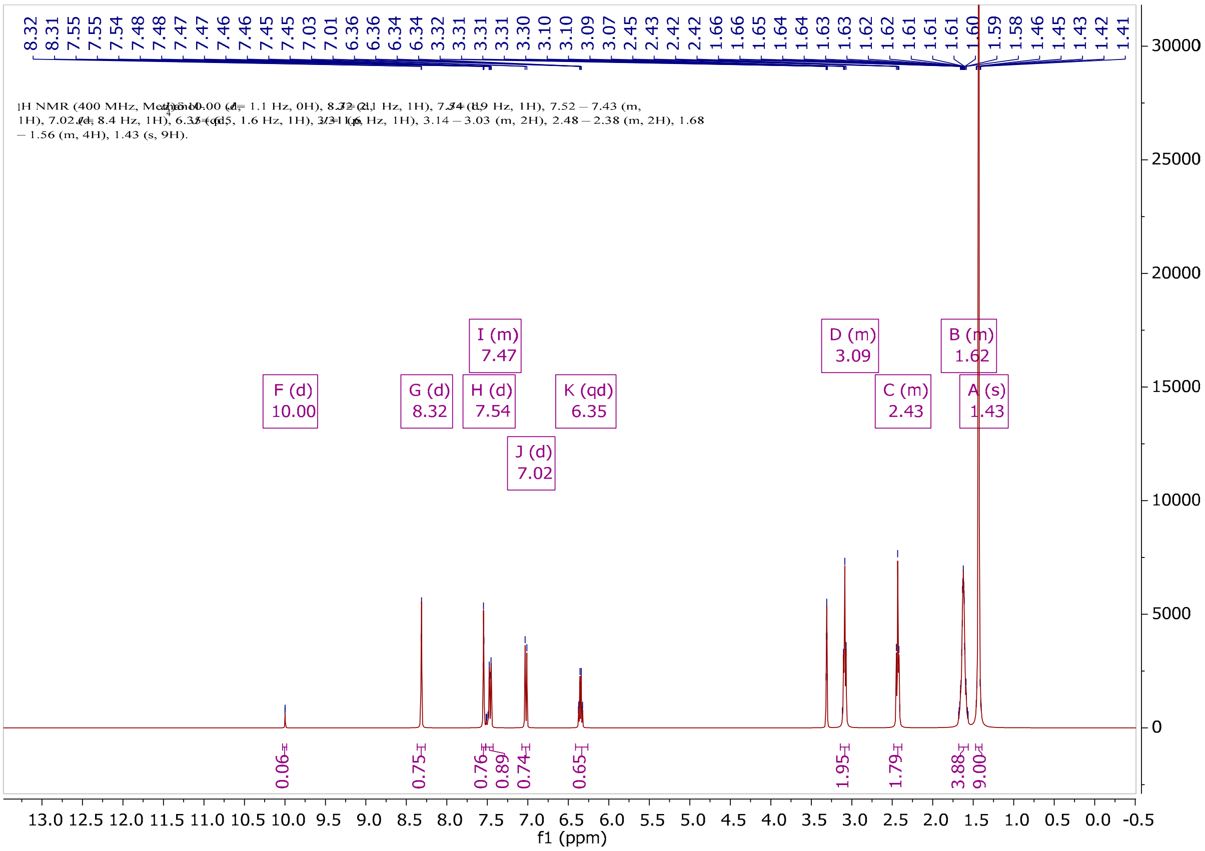

1H NMR (400 MHz, Methanol-d4) δ 10.00 (d, J = 1.1 Hz, 0H), 8.32 (d, J = 2.1 Hz, 1H), 7.54 (d, J = 1.9 Hz, 1H), 7.52 – 7.43 (m, 1H), 7.02 (d, J = 8.4 Hz, 1H), 6.35 (qd, J = 3.5, 1.6 Hz, 1H), 3.31 (p, J = 1.6 Hz, 1H), 3.14 – 3.03 (m, 2H), 2.48 – 2.38 (m, 2H), 1.68 – 1.56 (m, 4H), 1.43 (s, 9H).
F (d) 10.00
G (d) 8.32
H (d) 7.54
I (m) 7.47
J (d) 7.02
K (qd) 6.35
D (m) 3.09
C (m) 2.43
B (m) 1.62
A (s) 1.43
f1 (ppm)

```
Data File C:\Chem32\1\Data\PAOLA\Paola 2023-10-06 16-11-29\PO2440000003.D
Sample Name: PO244

    =====================================================================
    Acq. Operator   : SYSTEM                          Seq. Line :    1
    Acq. Instrument : hplc                             Location :    1
    Injection Date  : 10/6/2023 4:17:57 PM                  Inj :    1
                                                     Inj Volume : Inj prog
    Method          : C:\Chem32\1\Data\PAOLA\Paola 2023-10-06 16-11-29\AN_A05-95_B95-05_20MIN.M (
                      Sequence Method)
    Last changed    : 10/6/2023 4:11:30 PM by SYSTEM
    Additional Info : Peak(s) manually integrated
```

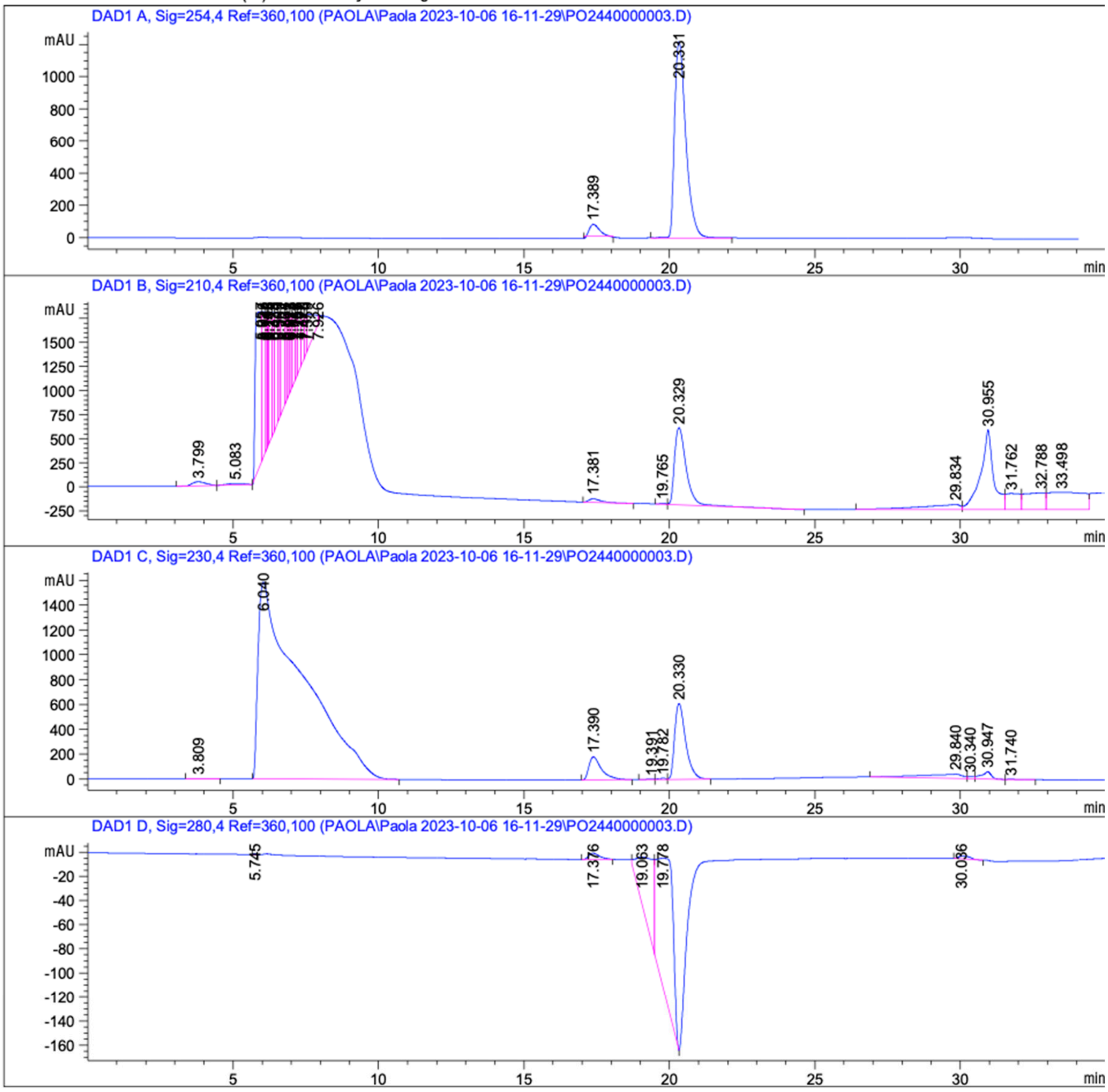

Data File C:\Chem32\1\Data\PAOLA\Paola 2023-10-06 16-11-29\PO2440000003.D
Sample Name: PO244

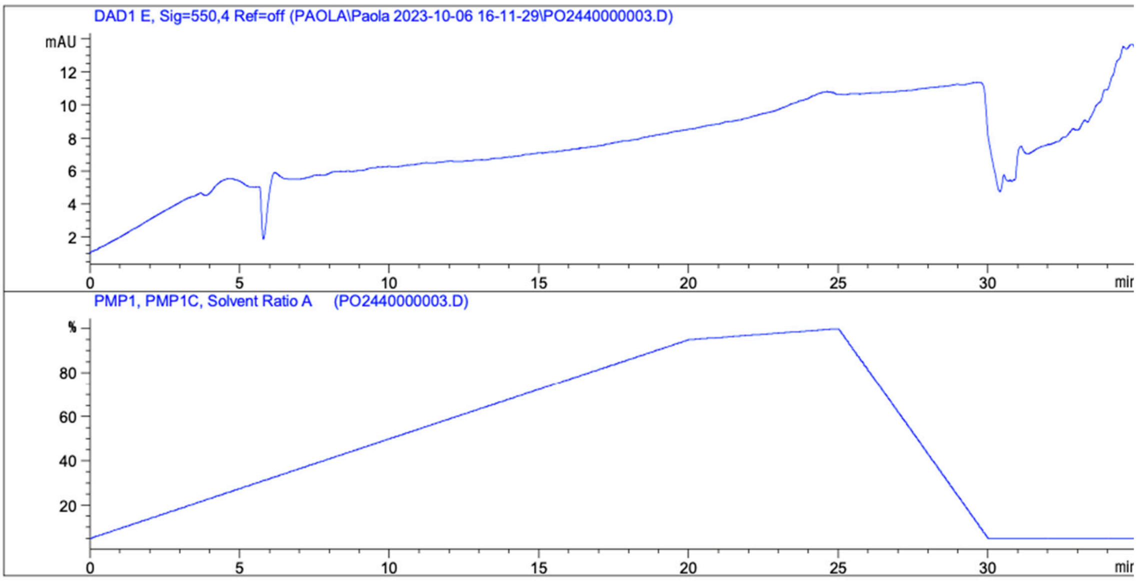

===

Fraction Information

===

Fraction collection using a timetable

===

No Fractions found.

===

===

External Standard Report

===

Sorted By : Signal
Multiplier : 1.0000
Dilution : 1.0000
Sample Amount: : 1.00000 [wt%] (not used in calc.)
Do not use Multiplier & Dilution Factor with ISTDs

===

===

Area Percent Report

===

Sorted By : Signal
Multiplier : 1.0000
Dilution : 1.0000
Sample Amount: : 1.00000 [wt%] (not used in calc.)
Do not use Multiplier & Dilution Factor with ISTDs

Signal 1: DAD1 A, Sig=254,4 Ref=360,100

Data File C:\Chem32\1\Data\PAOLA\Paola 2023-10-06 16-11-29\P02440000003.D
Sample Name: P0244

Peak #	RetTime [min]	Type	Width [min]	Area [mAU*s]	Height [mAU]	Area %
1	17.389	MM	0.3933	1724.74316	73.09673	4.9711
2	20.331	MM	0.4510	3.29706e4	1218.43237	95.0289

Totals : 3.46954e4 1291.52910

Signal 2: DAD1 B, Sig=210,4 Ref=360,100

Peak #	RetTime [min]	Type	Width [min]	Area [mAU*s]	Height [mAU]	Area %
1	3.799	BV	0.5114	1350.37708	41.74247	0.6611
2	5.083	VV	0.5467	688.67450	16.30764	0.3372
3	5.957	VV	0.1858	2.43314e4	1579.22083	11.9123
4	6.028	VV	0.1027	1.18680e4	1526.86743	5.8104
5	6.142	VV	0.0557	4812.56348	1438.87146	2.3562
6	6.195	VV	0.0504	4228.11865	1398.16333	2.0700
7	6.245	VV	0.0805	8685.67285	1360.32910	4.2524
8	6.375	VV	0.0620	5398.75977	1260.62915	2.6432
9	6.434	VV	0.1012	9930.24902	1216.56982	4.8617
10	6.569	VV	0.0791	5280.72803	1112.46301	2.5854
11	6.724	VV	0.1308	9638.96191	996.62787	4.7191
12	6.807	VV	0.0585	4357.46484	933.07550	2.1334
13	6.912	VV	0.0642	3824.73975	854.85858	1.8725
14	6.973	VV	0.0680	3883.98511	808.52246	1.9015
15	7.072	VV	0.0770	4467.48535	734.23932	2.1872
16	7.166	VV	0.0574	2698.19360	663.82324	1.3210
17	7.237	VV	0.0920	4504.05908	610.70618	2.2051
18	7.370	VV	0.0787	3183.90869	510.97433	1.5588
19	7.475	VV	0.0724	2448.66187	430.31445	1.1988
20	7.559	VV	0.1818	5521.99951	366.49240	2.7035
21	7.926	VB	0.0381	239.18648	86.83878	0.1171
22	17.381	BB	0.4322	1039.07446	36.49131	0.5087
23	19.765	BV	0.2849	117.77124	6.64063	0.0577
24	20.329	VB	0.4324	2.24284e4	801.62579	10.9806
25	29.834	BV	1.1422	4639.61230	51.05762	2.2715
26	30.955	VV	0.4042	2.53263e4	824.81183	12.3994
27	31.762	VV	0.4639	5630.92285	165.10036	2.7568
28	32.788	VV	0.6179	8385.00684	172.85812	4.1052
29	33.498	VV	1.0172	1.53441e4	177.03101	7.5123

Totals : 2.04254e5 2.01833e4

Signal 3: DAD1 C, Sig=230,4 Ref=360,100

Peak #	RetTime [min]	Type	Width [min]	Area [mAU*s]	Height [mAU]	Area %
1	3.809	BB	0.3960	79.86488	2.60285	0.0410
2	6.040	BB	1.3490	1.67523e5	1582.55347	86.0111
3	17.390	BB	0.4547	5658.26221	186.09598	2.9051

Compound 28

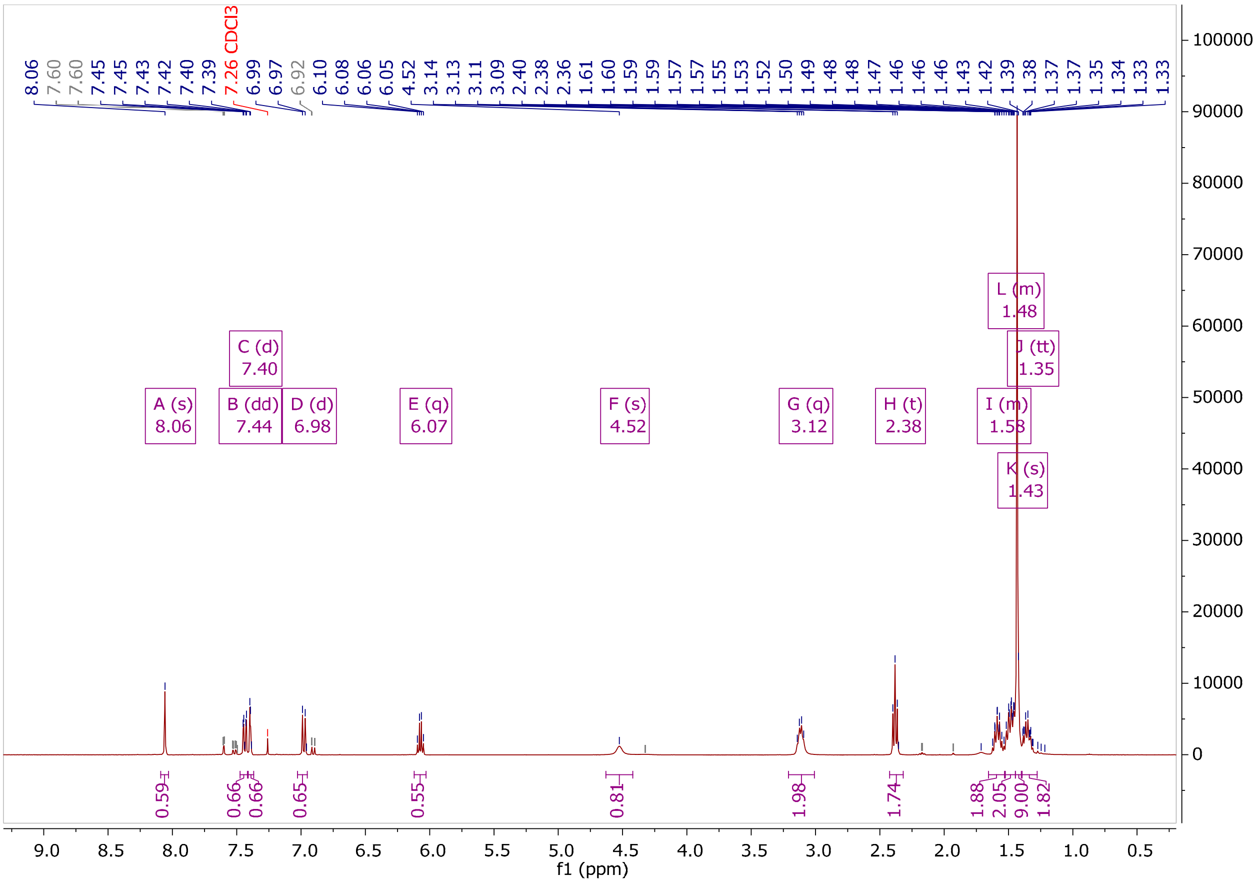

A (s) 8.06
B (dd) 7.44
C (d) 7.40
D (d) 6.98
E (q) 6.07
F (s) 4.52
G (q) 3.12
H (t) 2.38
I (m) 1.58
J (tt) 1.35
K (s) 1.43
L (m) 1.48
7.26 CDCl3
f1 (ppm)

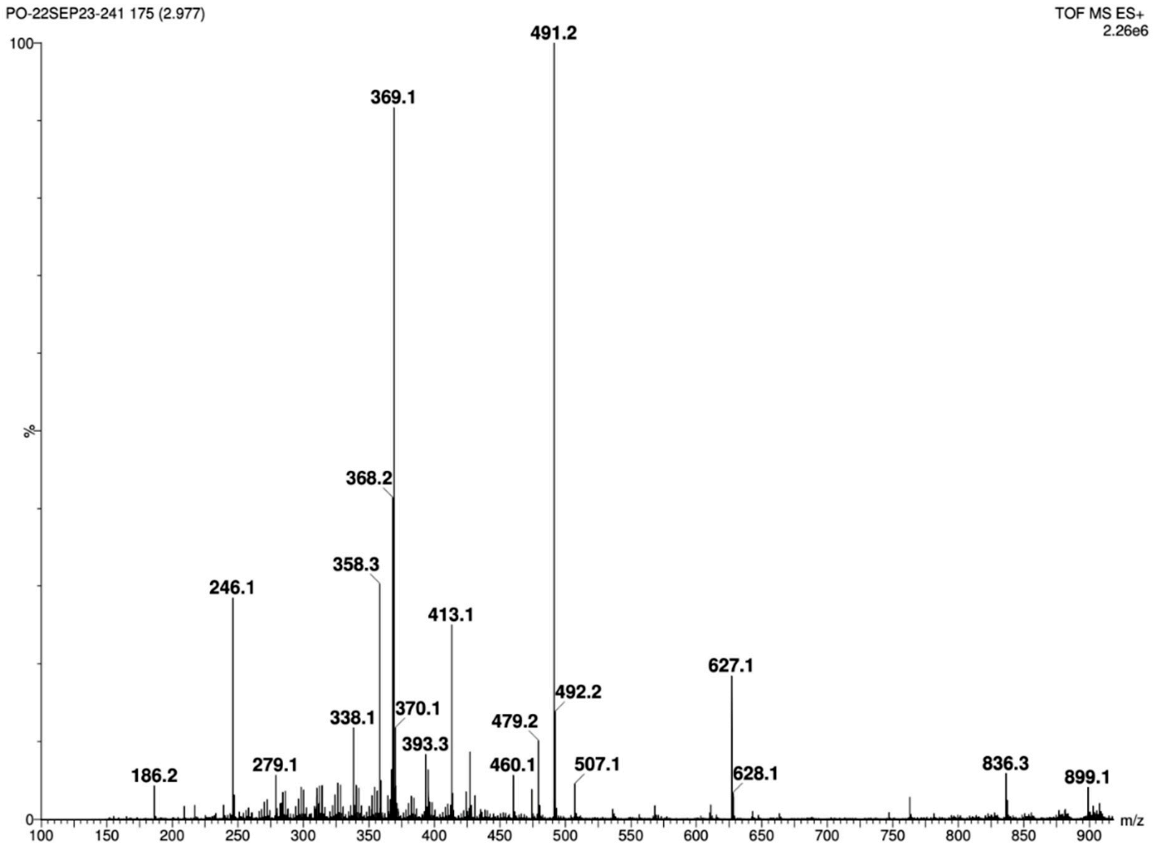
PO-22SEP23-241 175 (2.977)
TOF MS ES+
2.26e6
100
%
0
491.2
369.1
368.2
358.3
246.1
413.1
627.1
492.2
338.1
370.1
479.2
393.3
279.1
460.1
507.1
186.2
628.1
836.3
899.1
m/z
100
150
200
250
300
350
400
450
500
550
600
650
700
750
800
850
900

Data File C:\Chem32\1\Data\PAOLA\Paola 2023-09-21 18-33-51\PO240000002.D
Sample Name: PO241

===
Acq. Operator : SYSTEM Seq. Line : 2
Acq. Instrument : hplc Location : 2
Injection Date : 9/21/2023 7:13:19 PM Inj : 1
Inj Volume : 5.000 µl
Method : C:\Chem32\1\Data\PAOLA\Paola 2023-09-21 18-33-51\Paola_A75-100_B_20MIN.M (Sequence Method)
Last changed : 9/21/2023 6:33:52 PM by SYSTEM
Additional Info : Peak(s) manually integrated

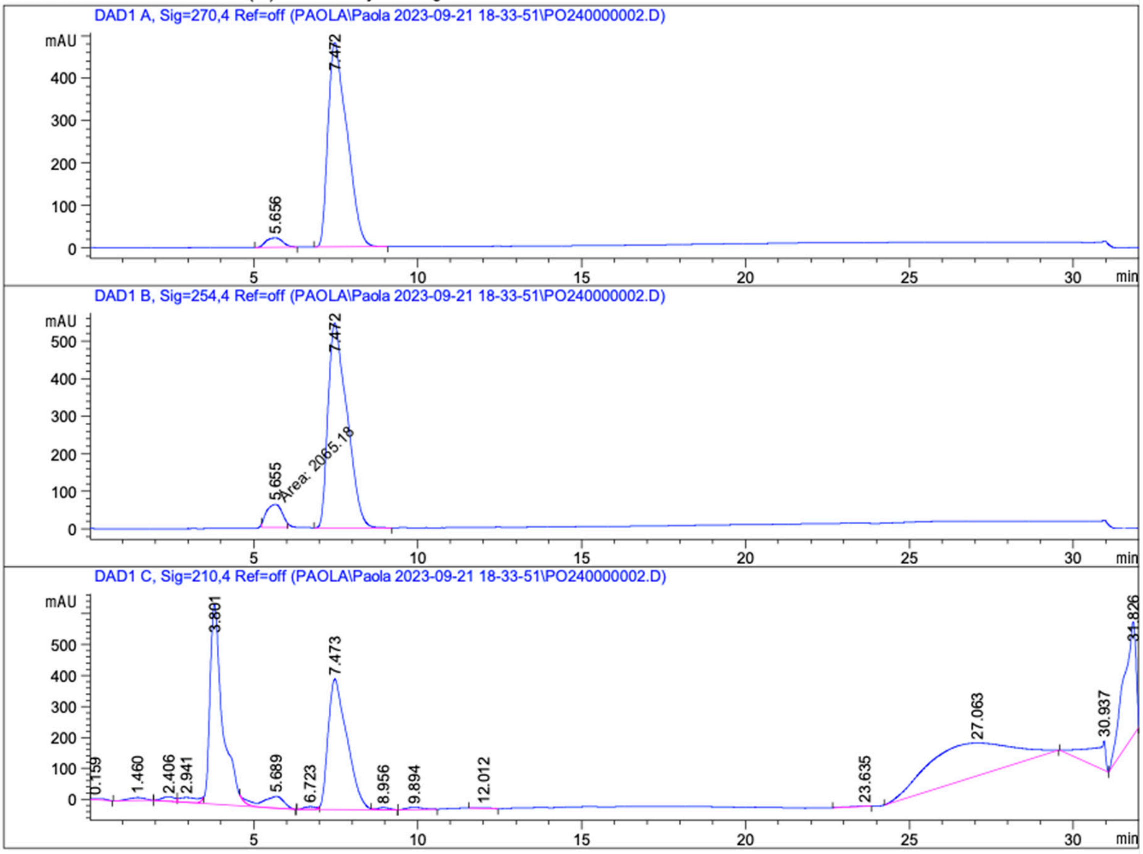

===
Fraction Information
===
Fraction collection off
===
No Fractions found.
===

Data File C:\Chem32\1\Data\PAOLA\Paola 2023-09-21 18-33-51\P0240000002.D
Sample Name: P0241

===
Area Percent Report
===

Sorted By : Signal
Multiplier : 1.0000
Dilution : 1.0000
Sample Amount: : 1.00000 [ng/ul] (not used in calc.)
Use Multiplier & Dilution Factor with ISTDs

Signal 1: DAD1 A, Sig=270,4 Ref=off

Peak #	RetTime [min]	Type	Width [min]	Area [mAU*s]	Height [mAU]	Area %
1	5.656	BB	0.5767	768.46936	22.04261	3.8135
2	7.472	BB	0.5826	1.93827e4	482.59418	96.1865

Totals : 2.01511e4 504.63679

Signal 2: DAD1 B, Sig=254,4 Ref=off

Peak #	RetTime [min]	Type	Width [min]	Area [mAU*s]	Height [mAU]	Area %
1	5.655	MM	0.5526	2065.17847	62.28187	8.6234
2	7.472	BB	0.5838	2.18834e4	545.77917	91.3766

Totals : 2.39486e4 608.06104

Signal 3: DAD1 C, Sig=210,4 Ref=off

Peak #	RetTime [min]	Type	Width [min]	Area [mAU*s]	Height [mAU]	Area %
1	0.159	BB	0.4203	114.76994	3.43107	0.1600
2	1.460	BB	0.4804	309.72495	9.16107	0.4317
3	2.406	BV E	0.4098	396.92261	14.57103	0.5533
4	2.941	VV E	0.5192	615.80634	15.31344	0.8584
5	3.801	VV R	0.3960	1.75163e4	646.73584	24.4160
6	5.689	VB E	0.6526	1786.64673	37.71822	2.4904
7	6.723	BV E	0.3714	208.21078	8.95078	0.2902
8	7.473	VV R	0.5840	1.68956e4	421.22745	23.5509
9	8.956	VB E	0.3722	165.64168	6.89705	0.2309
10	9.894	BB	0.4515	239.05855	7.01711	0.3332
11	12.012	BB	0.3547	61.91063	2.29510	0.0863
12	23.635	BB	0.4890	71.77733	1.82432	0.1001
13	27.063	BB	2.2358	2.01888e4	106.10860	28.1412
14	30.937	BB	0.4315	3256.31494	93.23711	4.5390
15	31.826	BBA	0.3649	9913.58008	369.67438	13.8186

Totals : 7.17410e4 1744.16255

Compound 30

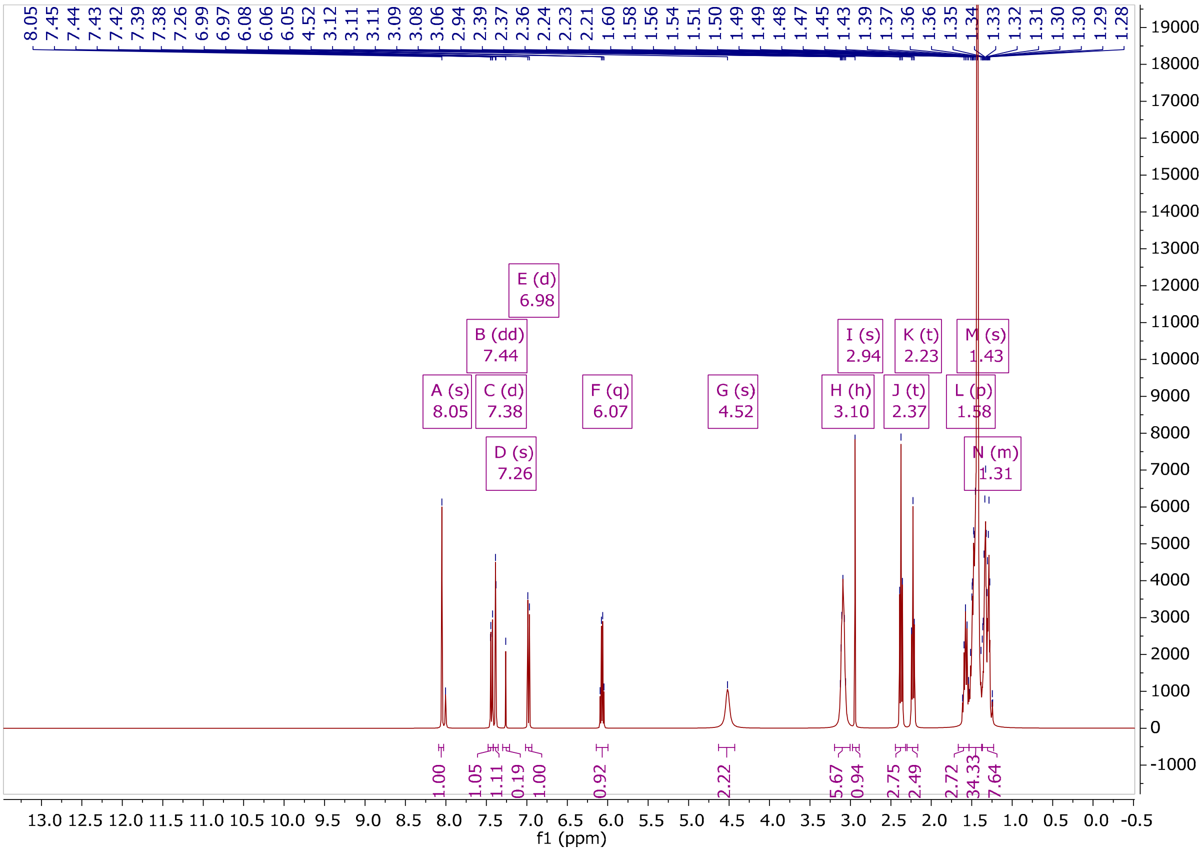
8.05
7.45
7.44
7.43
7.42
7.39
7.38
7.26
6.99
6.97
6.08
6.06
6.05
4.52
3.12
3.11
3.11
3.09
3.08
3.06
2.94
2.39
2.37
2.36
2.24
2.23
2.21
1.60
1.58
1.56
1.54
1.51
1.50
1.49
1.49
1.48
1.47
1.45
1.43
1.39
1.37
1.36
1.36
1.35
1.34
1.33
1.32
1.31
1.30
1.30
1.29
1.28
A (s) 8.05
B (dd) 7.44
C (d) 7.38
D (s) 7.26
E (d) 6.98
F (q) 6.07
G (s) 4.52
H (h) 3.10
I (s) 2.94
J (t) 2.37
K (t) 2.23
L (p) 1.58
M (s) 1.43
N (m) 1.31
1.00
1.05
1.11
0.19
1.00
0.92
2.22
5.67
0.94
2.75
2.49
2.72
34.33
7.64
f1 (ppm)

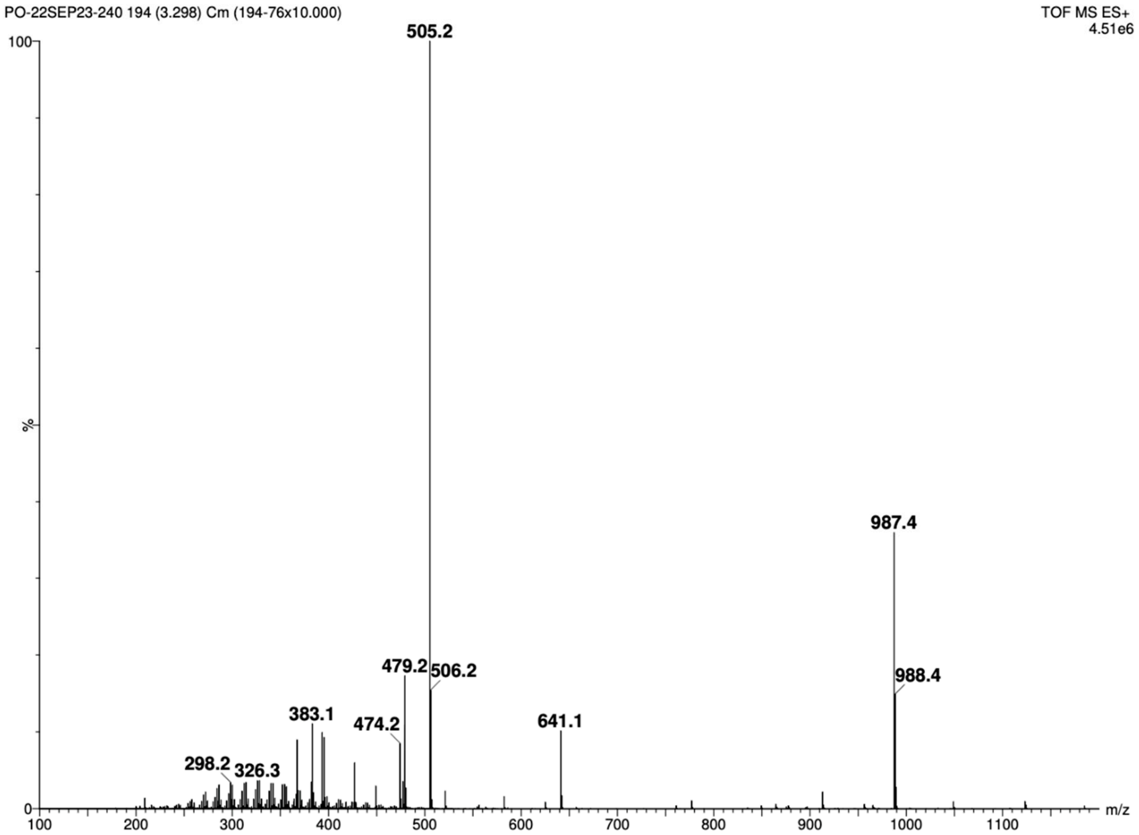
PO-22SEP23-240 194 (3.298) Cm (194-76x10.000)
TOF MS ES+
4.51e6
505.2
987.4
479.2
506.2
988.4
383.1
474.2
641.1
298.2
326.3
%
m/z

Data File C:\Chem32\1\Data\PAOLA\Paola 2023-10-06 10-42-24\PO2400000006.D
Sample Name: PO240

===

Acq. Operator	: SYSTEM	Seq. Line :	1
Acq. Instrument	: hplc	Location :	1
Injection Date	: 10/6/2023 10:48:42 AM	Inj :	1
		Inj Volume :	Inj prog

Method : C:\Chem32\1\Data\PAOLA\Paola 2023-10-06 10-42-24\AN_A05-95_B95-05_20MIN.M (Sequence Method)
Last changed : 10/6/2023 10:42:25 AM by SYSTEM
Additional Info : Peak(s) manually integrated

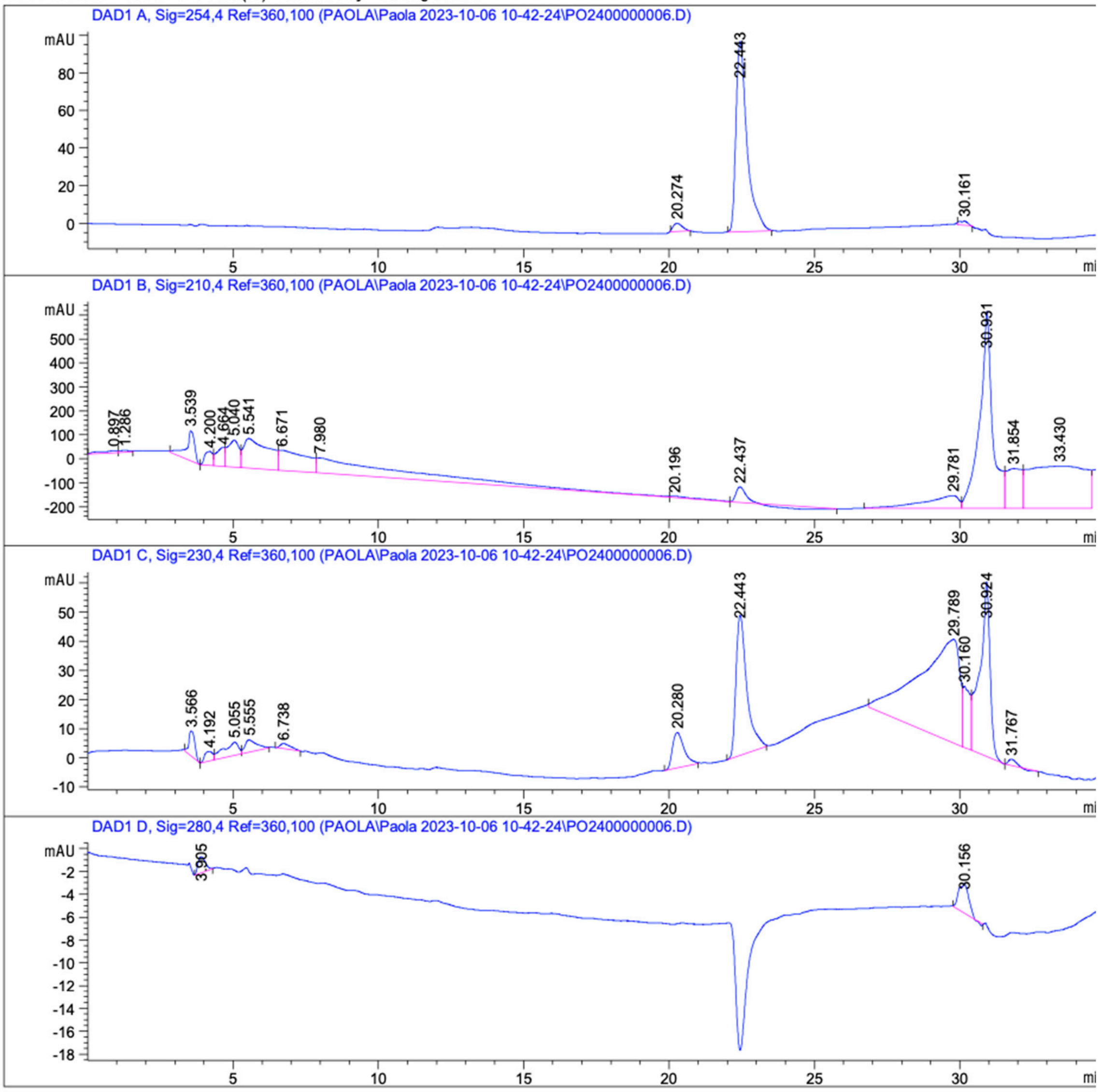

Data File C:\Chem32\1\Data\PAOLA\Paola 2023-10-06 10-42-24\PO2400000006.D
Sample Name: PO240

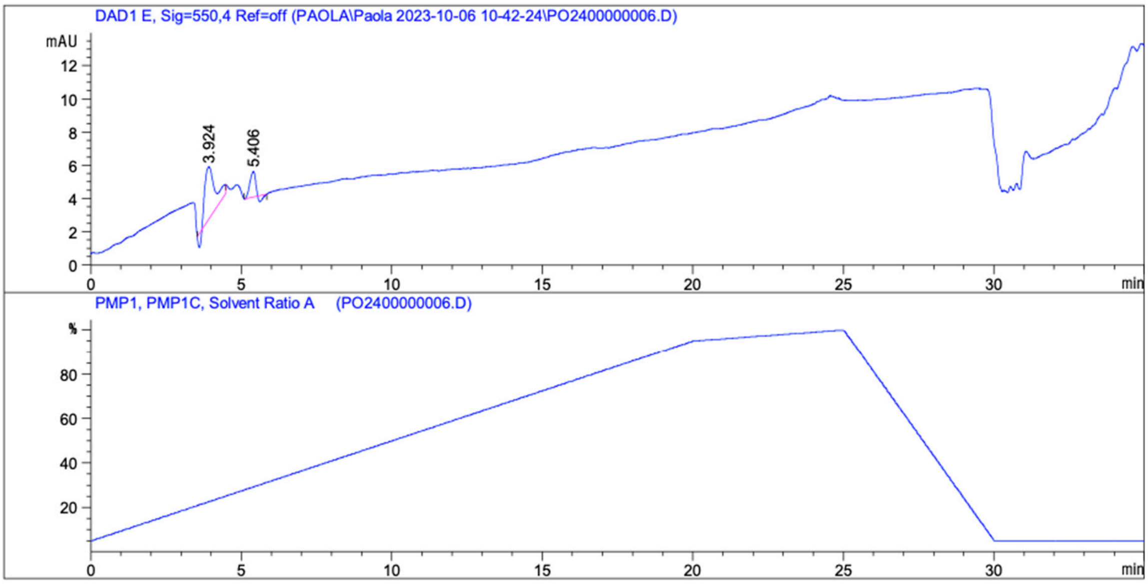


```
=====================================================================================
                            Fraction Information
=====================================================================================
Fraction collection using a timetable
=====================================================================================
No Fractions found.
=====================================================================================
=====================================================================
                      External Standard Report
=====================================================================

Sorted By             :      Signal
Multiplier            :      1.0000
Dilution              :      1.0000
Sample Amount:               :      1.00000  [wt%]   (not used in calc.)
Do not use Multiplier & Dilution Factor with ISTDs
=====================================================================
=====================================================================
                         Area Percent Report
=====================================================================

Sorted By             :      Signal
Multiplier            :      1.0000
Dilution              :      1.0000
Sample Amount:               :      1.00000  [wt%]   (not used in calc.)
Do not use Multiplier & Dilution Factor with ISTDs


Signal 1: DAD1 A, Sig=254,4 Ref=360,100
```

Data File C:\Chem32\1\Data\PAOLA\Paola 2023-10-06 10-42-24\P02400000006.D
Sample Name: P0240

Peak #	RetTime [min]	Type	Width [min]	Area [mAU*s]	Height [mAU]	Area %
1	20.274	MM	0.3656	92.14151	4.20003	3.2533
2	22.443	BB	0.3984	2697.90186	101.38781	95.2552
3	30.161	MM	0.3093	42.24364	2.27615	1.4915

Totals : 2832.28701 107.86399

Signal 2: DAD1 B, Sig=210,4 Ref=360,100

Peak #	RetTime [min]	Type	Width [min]	Area [mAU*s]	Height [mAU]	Area %
1	0.897	BV	0.6748	419.51044	8.03902	0.4309
2	1.286	VV	0.3504	193.40675	7.82277	0.1986
3	3.539	BV	0.3000	2584.47998	125.62318	2.6545
4	4.200	VV	0.2994	1114.37805	58.19474	1.1446
5	4.664	VV	0.2825	1556.87244	78.81134	1.5991
6	5.040	VV	0.3781	3058.25024	112.87090	3.1412
7	5.541	VV	0.7894	7502.48779	123.37865	7.7059
8	6.671	VV	0.8398	5587.11572	84.02007	5.7386
9	7.980	VV	2.8309	1.47473e4	62.67968	15.1471
10	20.196	VV	0.7840	383.36758	5.86952	0.3938
11	22.437	VB	0.1493	338.14664	64.33326	0.3473
12	29.781	BV	1.0554	4384.78320	52.44699	4.5036
13	30.931	VV	0.4053	2.53334e4	822.61554	26.0202
14	31.854	VV	0.5074	6112.56787	165.30826	6.2783
15	33.430	VV	1.6203	2.40446e4	176.39705	24.6964

Totals : 9.73608e4 1948.41097

Signal 3: DAD1 C, Sig=230,4 Ref=360,100

Peak #	RetTime [min]	Type	Width [min]	Area [mAU*s]	Height [mAU]	Area %
1	3.566	BV	0.2300	119.68671	8.51389	1.6220
2	4.192	VV	0.2517	61.93170	3.25909	0.8393
3	5.055	VV	0.4939	170.00294	4.52929	2.3039
4	5.555	VB	0.3964	121.68197	4.17059	1.6491
5	6.738	BB	0.3686	45.06836	1.74930	0.6108
6	20.280	BB	0.4258	333.99835	12.10789	4.5265
7	22.443	BB	0.3935	1259.40881	48.08889	17.0679
8	29.789	BV	1.1919	3399.16211	35.47557	46.0666
9	30.160	VV	0.2177	357.03348	21.07438	4.8386
10	30.924	VB	0.3269	1475.65442	60.00426	19.9986
11	31.767	BB	0.2463	35.16769	2.22594	0.4766

Totals : 7378.79655 201.19910

Compound 33

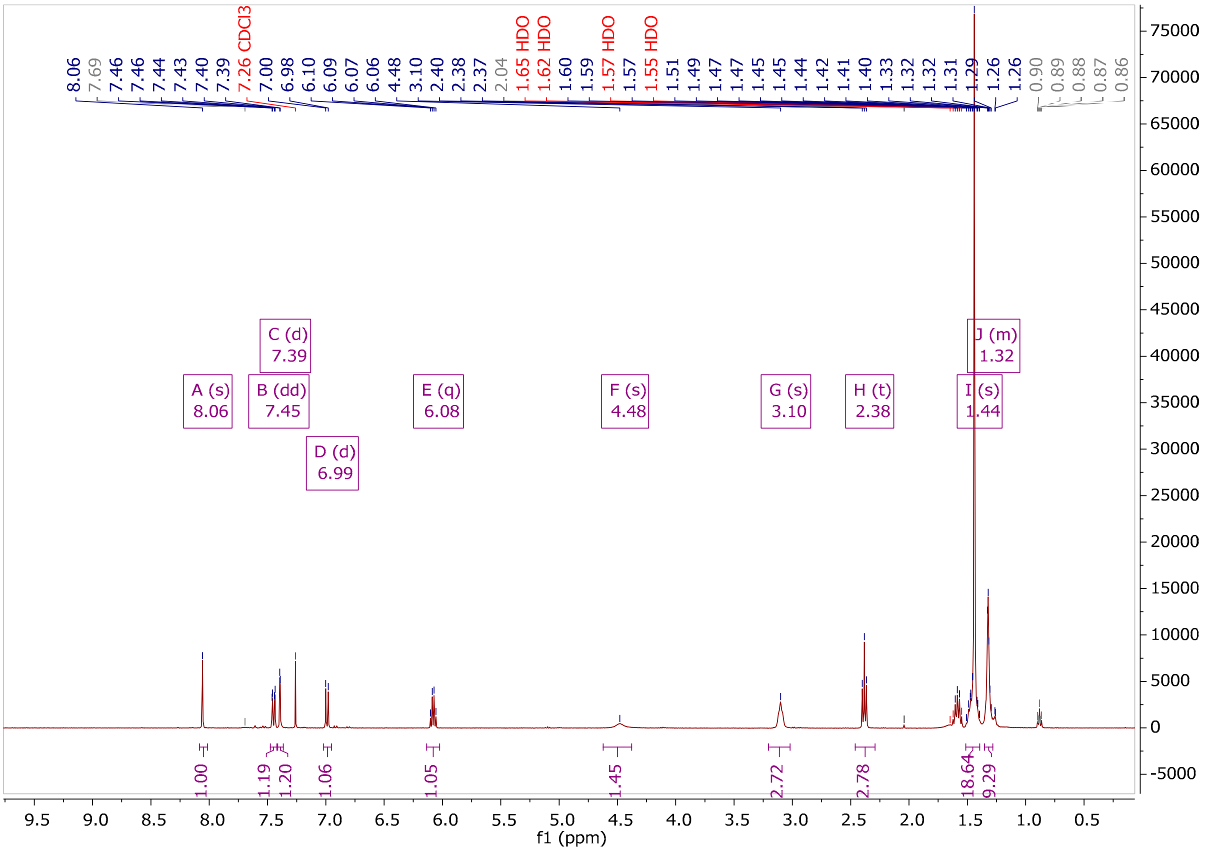
8.06
7.69
7.46
7.46
7.44
7.43
7.40
7.39
7.26 CDCl3
7.00
6.98
6.10
6.09
6.07
6.06
4.48
3.10
2.40
2.38
2.37
2.04
1.65 HDO
1.62 HDO
1.60
1.59
1.57 HDO
1.57
1.55 HDO
1.51
1.49
1.47
1.47
1.45
1.45
1.44
1.42
1.41
1.40
1.33
1.32
1.32
1.31
1.29
1.26
0.90
0.89
0.88
0.87
0.86
A (s)
8.06
B (dd)
7.45
C (d)
7.39
D (d)
6.99
E (q)
6.08
F (s)
4.48
G (s)
3.10
H (t)
2.38
I (s)
1.44
J (m)
1.32
1.00
1.19
1.20
1.06
1.05
1.45
2.72
2.78
18.64
9.29
f1 (ppm)

Compound 35

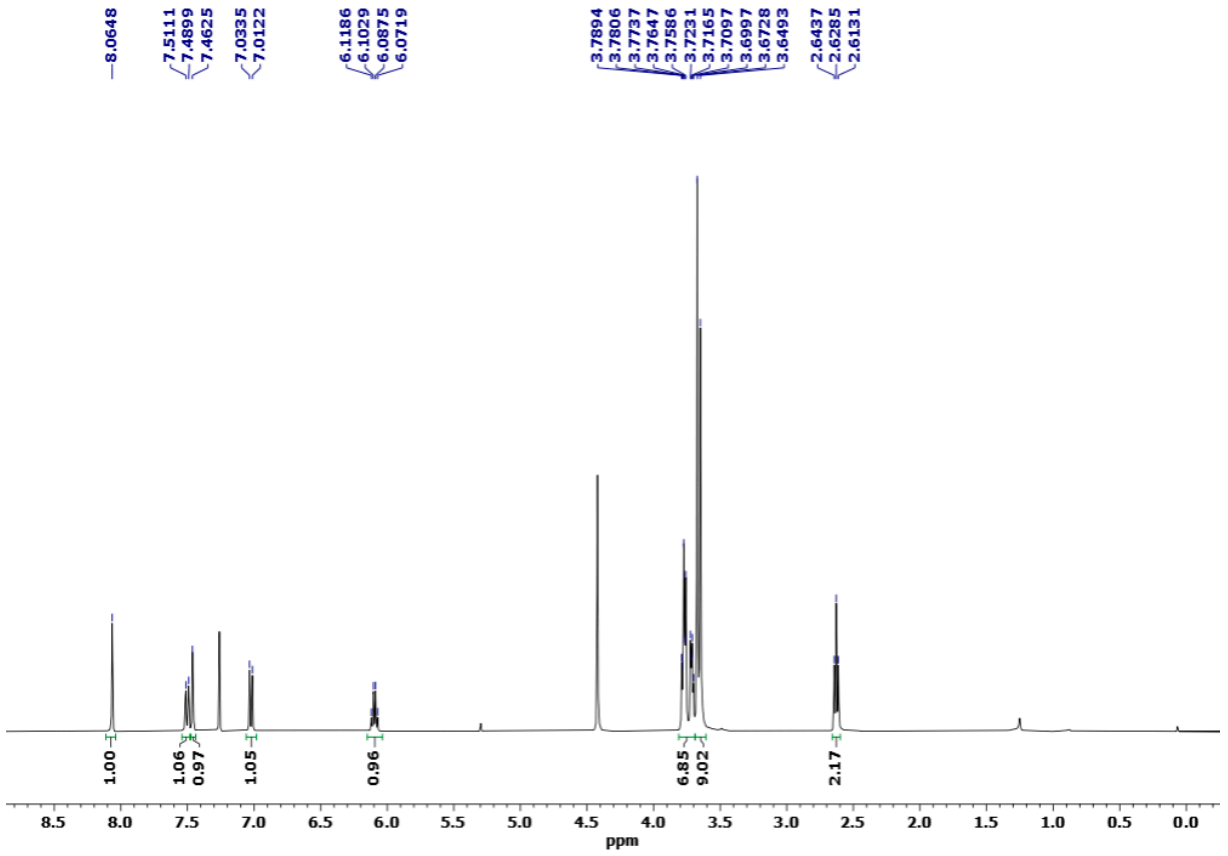

Elemental Composition Report

Single Mass Analysis
Tolerance = 5.0 mDa / DBE: min = -1.5, max = 100.0
Element prediction: Off
Number of isotope peaks used for i-FIT = 3

Monoisotopic Mass, Even Electron Ions
85 formula(e) evaluated with 1 results within limits (up to 50 closest results for each mass)
Elements Used:
C: 0-200 H: 0-200 N: 1-1 O: 0-20 F: 3-3 23Na: 1-1
JYH-10NOV22-557 270 (4.584) AM2 (Ar,25000.0,0.00,0.00); ABS
TOF MS ES+

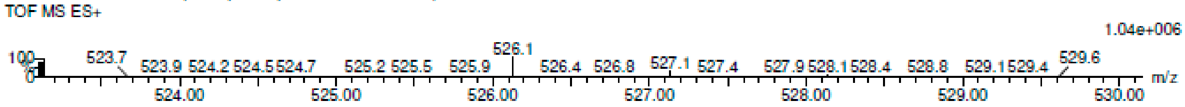

Minimum:				-1.5				
Maximum:		5.0	5.0	100.0				
Mass	Calc. Mass	mDa	PPM	DBE	i-FIT	Norm	Conf(%)	Formula
526.1307	526.1301	0.6	1.1	9.5	307.6	n/a	n/a	C22 H24 N O9 F3 23Na

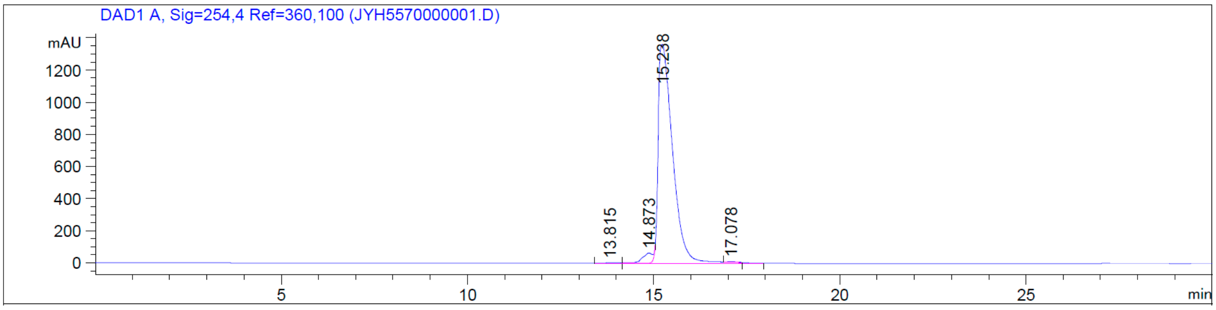
DAD1 A, Sig=254,4 Ref=360,100 (JYH5570000001.D)
mAU
13.815
14.873
15.238
17.078
min

Compound 21

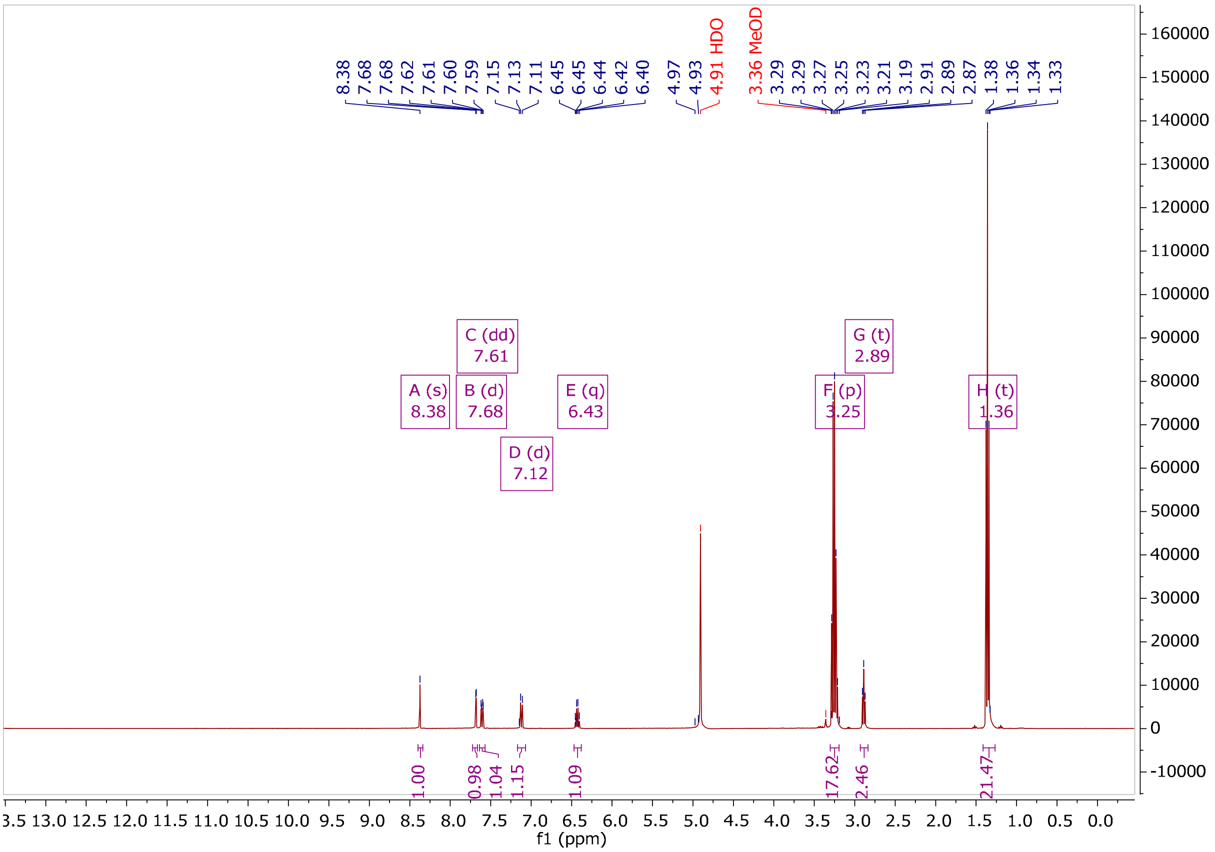
8.38 7.68 7.68 7.62 7.61 7.60 7.59 7.15 7.13 7.11 6.45 6.45 6.44 6.42 6.40 4.97 4.93
4.91 HDO
3.36 MeOD
3.29 3.29 3.27 3.25 3.23 3.21 3.19 2.91 2.89 2.87 1.38 1.36 1.34 1.33
A (s) 8.38
B (d) 7.68
C (dd) 7.61
D (d) 7.12
E (q) 6.43
F (p) 3.25
G (t) 2.89
H (t) 1.36
1.00
0.98
1.04
1.15
1.09
17.62
2.46
21.47
f1 (ppm)

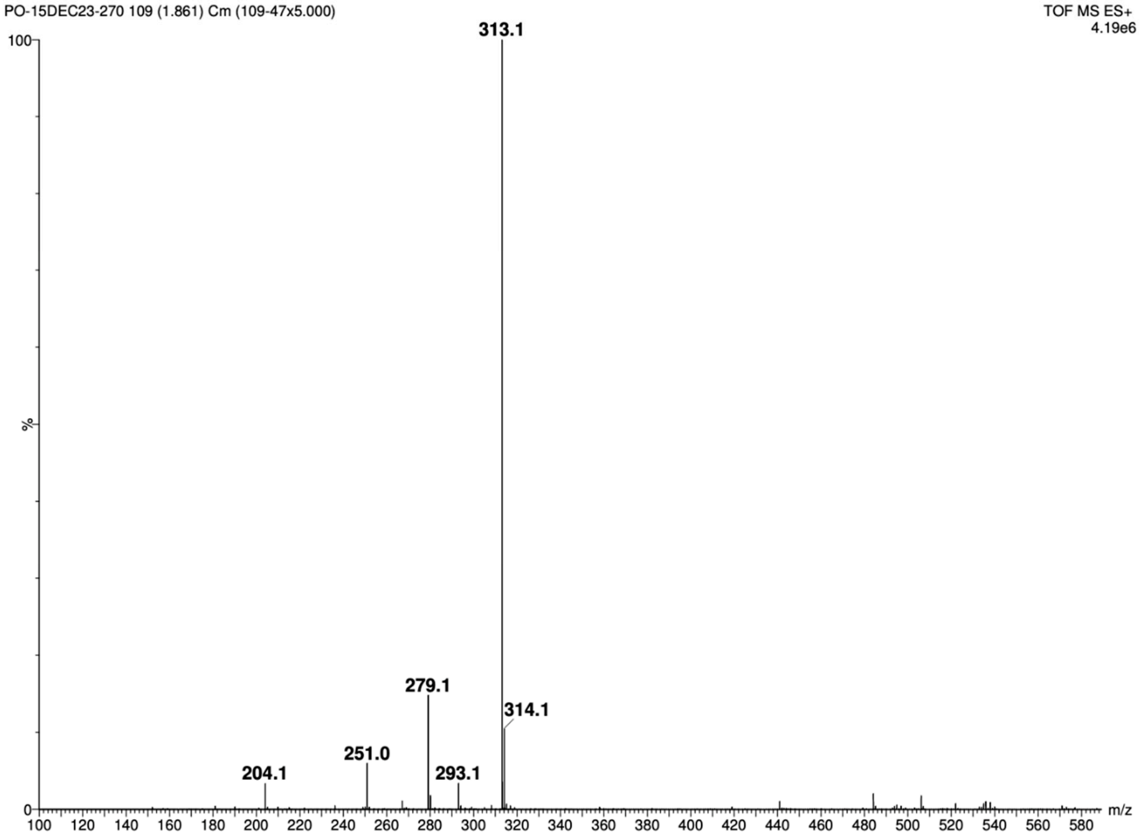
PO-15DEC23-270 109 (1.861) Cm (109-47x5.000)
TOF MS ES+
4.19e6
313.1
279.1
314.1
251.0
204.1
293.1
100
%
0
m/z
100 120 140 160 180 200 220 240 260 280 300 320 340 360 380 400 420 440 460 480 500 520 540 560 580

Compound 23

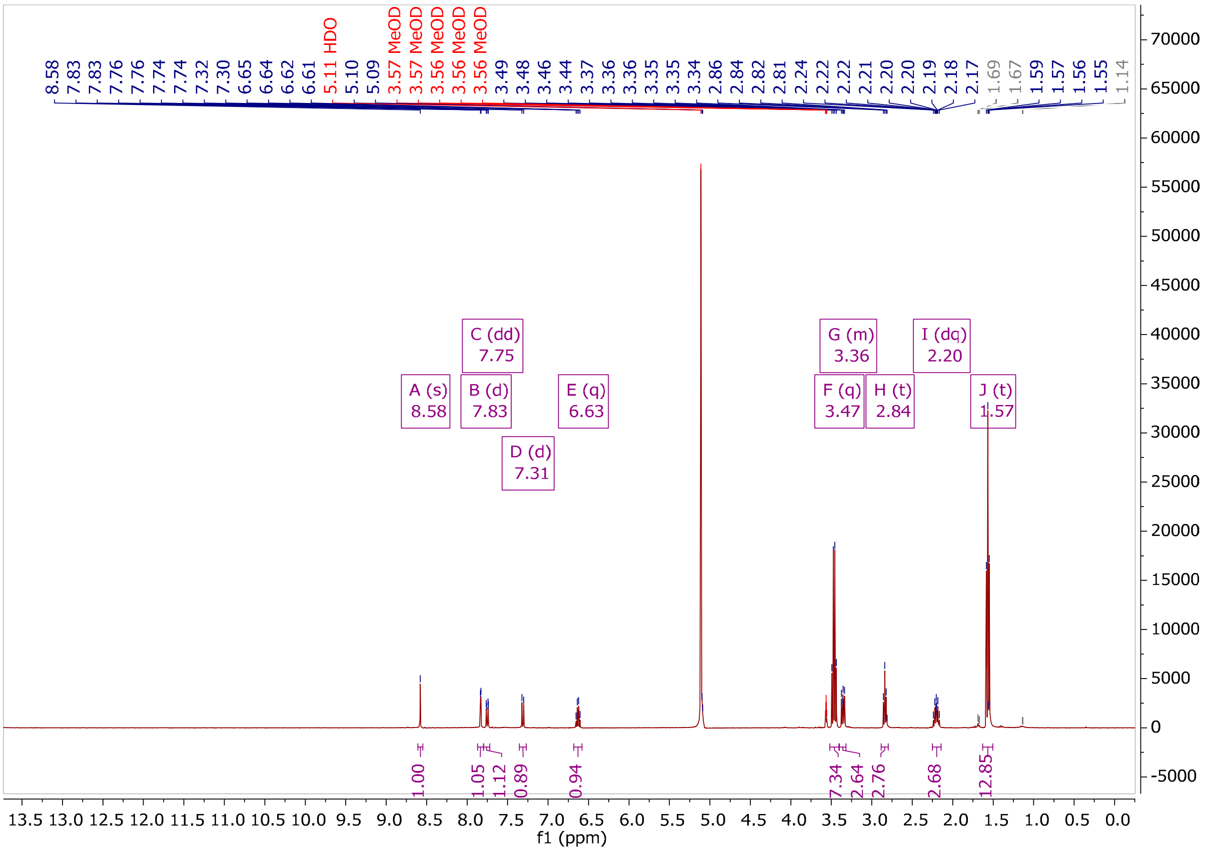
8.58
7.83
7.83
7.76
7.76
7.74
7.74
7.32
7.30
6.65
6.64
6.62
6.61
5.11 HDO
5.10
5.09
3.57 MeOD
3.57 MeOD
3.56 MeOD
3.56 MeOD
3.56 MeOD
3.49
3.48
3.46
3.44
3.37
3.36
3.36
3.35
3.35
3.34
2.86
2.84
2.82
2.81
2.24
2.22
2.22
2.21
2.20
2.20
2.19
2.18
2.17
1.69
1.67
1.59
1.57
1.56
1.55
1.14
A (s) 8.58
B (d) 7.83
C (dd) 7.75
D (d) 7.31
E (q) 6.63
F (q) 3.47
G (m) 3.36
H (t) 2.84
I (dq) 2.20
J (t) 1.57
1.00
1.05
1.12
0.89
0.94
7.34
2.64
2.76
2.68
12.85
f1 (ppm)

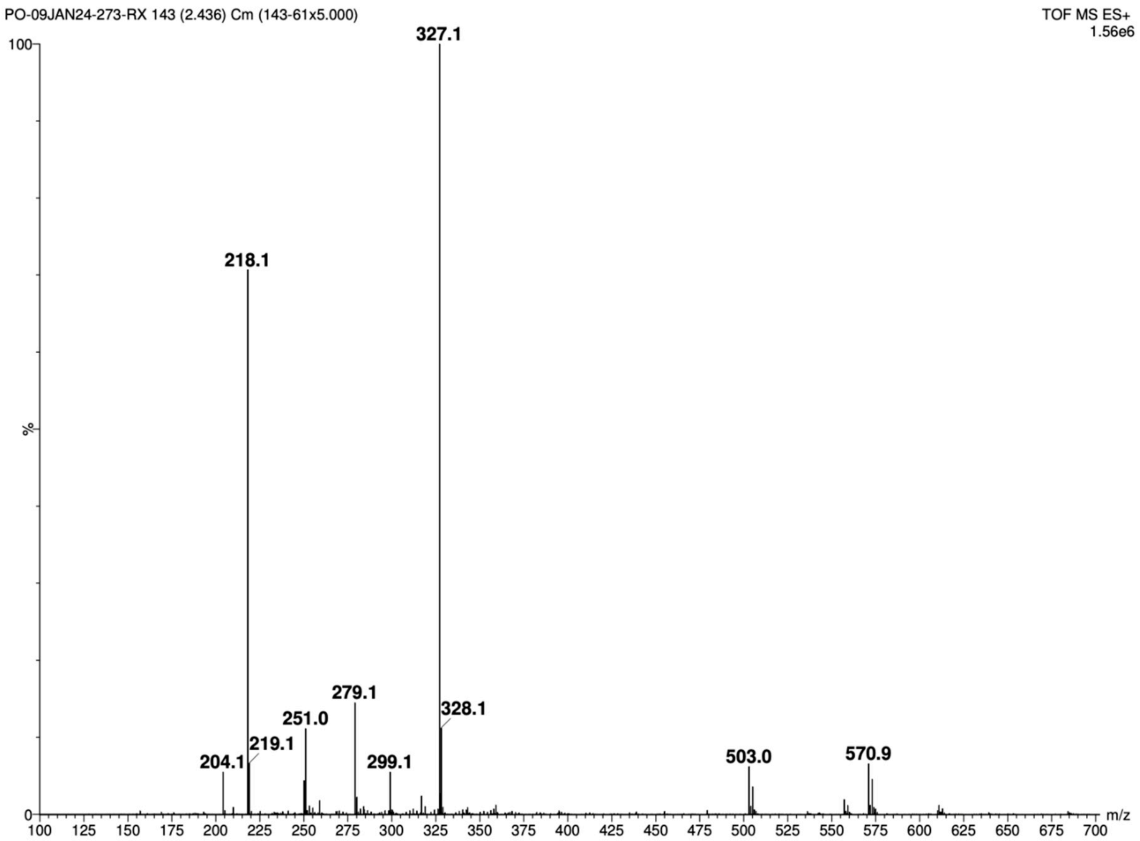

PO-09JAN24-273-RX 143 (2.436) Cm (143-61x5.000)
TOF MS ES+
1.56e6
327.1
218.1
279.1
328.1
251.0
219.1
204.1
299.1
503.0
570.9
100
%
0
100 125 150 175 200 225 250 275 300 325 350 375 400 425 450 475 500 525 550 575 600 625 650 675 700
m/z

Compound 25

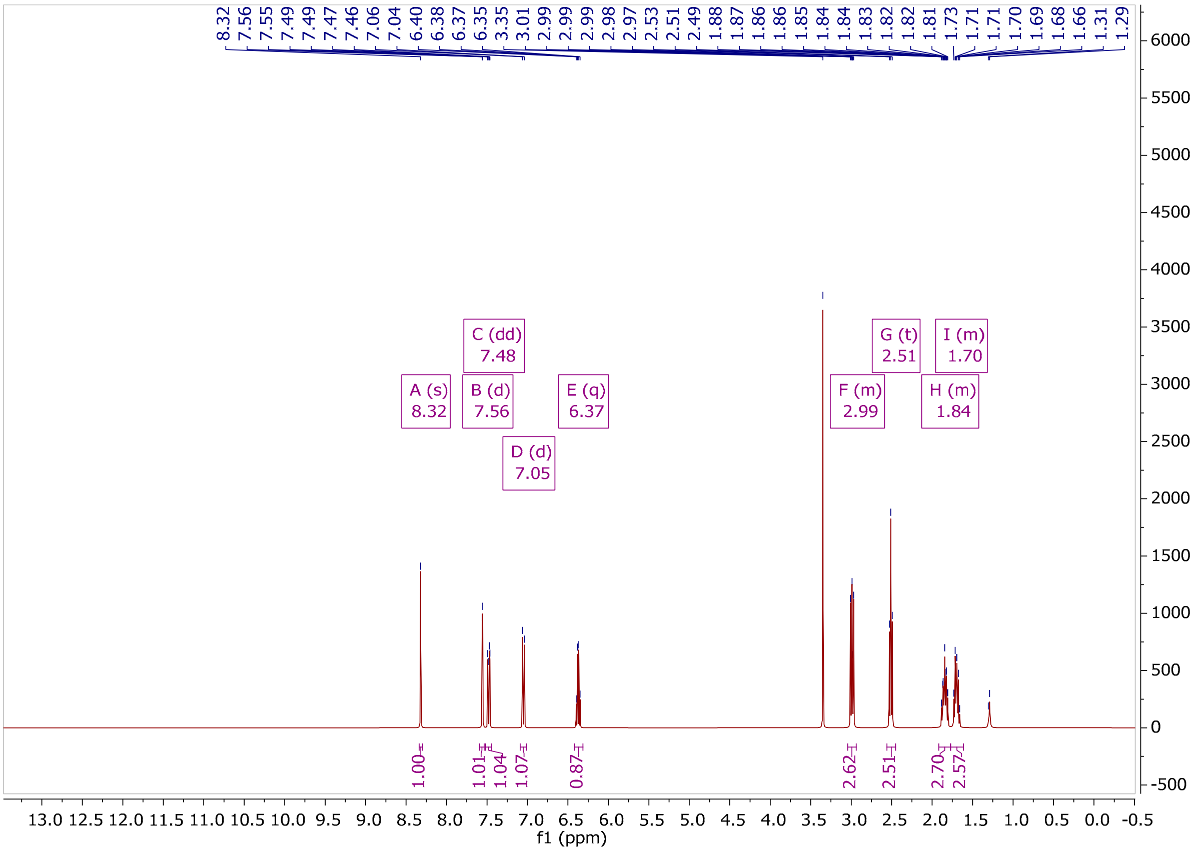

8.32 7.56 7.55 7.49 7.49 7.47 7.46 7.06 7.04 6.40 6.38 6.37 6.35 3.35 3.01 2.99 2.99 2.99 2.98 2.97 2.53 2.51 2.49 1.88 1.87 1.86 1.86 1.85 1.84 1.84 1.83 1.82 1.82 1.81 1.73 1.71 1.71 1.70 1.69 1.68 1.66 1.31 1.29
A (s) 8.32
B (d) 7.56
C (dd) 7.48
D (d) 7.05
E (q) 6.37
F (m) 2.99
G (t) 2.51
H (m) 1.84
I (m) 1.70
1.00
1.01
1.04
1.07
0.87
2.62
2.51
2.70
2.57
f1 (ppm)

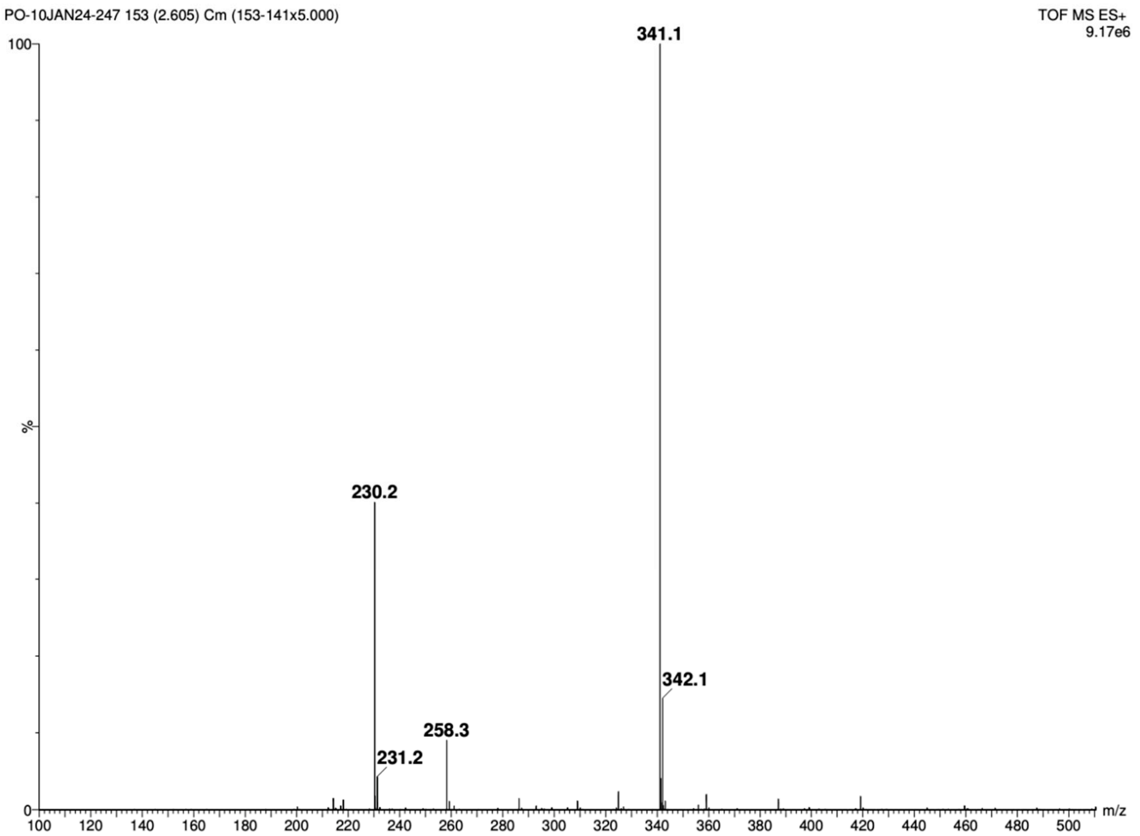
PO-10JAN24-247 153 (2.605) Cm (153-141x5.000)
TOF MS ES+
9.17e6
341.1
230.2
342.1
258.3
231.2
100
%
0
m/z
100 120 140 160 180 200 220 240 260 280 300 320 340 360 380 400 420 440 460 480 500

Compound 27

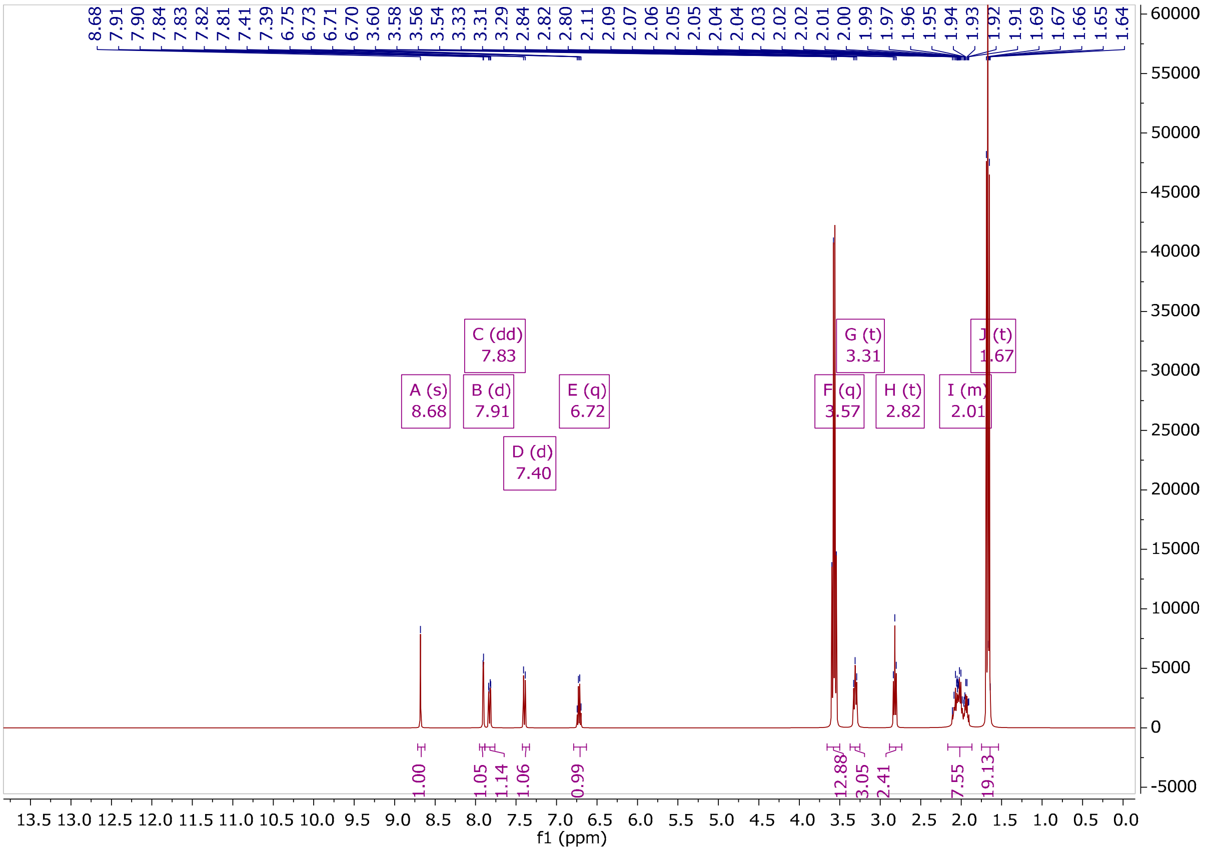
8.68
7.91
7.90
7.84
7.83
7.82
7.81
7.41
7.39
6.75
6.73
6.71
6.70
3.60
3.58
3.56
3.54
3.33
3.31
3.29
2.84
2.82
2.80
2.11
2.09
2.07
2.06
2.05
2.05
2.04
2.04
2.03
2.02
2.02
2.01
2.00
1.99
1.97
1.96
1.95
1.94
1.93
1.92
1.91
1.69
1.67
1.66
1.65
1.64
A (s) 8.68
B (d) 7.91
C (dd) 7.83
D (d) 7.40
E (q) 6.72
F (q) 3.57
G (t) 3.31
H (t) 2.82
I (m) 2.01
J (t) 1.67
1.00
1.05
1.14
1.06
0.99
12.88
3.05
2.41
7.55
19.13
f1 (ppm)

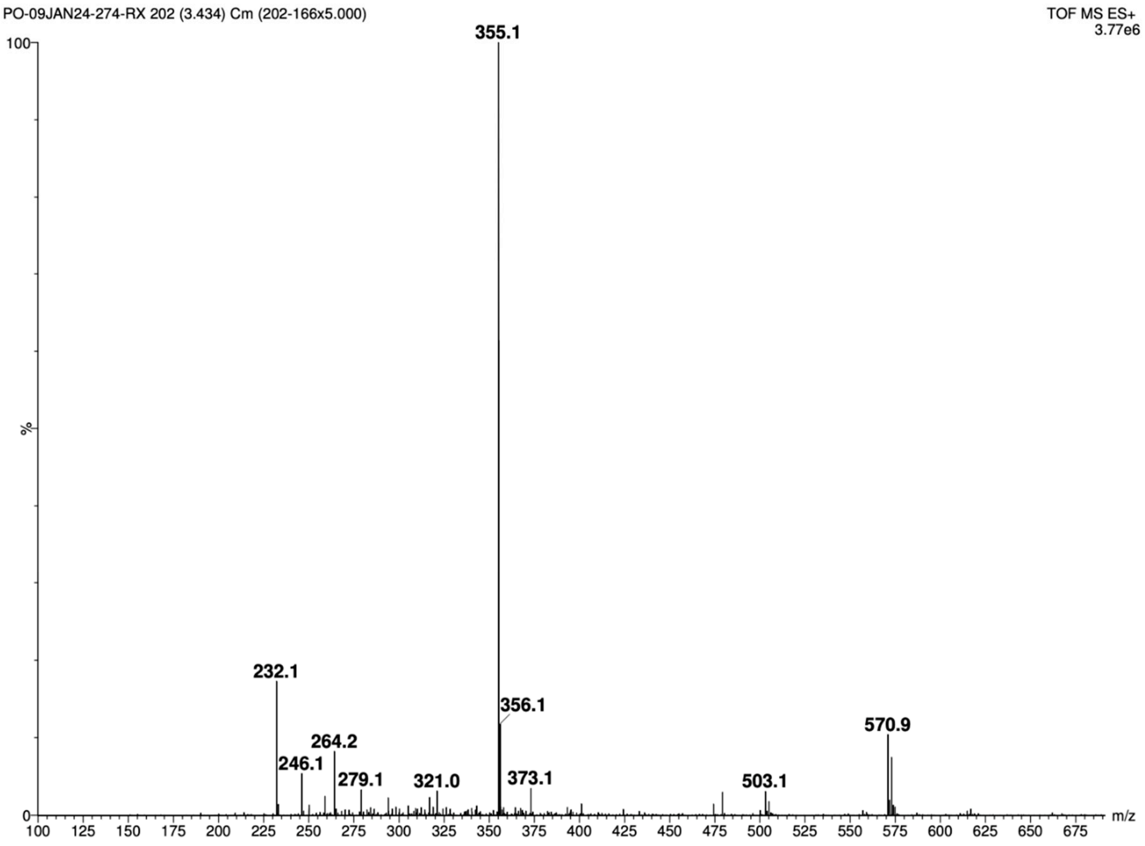

PO-09JAN24-274-RX 202 (3.434) Cm (202-166x5.000)
TOF MS ES+
3.77e6
355.1
232.1
356.1
264.2
246.1
279.1
321.0
373.1
570.9
503.1
%
m/z

Compound 29

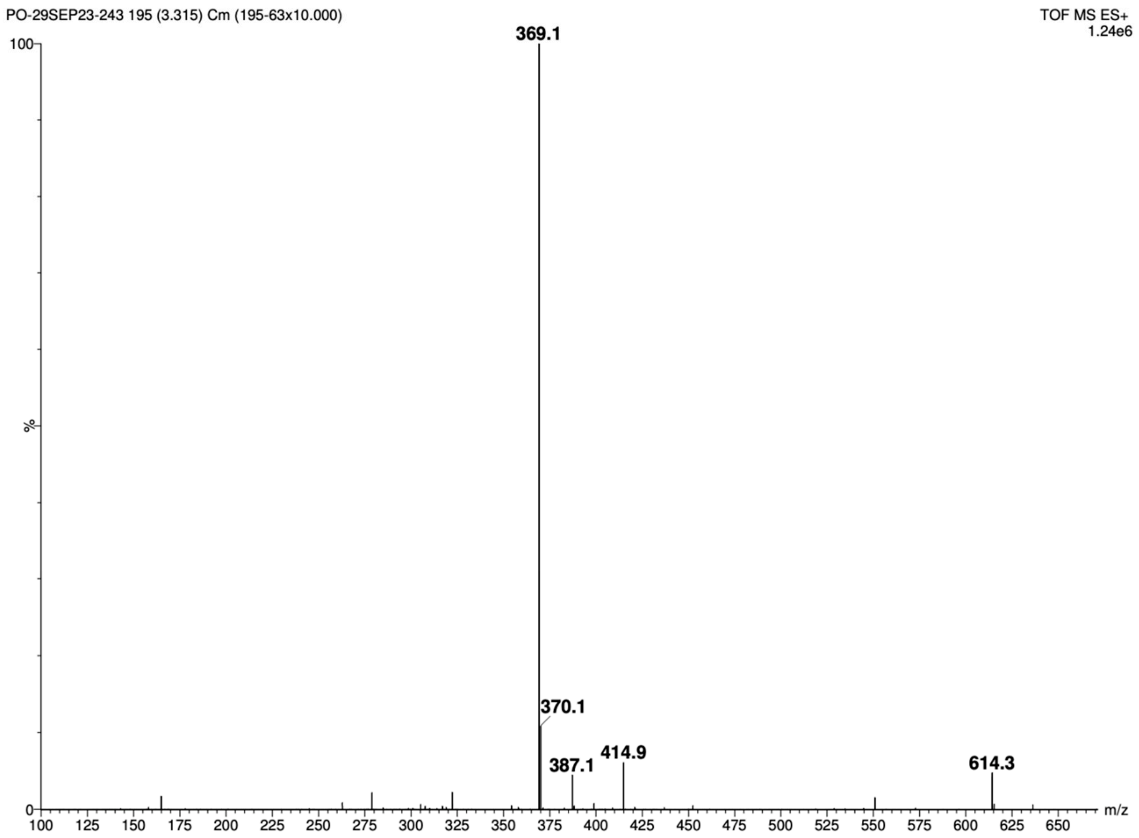
PO-29SEP23-243 195 (3.315) Cm (195-63x10.000)
TOF MS ES+
1.24e6
369.1
370.1
387.1
414.9
614.3
100
%
0
100 125 150 175 200 225 250 275 300 325 350 375 400 425 450 475 500 525 550 575 600 625 650
m/z

Compound 31

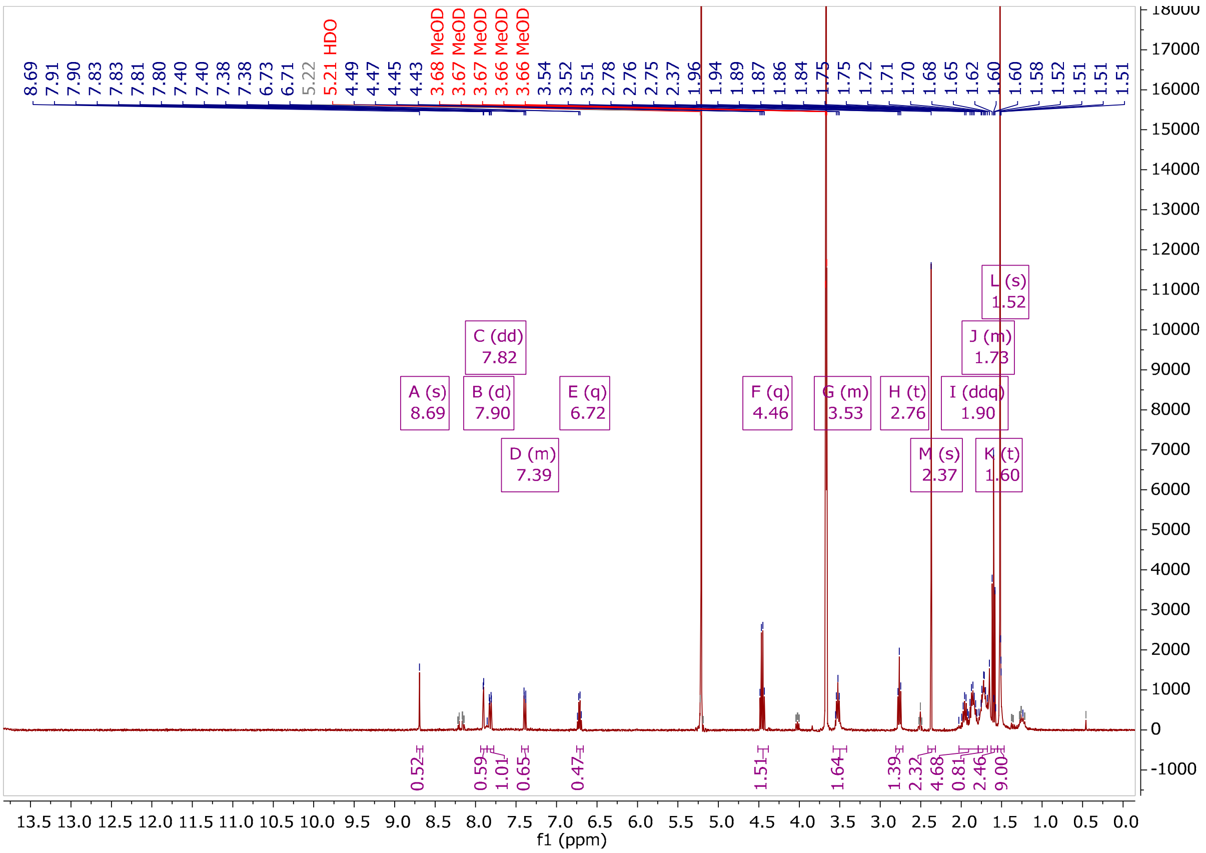

8.69 7.91 7.90 7.83 7.83 7.81 7.80 7.40 7.40 7.38 7.38 6.73 6.71 5.22
5.21 HDO
4.49 4.47 4.45 4.43
3.68 MeOD 3.67 MeOD 3.67 MeOD 3.66 MeOD 3.66 MeOD
3.54 3.52 3.51 2.78 2.76 2.75 2.37 1.96 1.94 1.89 1.87 1.86 1.84 1.75 1.75 1.72 1.71 1.70 1.68 1.65 1.62 1.60 1.60 1.58 1.52 1.51 1.51 1.51
A (s) 8.69
B (d) 7.90
C (dd) 7.82
D (m) 7.39
E (q) 6.72
F (q) 4.46
G (m) 3.53
H (t) 2.76
I (ddq) 1.90
J (m) 1.73
K (t) 1.60
L (s) 1.52
M (s) 2.37
0.52 0.59 1.01 0.65 0.47 1.51 1.64 1.39 2.32 4.68 0.81 2.46 9.00
f1 (ppm)

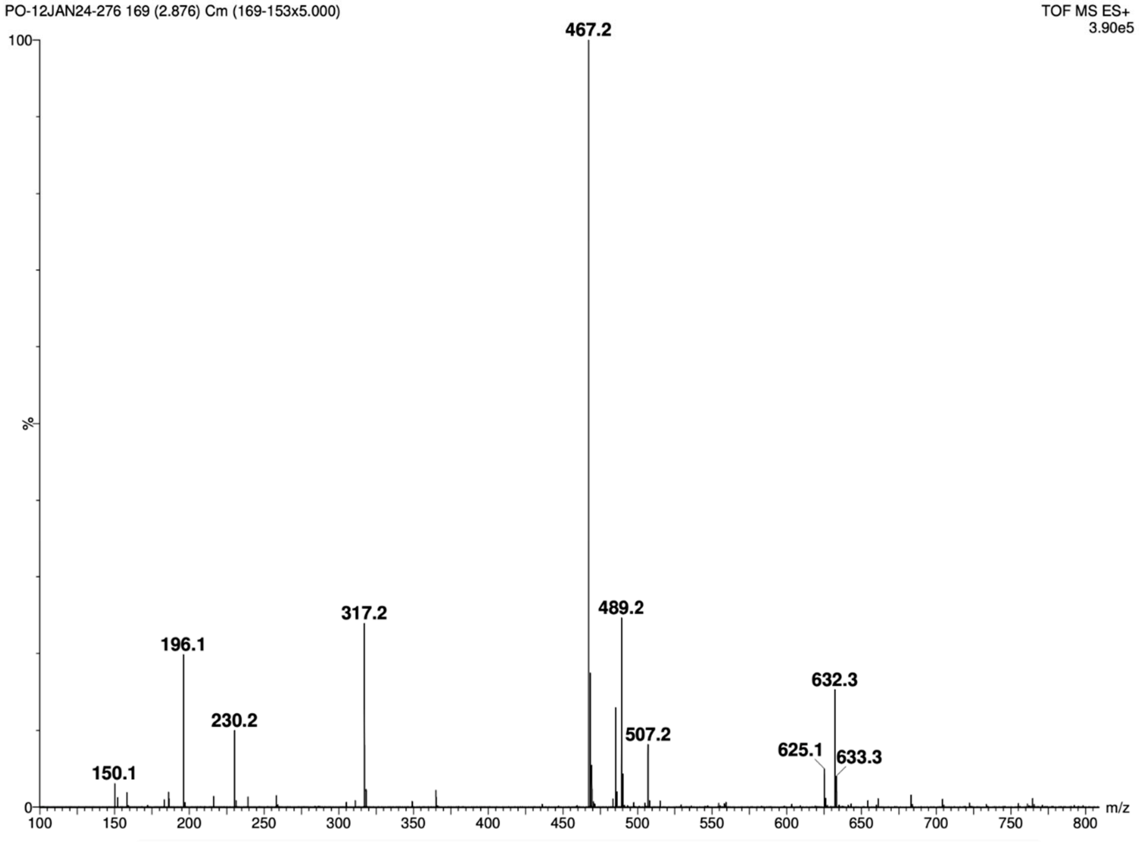
PO-12JAN24-276 169 (2.876) Cm (169-153x5.000)
TOF MS ES+
3.90e5
467.2
489.2
317.2
196.1
230.2
150.1
507.2
632.3
625.1
633.3
%
m/z
100
0
100
150
200
250
300
350
400
450
500
550
600
650
700
750
800

Compound 32

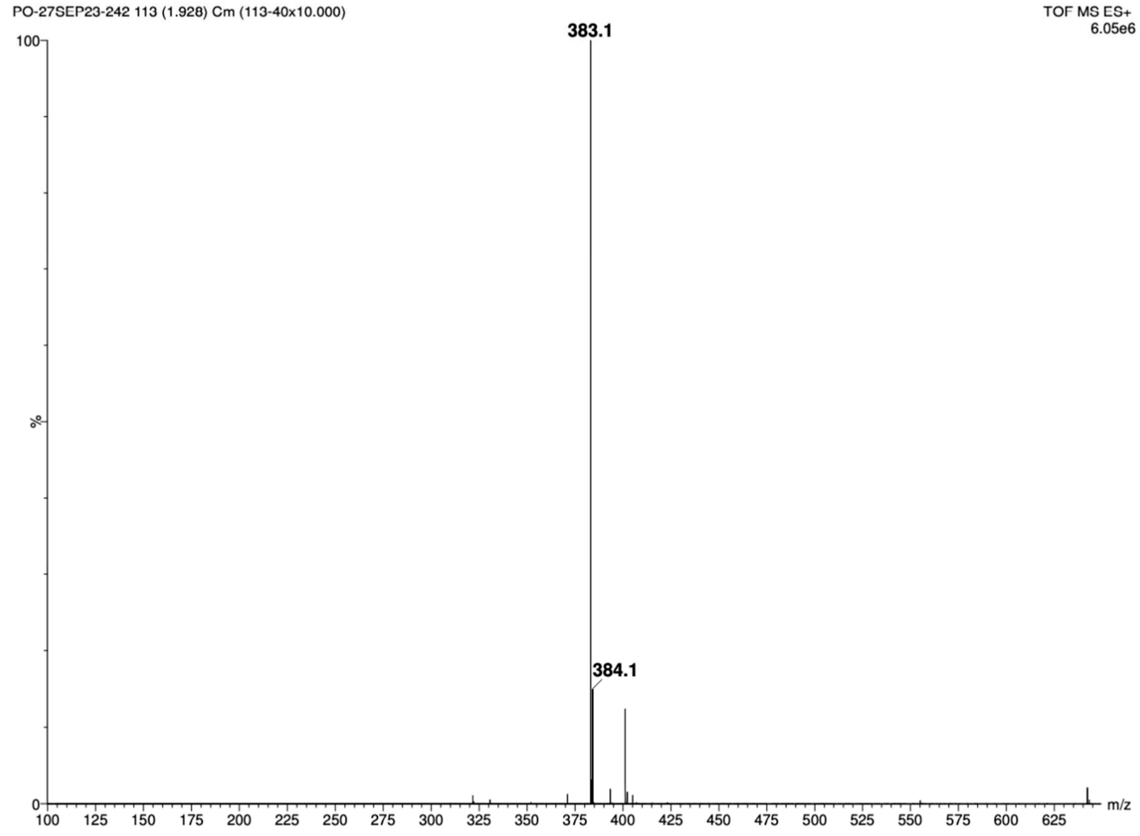
PO-27SEP23-242 113 (1.928) Cm (113-40x10.000)
TOF MS ES+
6.05e6
383.1
384.1
100
%
0
m/z
100 125 150 175 200 225 250 275 300 325 350 375 400 425 450 475 500 525 550 575 600 625

Figure S2. Dependence of affinity 6 position derivatives on chain length and terminal group (data from Table 1, compounds **19** – **30**, **32** – **34**).

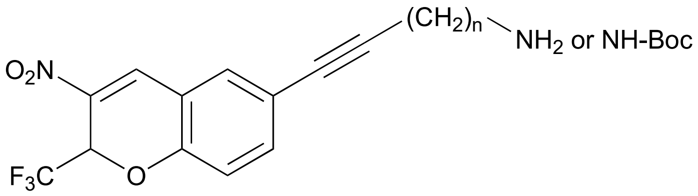

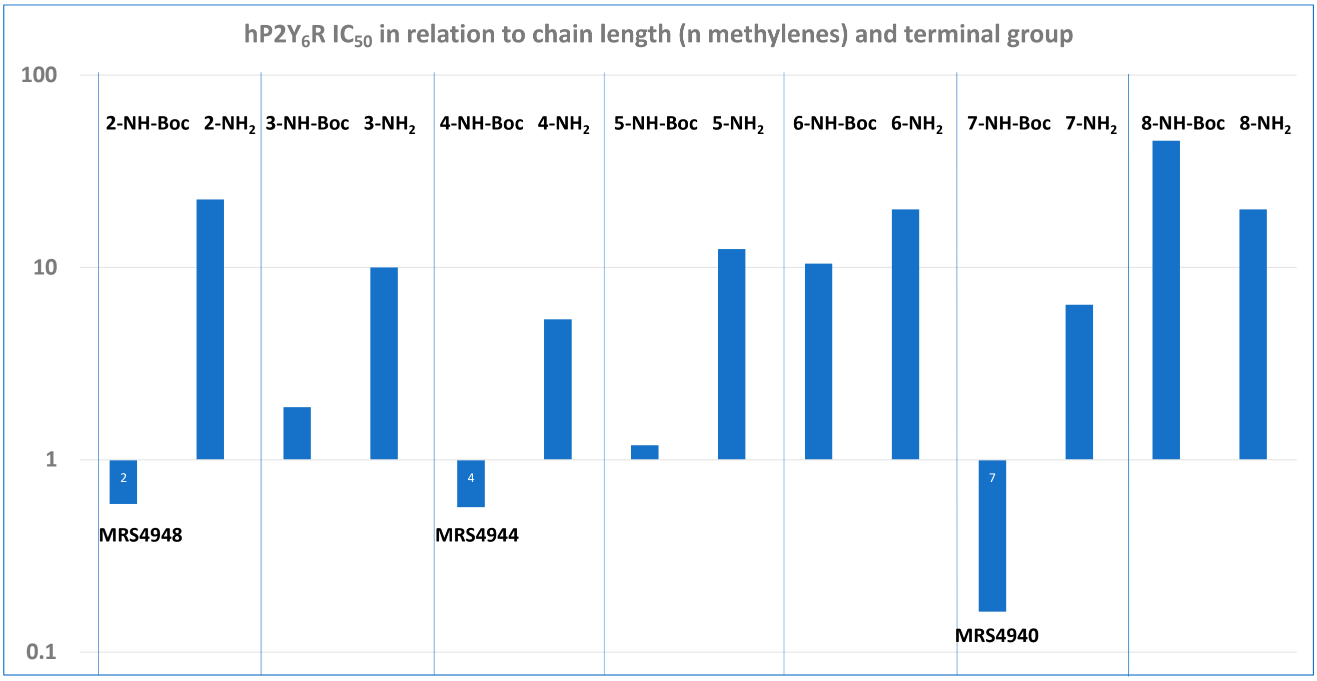

Table S1. StarDrop (Optibrium Ltd., Version 7.4.0) calculation of ADMET properties, as described in Segall, 2012 [2].

	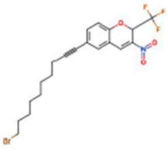	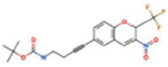	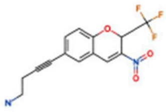	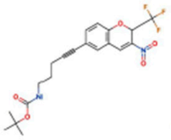	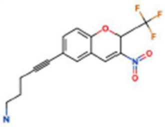	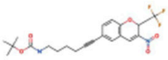
**compound #**	**19**	**20**	**21**	**22**	**23**	**24**
IC50 (nM) at hP2Y6R	14	0.587	22.6	1.87	9.98	0.568
logS	-0.01423	0.6787	2.317	0.5072	2.14	0.3444
logS @ pH7.4	-0.01423	0.6787	2.378	0.5072	2.304	0.3444
logD	6.055	4.602	1.093	4.886	1.279	5.161
2C9 pKi	4.929	5.086	4.84	5.058	4.815	4.991
hERG pIC50	6.183	5.288	4.823	5.452	4.993	5.595
BBB log([brain]:[blood])	0.385	-0.1305	-0.1351	-0.1178	-0.1135	-0.1062
BBB category	+	-	-	-	-	-
HIA category	+	+	+	+	+	+
P-gp category	no	yes	no	yes	no	yes
2D6 affinity category	high	high	high	high	high	high
PPB90 category	high	high	low	high	low	high
logP	6.055	4.602	3.05	4.886	3.347	5.161
MW	460.3	412.4	312.2	426.4	326.3	440.4
HBD	0	1	1	1	1	1
HBA	4	7	5	7	5	7
TPSA	55.05	93.38	81.07	93.38	81.07	93.38
Flexibility	0.3793	0.3	0.2174	0.3226	0.25	0.3438
Rotatable Bonds	11	9	5	10	6	11
Most Acidic pKa	N/A	N/A	N/A	N/A	N/A	N/A
Most Basic pKa	N/A	N/A	8.7	N/A	9	N/A
All pKas	N/A	N/A	Basic pKa 1: 8.7	N/A	Basic pKa 1: 9.0	N/A

	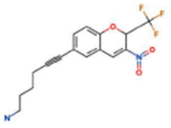	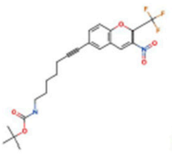	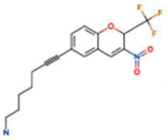	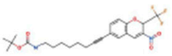	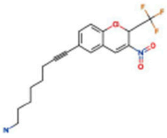	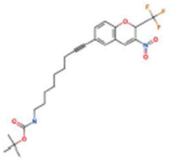
**compound #**	**25**	**26**	**27**	**28**	**29**	**30**
IC50 (nM) at hP2Y6R	5.39	1.19	12.4	10.5	20	0.162
logS	1.963	0.1631	1.726	0.02893	1.513	-0.06397
logS @ pH7.4	2.181	0.1631	2.039	0.02893	1.904	-0.06397
logD	1.501	5.454	1.683	5.66	1.826	5.838
2C9 pKi	4.962	5.1	4.981	4.895	4.795	4.993
hERG pIC50	5.141	5.736	5.292	5.87	5.435	6
BBB log([brain]:[blood])	-0.09278	-0.09363	-0.06666	-0.08758	-0.04897	-0.08371
BBB category	-	-	-	-	-	-
HIA category	+	+	+	+	+	+
P-gp category	no	yes	no	yes	no	yes
2D6 affinity category	high	high	high	high	high	high
PPB90 category	low	high	low	high	high	high
logP	3.637	5.454	3.986	5.66	4.255	5.838
MW	340.3	454.4	354.3	468.5	368.4	482.5
HBD	1	1	1	1	1	1
HBA	5	7	5	7	5	7
TPSA	81.07	93.38	81.07	93.38	81.07	93.38
Flexibility	0.28	0.3636	0.3077	0.3824	0.3333	0.4
Rotatable Bonds	7	12	8	13	9	14
Most Acidic pKa	N/A	N/A	N/A	N/A	N/A	N/A
Most Basic pKa	9	N/A	8.9	N/A	8.7	N/A
All pKas	Basic pKa 1: 9.0	N/A	Basic pKa 1: 8.9	N/A	Basic pKa 1: 8.7	N/A

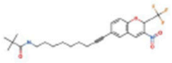
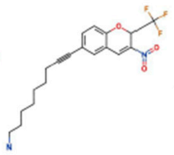
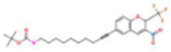
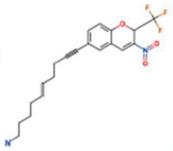
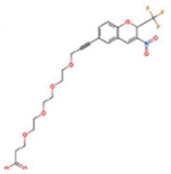
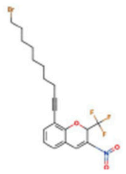

compound #	31	32	33	34	35	36
IC50 (nM) at hP2Y6R	17.3	6.37	45.4	20	63.3	4.79
logS	0.04169	1.33	-0.302	1.177	1.426	-0.01423
logS @ pH7.4	0.04169	1.771	-0.302	1.635	2.529	-0.01423
logD	5.693	1.945	6.257	2.056	0.9429	6.055
2C9 pKi	4.934	4.773	4.946	4.839	4.738	4.927
hERG pIC50	5.642	5.574	6.406	5.709	4.654	6.126
BBB log([brain]:[blood])	-0.1721	-0.03556	0.1611	-0.02383	-1.425	0.3952
BBB category	-	-	-	-	-	+
HIA category	+	+	+	+	-	+
P-gp category	yes	no	yes	yes	yes	no
2D6 affinity category	high	high	high	high	high	high
PPB90 category	high	high	high	high	high	high
logP	5.693	4.485	6.257	4.702	2.656	6.055
MW	466.5	382.4	495.5	396.4	503.4	460.3
HBD	1	1	0	1	1	0
HBA	6	5	7	5	10	4
TPSA	84.15	81.07	81.35	81.07	129.3	55.05
Flexibility	0.3824	0.3571	0.4167	0.3793	0.4722	0.3793
Rotatable Bonds	13	10	15	11	17	11
Most Acidic pKa	N/A	N/A	N/A	N/A	2.9	N/A
Most Basic pKa	N/A	8.7	N/A	8.7	N/A	N/A
All pKas	N/A	Basic pKa 1: 8.7	N/A	Basic pKa 1: 8.7	Acidic pKa 1: 2.9	N/A

	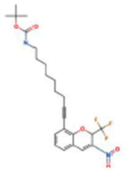	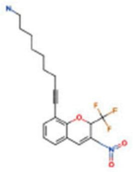	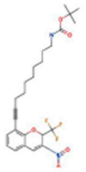	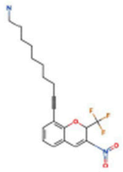	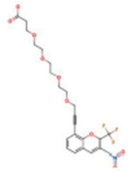
**compound #**	**37**	**38**	**39**	**40**	**41**
IC50 (nM) at hP2Y6R	4.96	13	23.8	34.8	> 50
logS	-0.06397	1.33	-0.1229	1.177	1.426
logS @ pH7.4	-0.06397	1.693	-0.1229	1.558	2.421
logD	5.838	1.969	6.012	2.077	0.9475
2C9 pKi	4.968	4.796	5.032	4.852	4.698
hERG pIC50	5.944	5.516	6.071	5.652	4.591
BBB log([brain]:[blood])	-0.07131	-0.02577	-0.06802	-0.01402	-1.413
BBB category	-	-	-	-	-
HIA category	+	+	+	+	-
P-gp category	yes	no	yes	yes	yes
2D6 affinity category	high	high	high	high	high
PPB90 category	high	high	high	high	high
logP	5.838	4.485	6.012	4.702	2.656
MW	482.5	382.4	496.5	396.4	503.4
HBD	1	1	1	1	1
HBA	7	5	7	5	10
TPSA	93.38	81.07	93.38	81.07	129.3
Flexibility	0.4	0.3571	0.4167	0.3793	0.4722
Rotatable Bonds	14	10	15	11	17
Most Acidic pKa	N/A	N/A	N/A	N/A	3.5
Most Basic pKa	N/A	8.9	N/A	8.5	N/A
All pKas	N/A	Basic pKa 1: 8.9	N/A	Basic pKa 1: 8.5	Acidic pKa 1: 3.5

Table S2. Off-target screening (to replace table later). We thank Dr. Bryan L. Roth (Univ. North Carolina at Chapel Hill) and National Institute of Mental Health's Psychoactive Drug Screening Program (Contract # HHSN-271-2008-00025-C) for screening data. Reference: Besnard et al. [3].
Procedures: https://pdsp.unc.edu/pdspweb/content/UNC-CH%20Protocol%20Book.pdf
Unless noted in the text, no significant interactions (<50% inhibition at 10 μM) for any of the nucleosides were found at the following sites (human unless noted): $5HT_{1A}$, $5HT_{1B}$, $5HT_{1D}$, $5HT_{1E}$, $5HT_{2A}$, $5HT_{2B}$, $5HT_{2C}$, $5HT_3$, $5HT_{5A}$, $5HT_6$, $5HT_7$, α_{1A}, α_{1B}, α_{1D}, α_{2A}, α_{2B}, α_{2C}, β_1, β_2, β_3, BZP rat brain site, D_1, D_2, D_3, D_4, D_5, $GABA_A$, H_1, H_2, H_3, H_4, M_1, M_2, M_5, δ-opioid receptor (DOR), κ-opioid receptor (KOR), μ-opioid receptor (MOR), σ_1, σ_2, DAT, NET, SERT. K_i values in μM, or % inhibition at 10 μM, are given.

Correspondence of compound numbers: **20** (MRS4948), PDSP74618; **24** (MRS4944), PDSP73669; **26** (MRS4952), PDSP74619; **30** (MRS4940), PDSP73668; **31** (MRS4955), PDSP74620; **37** (MRS4956), PDSP74621.

Compound #	PDSP #	Receptor	LogKi	Ki (nM)
30	73668	5-HT1A	> -5	> 10,000
30	73668	D3	> -5	> 10,000
30	73668	D5	-6.19	642.84
30	73668	D5	-6.04	911.38
30	73668	D2	> -5	> 10,000
30	73668	H1	-6.2	630.09
30	73668	H1	-6.44	365.51
30	73668	Alpha2A	-7.1	80.24
30	73668	Alpha2A	-7.04	90.72
30	73668	SERT	> -5	> 10,000
30	73668	KOR	-5.85	1402.81
30	73668	Sigma 1	> -5	> 10,000
24	73669	D5	-5.87	1359.88
24	73669	D5	-5.47	3415.07
24	73669	D1	-5.8	1602.14
24	73669	H1	-5.84	1431.86
24	73669	H1	-6.2	631.25
24	73669	Alpha2A	-6.83	147.98
24	73669	Alpha2A	-6.78	167.61
24	73669	5-HT1D	-5.49	3262.87
24	73669	5-HT1D	-5.52	3037.39

Cell viability (MTT) assay in the presence of selected P2Y$_6$R antagonists (data for Figure 5). See main text for procedure.

HeLa cells

MRS4830

Conc. (µM), 0	0.1	0.5	1	5	10
% survival, 100	94	91	93	87	83

2b, MRS4841

Conc. (µM), 0	0.1	0.5	1	5	10
% survival, 100	87	86	87	84	78

30, MRS4940

Conc. (µM), 0	0.05	0.1	0.5	1
% survival, 100	94	94	87	71

MRS2693

Conc. (µM), 0	1	5	10
% survival, 100	105	106	104

P2Y$_6$R-astrocytoma

MRS4830

Conc. (µM), 0	0.1	0.5	1	5	10
% survival, 100	100	102	101	96	89

2b, MRS4841

Conc. (µM), 0	0.1	0.5	1	5	10
% survival, 100	104	104	105	98	88

30, MRS4940

Conc. (µM), 0	0.05	0.1	0.5	1
% survival, 100	98	98	83	77

MRS2693

Conc. (µM), 0	1	5	10
% survival, 100	98	100	101

References:

[1] Jung, Y.H.; Shah, Q.; Lewicki, S.A.; Pramanik, A.; Gopinatth, V.; Pelletier, J.; Sévigny, J.; Iqbal, J.; Jacobson, K.A. Synthesis and pharmacological characterization of multiply substituted 2H-chromene derivatives as $P2Y_6$ receptor antagonists. *Bioorg. Med. Chem. Lett.* **2022**, *75*, 128981. https://doi.org/10.1016/j.bmcl.2022.128981

[2] Segall M.D. Multi-parameter optimization: identifying high quality compounds with a balance of properties. *Curr. Pharmaceut. Des.* **2012**, *18*(9), 1292–1310. https://doi.org/10.2174/138161212799436430

[3] Besnard, J.; Ruda, G. F.; Setola, V.; Abecassis, K.; Rodriguiz, R. M.; Huang, X. P.; Norval, S.; Sassano, M. F.; Shin, A. I.; Webster, L. A.; Simeons, F. R.; Stojanovski, L.; Prat, A.; Seidah, N. G.; Constam, D. B.; Bickerton, G. R.; Read, K. D.; Wetsel, W. C.; Gilbert, I. H.; Roth, B. L.; Hopkins, A. L. Automated design of ligands to polypharmacological profiles. *Nature* **2012**, *492*, 215–220.
